# Seven New and Two Known Lipopeptides as well as Five Known Polyketides: The Activated Production of Silent Metabolites in a Marine-Derived Fungus by Chemical Mutagenesis Strategy Using Diethyl Sulphate

**DOI:** 10.3390/md12041815

**Published:** 2014-03-28

**Authors:** Chang-Jing Wu, Chang-Wei Li, Cheng-Bin Cui

**Affiliations:** 1 Key Laboratory of Structure-Based Drug Design & Discovery of Ministry of Education, School of Traditional Chinese Materia Medica, Shenyang Pharmaceutical University, Shenyang 110016, China; E-Mail: wucj2009@163.com; 2 Beijing Institute of Pharmacology and Toxicology, Beijing 100850, China; E-Mail: sdrlcw@126.com

**Keywords:** marine-derived fungus, lipopeptide, penicimutalide, Marfey analysis, polyketide, *Penicillium purpurogenum*, DES mutagenesis

## Abstract

AD-2-1 is an antitumor fungal mutant obtained by diethyl sulfate mutagenesis of a marine-derived *Penicillium purpurogenum* G59. The G59 strain originally did not produce any metabolites with antitumor activities in MTT assays using K562 cells. Tracing newly produced metabolites under guidance of MTT assay and TLC analysis by direct comparison with control G59 extract, seven new (**1**–**7**) and two known (**8**–**9**) lipopeptides were isolated together with five known polyketides **10**–**14** from the extract of mutant AD-2-1. Structures of the seven new compounds including their absolute configurations were determined by spectroscopic and chemical evidences and named as penicimutalides A–G (**1**–**7**). Seven known compounds were identified as fellutamide B (**8**), fellutamide C (**9**), 1′-*O*-methylaverantin (**10**), averantin (**11**), averufin (**12**), nidurufin (**13**), and sterigmatocystin (**14**). In the MTT assay, **1**–**14** inhibited several human cancer cell lines to varying extents. All the bioassays and HPLC-photodiode array detector (PDAD)-UV and HPLC-electron spray ionization (ESI)-MS analyses demonstrated that the production of **1**–**14** in the mutant AD-2-1 was caused by the activated production of silent metabolites in the original G59 fungal strain. Present results provided additional examples for effectiveness of the chemical mutagenesis strategy using diethyl sulphate mutagenesis to discover new compounds by activating silent metabolites in fungal isolates.

## 1. Introduction

Activating silent secondary metabolites of fungal isolates have attracted considerable attention and various strategies have been developed for this purpose in the past decade [1,2,3,4]. The one strain-many compounds (OSMAC) strategy [5] and chemical epigenetics methodology [6] have been widely used by microbial chemists to access cryptic secondary metabolites [7]. The ribosome engineering strategy [8] provided another simple way to activate silent pathways by introducing drug-resistance mutations in bacteria to discover new antibiotics [9]. We previously extended this strategy to fungi to activate silent metabolites by introducing drug-resistance in a bio-inactive marine-derived *Penicillium purpurogenum* G59 using dimethyl sulfoxide (DMSO) as accessorial agent [10,11]. As further extension of that work, we have just recently developed a practical strategy to discover new antitumor agents by activating silent fungal metabolite production using a modified method of diethyl sulphate (DES) mutagenesis on *P. purpurogenum* G59 [12]. Using this strategy, several new antitumor agents including some with novel structures have also been discovered [12,13]. As a continuation, we report here our recent work on another bioactive mutant AD-2-1, including the discovery of seven new lipopeptides from activated production of silent metabolites in the original G59 fungal strain.

**Figure 1:**
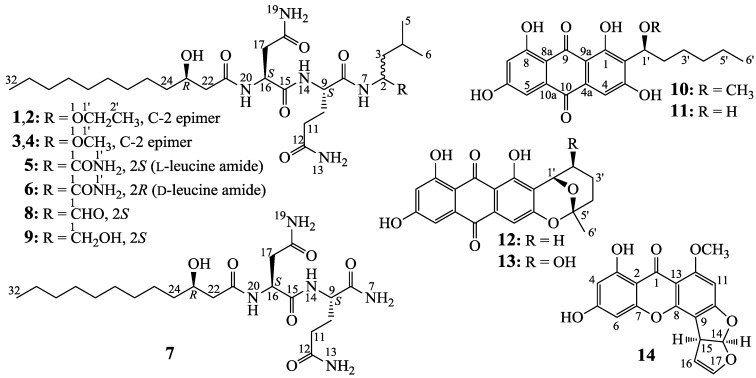
Structures of **1**–**14** newly produced by the mutant AD-2-1.

*P. purpurogenum* G59 is a marine-derived, wild-type fungal strain initially isolated by our group [14] and originally did not produce any metabolites with antitumor activity in repeated bioassay using 3-(4,5-dimethylthiazol-2-yl)-2,5-diphenyltetrazolium bromide (MTT) and K562 cells [10,11,12,13,14]. AD-2-1 was obtained as an antitumor mutant by DES mutagenesis of the strain G59 and the ethyl acetate (EtOAc) extract of its cultures inhibited K562 cells with an inhibition rate (IR%) value of 49.8% at 100 µg/mL [12]. However, the metabolites with antitumor activity in the EtOAc extract have not been investigated so far. In the present work, we thus investigated the antitumor metabolites in the EtOAc extract, which were newly produced by the mutant AD-2-1 compared to its parent G59 strain. This has resulted in the discovery of seven new lipopeptides (**1**–**7**) and the identification of two known lipopeptides (**8** and **9**) as well as five known polyketides (**10**–**14**) shown in Figure 1. All **1**–**14** inhibited several human cancer cell lines to varying extents. All the bioassays and HPLC-photodiode array detector (PDAD)-UV and HPLC-electron spray ionization (ESI)-MS analyses demonstrated that the production of **1**–**14** in the mutant AD-2-1 was caused by the activated production of silent metabolites in original fungal G59 strain.

## 2. Results and Discussion

### 2.1. Fermentation, Isolation of **1–14**, and Identification of Known Compounds **8–14**

Large-scale fermentation and extraction of the mutant AD-2-1 produced an EtOAc extract that inhibited the K562 cells with an IR% value of 64.6% at 100 µg/mL. However, the control G59 EtOAc extract that was obtained by fermentation of the G59 strain at the same time and same conditions did not inhibit the K562 cells (an IR% of 2.5% at 100 µg*/*mL). In preliminary analyses of the mutant AD-2-1 and the control G59 extracts by thin layer chromatography (TLC), lipopeptide components that contained **1**–**9** were detected as white spots by spraying distilled water to the silica gel TLC plates in the mutant extract but not in the control G59 extract. The polyketide components including **10**–**14** were also detected only in the mutant extract as spots on the silica gel TLC plates or on the polyamide film by examination under sunlight or UV lights (254 and 360 nm) or by visualization using 5% FeCl_3_ reagent, Vaughan’s reagent or 10% sulfuric acid reagent. Thus, the separation of the mutant extract was performed tracing these newly produced metabolites under the guidance of MTT assay and TLC analysis by direct comparison with the control G59 extract.

A column chromatography of the mutant extract on Sephadex LH-20 by monitoring the targeted components gave two fractions that contained the aimed lipopeptides and polyketides, respectively. The lipopeptide-containing fraction was separated by chromatographic means, including repeatedly performed preparative TLC, column chromatography, and semi-preparative HPLC, to obtain **1**–**9** shown in Figure 1. The HPLC separations were performed on a chiral, reversed-phase Click β-CD column. The polyketide-containing fraction was separated by repeated column chromatography on silica gel, polyamide, and Sephadex LH-20 to afford **10**–**14** (Figure 1).

Among the obtained compounds **1**–**14**, structures of seven new compounds including their absolute configurations were determined by spectroscopic and chemical evidences and named penicimutalides A–G (**1**–**7**). Structure determinations of these new compounds are described in detail in Section 2.2. Further, seven known compounds including two lipopeptides **8**–**9** and five polyketides **10**–**14** were identified as fellutamide B (**8**) [15], fellutamide C (**9**) [16], 1′-*O*-methylaverantin (**10**) [17], averantin (**11**) [18], averufin (**12**) [18,19], nidurufin (**13**) [18,19,20], and sterigmatocystin (**14**) [21], respectively, by their physicochemical and spectroscopic data. The identification of fellutamide B (**8**) and fellutamide C (**9**) was also based on the chemical evidences to confirm their absolute configurations.

### 2.2. Structure Determination of New Compounds **1–7**

Penicimutalide A (**1**), 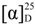 −18.0 (*c* 0.1, MeOH), and penicimutalide B (**2**), 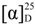 −17.0 (*c* 0.1, MeOH), were obtained as amorphous powders from MeOH, and their molecular compositions were assigned to be C_28_H_53_N_5_O_7_ by HRESIMS (measured 594.3834 [M + Na]^+^ for both **1** and **2**, calcd for C_28_H_53_N_5_O_7_Na [M + Na]^+^ 594.3843). Penicimutalides C (**3**), 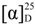 −23.7 (*c* 0.3, MeOH), and D (**4**), 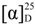 −24.8 (*c* 0.3, MeOH), amorphous powders (MeOH), were assigned the same molecular formula C_27_H_51_N_5_O_7_ (HRESIMS: measured 580.3695 [M + Na]^+^ for **3** and 580.3684 [M + Na]^+^ for **4**; calcd for C_27_H_51_N_5_O_7_Na [M + Na]^+^ 580.3686). Penicimutalides E (**5**), 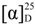 −15.0 (*c* 0.04, MeOH), and F (**6**), 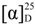 −18.8 (*c* 0.04, MeOH), amorphous powders (MeOH), had also the same molecular composition C_27_H_50_N_6_O_7_ (HRESIMS: measured 571.3819 [M + H]^+^ for **5** and 571.3805 [M + H]^+^ for **6**, calcd for C_27_H_51_N_6_O_7_ [M + H]^+^ 571.3819). Penicimutalide G (**7**), an amorphous powder (MeOH), 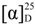 −7.5 (*c* 0.1, MeOH), was assigned the molecular formula C_21_H_39_N_5_O_6_ by HRESIMS (measured 458.2970 [M + H]^+^, calcd for C_21_H_40_N_5_O_6_ [M + H]^+^ 458.2979).

The UV spectra of **1**–**7** exhibited end absorptions (see their absorption curves in Figure S1-A2 in the Supplementary Information), and their IR spectra (see their IR spectra in the Supplementary Information) showed absorptions due to OH/NH (around 3273 and 3205 cm^−1^), CH_3_/CH_2_ (around 2930 and 2860 cm^−1^), and amide (amide I bands around 1650 cm^−1^ for amide carbonyl and amide II bands around 1535 cm^−1^ for amide H_2_NCO and/or HNCO) groups. These data, coupled with the molecular sizes and the molecular compositions of **1**–**7**, revealed that they were peptides. In the ^1^H and ^13^C NMR spectra (see their NMR spectra in the Supplementary Information), **1**–**7** showed ^1^H and ^13^C NMR signals that closely resembled the signals from the known lipopeptides **8** and **9** except several signals from leucine-derived moieties have slightly changed (Table 1 and Table 2). These NMR data indicated that **1**–**7** are all lipopeptides that resembled **8** and **9** but differed only in the leucine-derived moieties. This was further confirmed by the detailed analyses of their DEPT, ^1^H-^1^H COSY, HMQC, and HMBC spectra (see Tables S1–S7 for **1**–**7** and see also their spectra, all in the Supplementary Information).

Interpretation of the ^1^H–^1^H COSY and HMQC data established structural parts related to the proton spin systems including OH and NH in **1**–**7**. The structure moieties of 3-hydroxydodecaonic acid (HDA, C21→C32), asparagine (Asn, C15→N20), and glutamine (Gln, C8→N14) in **1**–**7** were deduced by the HMBCs detected on H_2_-22/C-21, H-23/C-21, HO-23/C-22, HO-23/C-23, and HO-23/C-24 in HAD, on H-16/C-15, H-16/C-18, H_2_-17/C-15, H_2_-17/C-18, H_2_-19/C-17, H_2_-19/C-18, and H-20/C-15 in Asn, and on H-9/C-8, H_2_-10/C-8, H_2_-10/C-12, H_2_-11/C-12, H_2_-13/C-11, H_2_-13/C-12, and H-14/C-8 in Gln moieties. In addition, the HMBCs on H_2_-1'/C-2 in **1**–**2** and H_3_-1'/C-2 in **3**–**4** demonstrated the presence of an isopentyl amino group (N7→C2→C6) with an ethoxy group at C-2 in **1**–**2** and a methoxy group at C-2 in **3**–**4**. The HMBCs on H_2_-1′/C-1, H_2_-1′/C-2, H-2/C-1, and H_2_-3/C-1 of **5** and **6** established the leucine (Leu) amide residues in **5** and **6**. The connectivity of the HAD, Asn and Gln in **1**–**7** were determined by the HMBCs of H-16 and H-20 in Asn with C-21 in HDA and of H-9 and H-14 in Gln with C-15 in Asn. The isopentyl amino groups in **1**–**4** and Leu amide residues in **5** and **6** were further connected to C-8 in Gln by the HMBCs of H-2 and H-7 with C-8 in **1**–**4** and **5**–**6**. The terminal Gln in **7** was evidenced by the HMBCs on H_2_-7/C-8 and H_2_-7/C-9.

**Table 1:** ^1^H NMR data of **1**–**9** in DMSO-*d*_6_ (δ_H_, *J* in Hz) ^a^.

Proton	1 ^b^	2 ^b^	3 ^c^	4 ^c^	5 ^b^	6 ^b^	7 ^c^	8 ^c^	9 ^c^
1	—	—	—	—	—	—	—	9.35 s	3.30 ddd (10.6, 5.6, 4.8)
	—	—	—	—	—	—	—	—	3.23 ddd (10.6, 6.0, 4.8)
2	5.07 dt (9.2, 6.9)	5.06 dt (9.1, 6.9)	4.97 dt (9.1, 6.9)	4.96 dt (9.4, 7.2)	4.07–4.02 m	4.15–4.07 m	—	4.08–4.00 m	3.84–3.72 (overlapped)
3	1.48 dt (13.8, 6.9)	1.45 dt (13.8, 6.9)	1.49 dt (13.8, 6.9)	1.49–1.38 (2H) m	1.59–1.50 m	1.59–1.49 m	—	1.56–1.44 (2H) m	1.40–1.25 (overlapped)
	1.40–1.33 m	1.40 dt (13.8, 6.9)	1.42–1.30 m	—	1.48–1.42 m	1.49–1.40 m	—	—	—
4	1.65–1.57 m	1.60–1.52 m	1.65–1.54 m	1.61–1.49 m	1.59–1.50 m	1.59–1.49 m	—	1.68–1.58 m	1.63–1.52 m
5	0.83 d (6.8)	0.81 d (6.6)	0.83 d (6.7)	0.81 d (6.6)	0.80 d (6.6)	0.79 d (6.2)	—	0.84 d (6.4)	0.82 d (6.4)
6	0.86 d (6.7)	0.84 d (6.8)	0.85 d (6.7)	0.84 d (6.6)	0.87 d (6.6)	0.84 d (5.6)	—	0.89 d (6.8)	0.86 d (6.4)
7	8.02 d (9.2)	8.04 d (9.1)	8.03 d (9.1)	8.04 d (9.4)	7.86 d (8.4)	7.87 d (8.4)	7.34 br s	8.27 d (7.2)	7.43 d (7.6)
							7.08 br s		
9	4.13 td (7.8, 4.6)	4.13–4.05 m	4.18–4.08 m	4.13–4.05 m	4.13–4.09 m	4.15–4.07 m	4.06 td (8.0, 4.6)	4.21–4.16 m	4.15–4.05 (overlapped)
10	1.99–1.89 m	1.97–1.90 m	2.02–1.88 m	2.00–1.89 m	1.95–1.88 m	2.00–1.92 m	1.95 dtd (13.2, 7.5, 4.6)	2.02–1.90 m	1.97–1.85 m
	1.78–1.68 m	1.78–1.70 m	1.80–1.68 m	1.81–1.69 m	1.78–1.72 m	1.77–1.67 m	1.70 ddt (13.2, 8.0, 7.5)	1.82–1.68 m	1.78–1.65 m
11	2.12–2.04 m	2.13–2.03 m	2.14–2.03 m	2.13–2.05 m	2.21–2.16 m	2.08–2.00 m	2.06 t (7.5)	2.14–2.04 m	2.11–2.02 m
13	7.21 br s	7.20 br s	7.25 br s	7.24 br s	7.22 br s	7.18 br s	7.21 br s	7.21 br s	7.20 br s
	6.75 br s	6.74 br s	6.79 br s	6.78 br s	6.76 br s	6.71 br s	6.75 br s	6.76 br s	6.74 br s
14	7.94 d (7.8)	8.06 d (7.6)	8.05 d (8.0)	8.12 d (7.6)	8.10 d (7.2)	8.09 d (8.4)	7.98 d (8.0)	8.10 d (7.6)	8.00 d (8.0)
16	4.49 q (7.0)	4.50 q (7.0)	4.49 q (6.9)	4.50 q (7.0)	4.48 q (7.2)	4.45 q (6.9)	4.48 q (7.0)	4.50 q (7.2)	4.48 br q (6.9)
17	2.54 dd (15.6, 7.0)	2.55 dd (15.6, 7.0)	2.54 dd (15.5, 6.9)	2.55 dd (15.6, 7.0)	2.58–2.42 m	2.54 dd (15.6, 6.9)	2.57 dd (15.5, 7.0)	2.55 dd (15.5, 7.2)	2.55 dd (15.6, 6.9)
	2.43 dd (15.6, 7.0)	2.43 dd (15.6, 7.0)	2.43 dd (15.5, 6.9)	2.42 dd (15.6, 7.0)	2.58–2.42 m	2.43 dd (15.6, 6.9)	2.43 dd (15.5, 7.0)	2.44 dd (15.5, 7.2)	2.43 dd (15.6, 6.9)
19	7.37 br s	7.40 br s	7.41 br s	7.43 br s	7.46 br s	7.41 br s	7.42 br s	7.42 br s	7.42 br s
	6.90 br s	6.94 br s	6.93 br s	7.24 br s	6.95 br s	6.91 br s	6.96 br s	6.93 br s	6.94 br s
20	8.08 d (7.0)	8.05 d (7.0)	8.12 d (6.9)	8.08 d (7.0)	8.26 d (7.2)	8.15 d (7.2)	8.09 d (7.0)	8.09 d (7.2)	8.10 d (6.9)
22	2.26–2.16 m	2.25–2.16 m	2.27-2.16 m	2.25–2.16 m	2.24–2.16 m	2.25–2.15 m	2.25–2.14 m	2.24–2.16 m	2.25–2.17 m
23	3.82–3.75 m	3.80–3.75 m	3.84–3.73 m	3.82–3.72 m	3.80–3.74 m	3.81–3.74 m	3.82–3.73 m	3.82–3.72 m	3.84–3.72 (overlapped)
24	1.40–1.33 m	1.39–1.30 m	1.42–1.30 m	1.38–1.30 m	1.40–1.18 m	1.40–1.18 m	1.40–1.20 m	1.40–1.20 m	1.40–1.20 m
25	1.40–1.33 m	1.39–1.30 m	1.42–1.30 m	1.38–1.30 m	1.40–1.18 m	1.40–1.18 m	1.40–1.20 m	1.40–1.20 m	1.40–1.20 m
	1.30–1.18 m	1.30–1.18 m	1.30–1.18 m	1.30–1.18 m	1.40–1.18 m	1.40–1.18 m	1.40–1.20 m	1.40–1.20 m	1.40–1.20 m
26	1.30–1.18 m	1.30–1.18 m	1.30–1.18 m	1.30–1.18 m	1.40–1.18 m	1.40–1.18 m	1.40–1.20 m	1.40–1.20 m	1.40–1.20 m
27	1.30–1.18 m	1.30–1.18 m	1.30–1.18 m	1.30–1.18 m	1.40–1.18 m	1.40–1.18 m	1.40–1.20 m	1.40–1.20 m	1.40–1.20 m
28	1.30–1.18 m	1.30–1.18 m	1.30–1.18 m	1.30–1.18 m	1.40–1.18 m	1.40–1.18 m	1.40–1.20 m	1.40–1.20 m	1.40–1.20 m
29	1.30–1.18 m	1.30–1.18 m	1.30–1.18 m	1.30–1.18 m	1.40–1.18 m	1.40–1.18 m	1.40–1.20 m	1.40–1.20 m	1.40–1.20 m
30	1.30–1.18 m	1.30–1.18 m	1.30–1.18 m	1.30–1.18 m	1.40–1.18 m	1.40–1.18 m	1.40–1.20 m	1.40–1.20 m	1.40–1.20 m
31	1.30–1.18 m	1.30–1.18 m	1.30–1.18 m	1.30–1.18 m	1.40–1.18 m	1.40–1.18 m	1.40–1.20 m	1.40–1.20 m	1.40–1.20 m
32	0.85 t (7.0)	0.86 t (7.2)	0.85 t (7.2)	0.86 t (6.8)	0.85 t (6.9)	0.85 t (6.9)	0.86 t (6.8)	0.85 t (6.8)	0.86 t (6.8)
1'	3.44 dq (14.1, 7.0)	3.46 dq (14.1, 7.0)	3.11 (3H) s	3.12 (3H) s	6.97 br s	7.17 br s	—	—	—
	3.27 dq (14.1, 7.0)	3.28 dq (14.1, 7.0)	—	—	6.95 br s	6.97 br s	—	—	—
2'	1.05 t (7.0)	1.05 t (7.0)	—	—	—	—	—	—	—
1–O H	—	—	—	—	—	—	—	—	4.54 br t (4.8)
23–O H	4.64 d (4.8)	4.62 d (4.8)	4.68 d (4.8)	4.65 d (5.1)	4.61 d (5.4)	4.66 br s	4.65 d (5.1)	4.63 d (5.2)	4.64 br d (4.8)

^a^ Chemical shifts were recorded in δ values using the solvent DMSO-*d*_6_ signal δ_H_ 2.50 as reference; Signal assignments were based on the results of DEPT, ^1^H–^1^H COSY, HMQC, and HMBC experiments; ^b^ Recorded at 600 MHz ^1^H NMR; ^c^ Recorded at 400 MHz ^1^H NMR.

**Table 2:** ^13^C NMR data of **1**–**9** in DMSO-*d*_6_ (δ_C_, multiplicity) ^a^.

Carbon	1 ^b^	2 ^b^	3 ^c^	4 ^c^	5 ^b^	6 ^b^	7 ^c^	8 ^c^	9 ^c^
1	—	—	—	—	174.2 s	174.3 s	—	201.6 s	63.7 t
2	77.2 d	77.3 d	78.9 d	78.9 d	49.9 d	48.7 d	—	56.6 d	48.8 d
3	43.3 t	43.1 t	43.0 t	42.8 t	40.0 t	40.2 t	—	36.3 t	40.2 t
4	24.0 d	23.9 d	24.1 d	24.0 d	24.2 d	24.1 d	—	23.9 d	24.1 d
5	22.3 q	22.2 q	22.4 q	22.2 q	21.2 q	21.8 q	—	21.3 q	22.1 q
6	22.4 q	22.5 q	22.5 q	22.7 q	23.0 q	23.4 q	—	23.1 q	23.4 q
8	171.5 s	171.6 s	171.7 s	171.8 s	170.9 s	170.5 s	173.3 s	171.9 s	170.5 s
9	52.6 d	52.8 d	52.8 d	53.9 d	53.2 d	52.6 d	52.3 d	52.4 d	52.7 d
10	27.6 t	27.5 t	27.6 t	27.5 t	27.1 t	27.7 t	27.5 t	27.5 t	27.8 t
11	31.4 t	31.4 t	31.5 t	31.5 t	31.4 t	31.5 t	31.5 t	31.4 t	31.5 t
12	173.7 s	173.7 s	173.9 s	173.8 s	173.8 s	173.9 s	173.8 s	173.8 s	173.9 s
15	171.0 s	171.1 s	171.3 s	171.2 s	170.9 s	170.9 s	170.8 s	171.1 s	170.9 s
16	49.9 d	49.7 d	50.0 d	49.8 d	49.9 d	49.8 d	49.8 d	49.8 d	49.9 d
17	36.9 t	37.0 t	36.9 t	36.9 t	36.9 t	36.8 t	36.9 t	37.0 t	37.0 t
18	171.6 s	171.7 s	171.8 s	171.9 s	171.8 s	171.8 s	171.9 s	171.8 s	171.8 s
21	171.2 s	171.1 s	171.2 s	171.1 s	171.2 s	171.2 s	171.1 s	171.2 s	171.2 s
22	43.4 t	43.4 t	43.5 t	43.5 t	43.4 t	43.4 t	43.5 t	43.5 t	43.5 t
23	67.4 d	67.4 d	67.5 d	67.5 d	67.4 d	67.4 d	67.5 d	67.5 d	67.4 d
24	36.8 t	36.8 t	37.0 t	37.1 t	37.0 t	36.9 t	36.9 t	36.9 t	36.9 t
25	25.1 t	25.1 t	25.2 t	25.2 t	25.0 t	25.1 t	25.1 t	25.1 t	25.1 t
26	29.1t	29.1t	29.2 t	29.2 t	29.1 t	29.1 t	28.7 t	29.1 t	29.1 t
27	29.1t	29.1t	29.1 t	29.2 t	29.1 t	29.0 t	29.0 t	29.1 t	29.1 t
28	29.0 t	29.0 t	29.1 t	29.1 t	28.9 t	28.9 t	29.1 t	29.0 t	29.0 t
29	28.7 t	28.7 t	28.8 t	28.8 t	28.7 t	28.7 t	29.1 t	28.7 t	28.7 t
30	31.3 t	31.3 t	31.4 t	31.4 t	31.2 t	31.3 t	31.3 t	31.3 t	31.3 t
31	22.0 t	22.0 t	22.2 t	22.2 t	22.0 t	22.1 t	22.1 t	22.1 t	21.8 t
32	13.9 q	13.9 q	14.0 q	14.0 q	13.9 q	13.9 q	14.0 q	14.0 q	14.0 q
1′	61.8 t	61.8 t	54.3 q	54.4 q	—	—	—	—	—
2′	15.0 q	15.1 q	—	—	—	—	—	—	—

^a^ Chemical shifts were recorded in δ values using the solvent DMSO-*d*_6_ signal δ_C_ 39.52 as reference. Multiplicities of the carbon signals were determined by the DEPT method and are shown as s (singlet), d (doublet), t (triplet), and q (quartet), respectively. Signals were assigned on the basis of DEPT, ^1^H–^1^H COSY, HMQC, and HMBC experiments; ^b^ Recorded at 150 MHz ^13^C NMR; ^c^ Recorded at 100 MHz ^13^C NMR.

The absolute configurations of Asn and Gln in **1**–**7** and Leu amide further in **5**–**6** were determined by Marfey’s method [22] using d- and l-Leu amides, d- and l-Asn, and d- and l-Gln as standards. We hydrolyzed **1**–**7** with 6 N HCl at 110 °C for 24 hours to produce hydrolysates containing aspartic and glutamic acids in **1**–**7** and leucine further in **5**–**6**. Each standard was hydrolyzed also in the same conditions. The hydrolysates of **1**–**7** and the standards were combined with Marfey’s reagent to derivatize the amino acids. HPLC analyses of the Marfey’s derivatives from **1**–**7**, using the derivatives of the l- and d-standards as references, established the l configurations of Asn (16*S*) and Gln (9*S*) in **1**–**7**, the l configuration of Leu amide (2*S*) in 5, and the d configuration of the Leu amide (2*R*) in **6**. The 23*R* absolute configurations of **1**–**7** were determined as follows. The 23*R* absolute configurations of the known **8** and **9** were determined by the chemical and Mosher’s methods in the literature [15,16]. Coproduction of **1**–**9** by the mutant AD-2-1 revealed the same absolute configuration 23*R* in **1**–**7** from a biogenetic consideration. Coincidently, hydrolysis of **1**–**9** mixtures in 6 N HCl at 110 °C for 1 h, as reported [23], produced 3*R*-hydroxydodecanoic acid (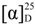 −16.0 (*c* 0.05, CHCl_3_); lit. 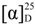 −17.8 (*c* 1.2, CHCl_3_) [24]) and established the 23*R* configuration in **1**–**7**. Therefore, absolute configurations have been defined for all chiral carbons in **1**–**7** except for the C-2 carbons in **1**–**4**. Thus, **1**/**2** and **3**/**4** were each pairs of epimers at C-2.

Although several structurally related lipopeptides have been known to occur in Nature [15,16,23], **1**–**7** are the first examples of new lipopeptides generated by mutagenic activation of silenced metabolic pathways in fungi. Among the lipopeptides reported, fellutamides A (10-hydroxylated B) and B (**8** in Figure 1) were isolated in 1991 from *Penicillium fellutanum* and given their names by the mixed characters from the species name *fellutanum* and the group name of compounds “amide” [15]. Both compounds carry 3-hydroxydodecaonic acid (HDA) side chains at N-20 and leucinal residues at N-7. Later in 2010 [16], the lipopeptide **9** shown in Figure 1 was initially isolated as a new compound from *Penicillium vercicolor* and named fellutamide C in view of the structural similarity to fellutamides A and B (**8**). Fellutamide C (**9**) carries an l-leucinol residue, instead of the leucinal residue in **8**. Later in 2011, a new lipopeptide from an undescribed fungal species of *Metulocladosporiella* was reported by Xu *et al.* giving the same name “fellutamide C” [23]. The “fellutamide C” differs from **8** in carrying a 3-hydroxytetradecanoic acid (HTDA) side chain at N-20. However, the structure of this compound was apparently different from the fellutamide C [16] (**9**) and this has thus confused the names of the same class of compounds. Xu *et al*. [23] reported also another new lipopeptide in this class of compounds, named fellutamide D, which differed from their “fellutamide C” in the valinal residue at N-7, instead of the leucinal residue in “fellutamide C”. We therefore propose here that the “fellutamide C” should be renamed, and fellutamide E was suggested for the name of this compound. Incidentally, we named the new lipopeptides **1**–**7** as penicimutalides A–G (**1**–**7**), which differ from the fellutamides in the leucine-derived moieties at N-7, and this would avoid similar confusion.

### 2.3. HPLC-PDAD-UV/HPLC-ESI-MS Analyses of G59 and AD-2-1 Extracts for Detecting **1–14**

The EtOAc extracts of the mutant AD-2-1 and the strain G59 were subjected to HPLC-PDAD-UV and HPLC-ESI-MS analyses, using **1**–**14** as reference standards, to confirm the activated production of **1**–**14** in the mutant AD-2-1 by the DES mutagenesis of original G59 strain. The reference standards **1**–**6** were used as mixed pairs of **1**/**2**, **3**/**4**, and **5**/**6**, respectively, because the peaks of two compounds in these pairs had been known to appear at the same retention times in the given HPLC condition using an achiral, reversed phase analytical column by pilot test of HPLC analysis.

**Figure 2:**
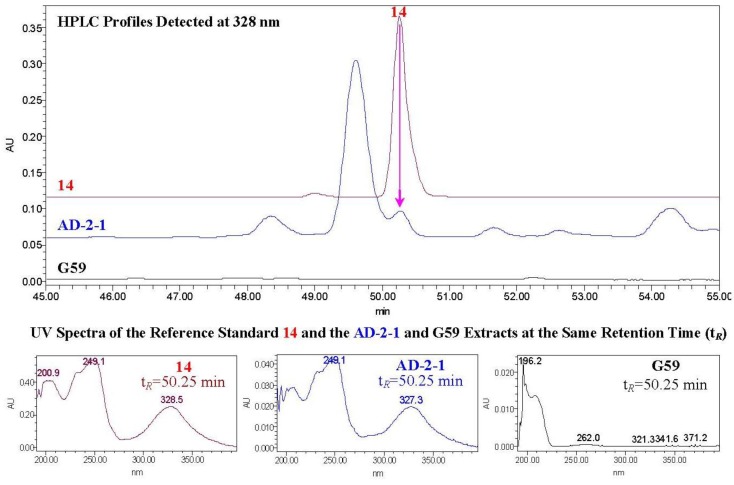
HPLC-PDAD-UV analysis of AD-2-1 and G59 EtOAc extracts for detecting **14**.

In the HPLC-PDAD-UV analysis, **1**–**9** were eluted as peaks with retention times of 56.12 min for **1**/**2**, 54.57 min for **3**/**4**, 50.62 min for **5**/**6**, 46.52 min for **7**, 52.77 min for **8**, and 52.57 min for **9**, respectively. Because **1**–**9** showed only the end UV absorptions, they were hardly identified by the HPLC-PDAD-UV analysis both in the mutant AD-2-1 and the control G59 extracts due to the lack of the typical UV absorption curves and the baseline drift (see Figure S1-A1,A2 in the Supplementary Information). On the other hand, **10**–**14** were eluted as peaks with retention times of 63.90 min for **10**, 59.50 min for **11**, 63.00 min for **12**, 52.12 min for **13**, and 50.25 min for **14**, respectively. They were all detected in the mutant AD-2-1extract but not in the parent G59 extract. The data for the detection of **14** is shown as a typical example in Figure 2. The detection of **10**–**14** was achieved by both the retention times and the UV absorption curves (see Figure 2 and see also Figure S1-B1,B2,C1,C2 in the Supplementary Information).

In the HPLC-ESI-MS analysis, retention times of the ion peaks of **1**–**14** were slightly shortened (see Experimental Section) than those in HPLC-PDAD-UV analysis because of the shortened flow length from the outlet of the HPLC column to the inlet of MS in the HPLC-ESI-MS. By selective ion ([M + Na]^+^ for **1**/**2** and **14**; [M − H]^−^ for **5**/**6**, **7**, **8**, and **10**–**13**; [M + Cl]^−^ for **3**/**4** and **9**) monitoring with both extracted ion chromatograms and related MS spectra, **1**–**14** were all detected in the mutant AD-2-1 extract, but none of these metabolites were detected in the G59 extract (see Figure S2A–K in the Supplementary Information).

These analyses indicated that the production of **1**–**14** in mutant AD-2-1 was caused by the activation of silent metabolic pathways in the original G59 strain by the DES mutagenesis. Although the silent pathways remained undefined, the structural features of **1**–**9** and **10**–**14** indicated they may originate from the nonribosomal peptide synthetase (NRPS) systems and the polyketide synthase (PKS) systems, respectively [25,26]. Various kinds of lipopeptides are known occurring in Nature. However, **1**–**7** are new lipopeptides and several analogues including **8** and **9** were all discovered from fungal metabolites [15,16,23]. Also, a lot of anthraquinone/xanthone-derived polyketides have been isolated from fungal metabolites [25,26]. However, none of them were obtained by mutagenic activation of their production, and **1**–**9** and **10**–**14** provide the first examples of the related lipopeptides and polyketides produced by mutagenic activation of the silent metabolic pathways in fungi. Thus, additional investigation into the activated metabolic pathways and their regulatory mechanisms are warranted.

### 2.4. Inhibitory Effects of **1–14** on Several Human Cancer Cell lines

Antitumor activities of **1***–***14** were assayed by the MTT method using human cancer K562, HL-60, HeLa, BGC-823, and MCF-7 cells. Seven new lipopeptides **1***–***7** and the known one **9** weakly inhibited these cells to varying extents with inhibition rate (IR%) values shown in Table 3, and the five known polyketides **10***–***14** showed also similarly weak inhibitory effects on the K562 cells with the IR% values of 11.6% (**10**), 51.9% (**11**), 37.9% (**12**), 25.5% (**13**) at 100 µg/mL, and 37.5% (**14**) at 50 µg/mL. In contrast, the known *C*-terminal aldehyde lipopeptide fellutamide B (**8**) showed stronger activities than **1***–***7** and **9***–***14** in inhibiting the tested cell lines with half-inhibitory concentration (IC_50_) values of 29.1 µg/mL (52.5 µM) for K562, 59.9 µg/mL (107.9 µM) for HL-60, 59.5 µg/mL (107.2 µM) for HeLa, 77.9 µg/mL (140.4 µM) for BGC-823, and 43.3 µg/mL (68.4 µM) for MCF-7 cells. The positive control 5-flurouracel (5-FU) inhibited these cell lines with the IR% values of 48.5% (K562), 38.2% (HL-60), 37.4% (HeLa), 47.8% (BGC-823), and 47.4% (MCF-7) at 100 µg/mL. The above data were taken with four new compounds that we have just recently reported [12], using 5-FU as the positive control at the same time and same conditions, so that the same data for 5-FU [12] were also given as references for **1**–**14**.

**Table 3:** IR% values of **1**–**7** and **9** on human cancer cell lines by MTT assay at 100 µg/mL.

Compound	K562	HL-60	HeLa	BGC-823	MCF-7
**1**	37.6%	14.1%	12.8%	19.9%	33.0%
**2**	34.4%	28.0%	27.6%	28.1%	34.5%
**3**	40.7%	19.8%	19.1%	13.5%	27.0%
**4**	40.0%	29.6%	29.0%	27.9%	38.0%
**5**	17.0%	42.6%	42.0%	43.9%	12.5%
**6**	10.1%	38.8%	37.9%	49.0%	21.8%
**7**	16.1%	12.0%	11.5%	10.3%	16.5%
**9**	48.3%	33.4%	33.0%	33.3%	35.5%

Fellutamide A and B (**8**) were originally identified for their cytotoxic properties on murine leukemia P388 and L1210, and human epidermoid carcinoma KB cells with IC_50_ values 0.1–0.8 µg/mL [15]. Later they were found to stimulate nerve growth factor (NGF) synthesis and secretion [27,28]. The similar fellutamides carrying *C*-terminal aldehyde group, fellutamide D and “fellutamide C” that we suggest to be renamed as fellutamide E, were also cytotoxic against human prostate carcinoma PC-3 cells (IC_50_: 160 and 440 nM, respectively) and further showed antifungal activities on several *Candida* species with MIC values ranging from 2 to 32 µg/mL [23]. Now, the *C*-terminal aldehyde fellutamides are known to be members of peptide aldehyde proteasome inhibitors [23,29,30], and their inhibition of the human proteasome, probably by formation of hemiacetal covalent adduct at the active site of the proteasome [29,30], induced the NGF synthesis that may result in the neurotrophic effects [27,28,29,30]. Meanwhile, the inhibition of human proteasome by the *C*-terminal aldehyde fellutamides is also likely to be the reason for their cytotoxicity [23]. Recently, fellutamide B (**8**) was known also to potently inhibit the *Mycobacterium tuberculosis* proteasome with kinetic mechanisms that differ from its inhibition of the human proteasome [31]. Fellutamide C (**9**), the only fellutamide without *C*-terminal aldehyde group, was recorded to exhibit the cytotoxic activity on the human lung cancer A549, human ovarian cancer SK-OV-3, human skin cancer SK-MEL-2, human CNS cancer XF498, and human colon cancer HCT-15 cells with half-effective dosage (ED_50_) values ranging from 3.1 to 33.1 µM [16]. Our bioassay results for **8** and **9** on the five human cancer cell lines in the present study confirmed their cytotoxic properties previously recorded on the other cell lines [15,16], but their inhibitory effects on the present cell lines are much lower than recorded. Furthermore, the seven new and one known lipopeptides all without the *C*-terminal aldehyde group, penicimutalides A–G (**1**–**7**) and fellutamide C (**9**), showed also much lower cytotoxicity than the *C*-terminal aldehyde lipopeptide, fellutamide B (**8**), on the five tested cell lines in the present bioassays. These data have revealed that the *C*-terminal aldehyde is an important functional group for their cytotoxic effects on these cell lines, and suggested also that the inhibition of the human proteasome by the aldehyde group in *C*-terminal fellutamides [23,29] might have contributed to the cytotoxicity of these fellutamides [15,23,29].

In summary, we have reported 14 metabolites **1**–**14** in the culture extract of an antitumor mutant AD-2-1 that was obtained by the DES mutagenesis of originally inactive, parent G59 fungal strain. By tracing newly produced metabolites in the mutant AD-2-1 extract under the guidance of MTT assay and TLC analysis using the parent G59 extract as control, seven new lipopeptides **1**–**7** were isolated together with two known lipopeptides 8 and 9 and the five known polyketides **10**–**14**. Structures of the new compounds including their absolute configurations were determined by spectroscopic and chemical evidences, and named penicimutalides A–G (**1**–**7**). All **1**–**14** inhibited several human cancer cell lines to varying extents. All the bioassays and HPLC-PDAD-UV and HPLC-ESI-MS analyses demonstrated that the production of **1**–**14** in the mutant AD-2-1 was caused by the activation of the silent metabolic pathways in the original fungal G59 strain by the DES mutagenesis. Present results provided additional examples for the effectiveness of the chemical mutagenesis strategy using an old chemical mutagen DES to discover new compounds by activating silent metabolites in fungal isolates [12].

## 3. Experimental Section

### 3.1. General Experimental

Melting points were measured on a Beijing Tiandiyu X-4 exact micro melting point apparatus and the temperatures were not corrected. Optical rotations were measured on an Optical Activity Limited polAAr 3005 spectropolarimeter. ESIMS was recorded on an Applied Biosystems API 3000 LC-MS spectrometer. HRESIMS was measured on an Agilent 6520 Q-TOF LC-MS spectrometer. IR spectra were taken on a Bruker Hyperion ATR-objective spectrophotometer or Bruker Tensor-27 infrared spectrophotometer. All 1D and 2D NMR spectra were obtained on a JEOL JNM-GX 400 (400 MHz ^1^H and 100 MHz ^13^C NMR) or Bruker-600 (600 MHz ^1^H and 150 MHz ^13^C NMR) NMR spectrometer. The chemical shifts of ^1^H and ^13^C NMR signals were recorded in δ values using solvent signals (CDCl_3_: δ_H_ 7.26, δ_C_ 77.1; DMSO-*d*_6_: δ_H_ 2.50, δ_C_ 39.5; and acetone-*d*_6_: δ_H_ 2.05, δ_C_ 206.26) as references, respectively.

Precoated silica gel GF_254_ plates (10 cm × 20 cm, 0.25 mm thickness for analytical TLC and 20 cm × 20 cm, 0.5 mm thickness for preparative TLC; Yantai Chemical Industrial Institute, Yantai, China) and polyamide thin layers (10 cm × 20 cm, Taizhou Luqiao Sijia Biochemical plastic factory, Taizhou, China) were used in TLC and spots were detected under sunlight and UV light (254 and 365 nm) or by using Vaughan’s reagent (24 g of ammonium molybdate tetrahydrate (NH_4_)_6_Mo_7_O_24_·4H_2_O) and 1 g of ceric sulfate Ce(SO_4_)_2_ dissolved in 500 mL of 10% H_2_SO_4_), 5% FeCl_3_ reagent (5 g of FeCl_3_ dissolved in 100 mL of 95% aqueous EtOH) or 10% sulfuric acid reagent. For lipopeptides, TLC spots or bands were also detected by spraying distilled water onto the plates. Silica gel H (100–200 mesh, Yantai Chemical Industrial Institute, Yantai, China), YMC*GEL^®^ ODS-A-HG (12 nm S-50 µm, YMC Co., Ltd., Kyoto, Japan), Sephadex™ LH-20 (GE Healthcare, Uppsala, Sweden), and Polyamide (100–200 mesh, Taizhou Luqiao Sijia Biochemical plastic factory, Taizhou, China) were used for column chromatography. Both analytical and semi-preparative HPLC were performed on Waters HPLC systems equipped with Waters 600 controller, Waters 600 pump, Waters 2414 refractive index detector, Waters 2996 (for analytical HPLC) or 2998 (for preparative HPLC) photodiode array detector, and Waters Empower™ software. Venusil MP C_18_ (5 µm, 100 Å, 4.6 mm × 250 mm; Agela Technologies, Tianjin, China) and reversed-phase chiral Click β-CD (4.6 mm × 150 mm; Dalian Institute of Chemical Physics, Dalian, China) columns were used in analytical HPLC, and reversed-phase chiral Click β-CD column (8 mm × 150 mm; Dalian Institute of Chemical Physics, Dalian, China) was used in semi-preparative HPLC.

Marfey’s reagent, the 1-fluoro-2,4-dinitrophenyl-5-l-alanineamide (FDAA), was the Sigma product, which was provided by Yongsheng Che (Beijing Institute of Pharmacology and Toxicology, Beijing, China). The standard materials used in Marfey analysis were purchased from ShangHai HanHong Chemical CO., Ltd. (Shanghai, China) Standard (abbr.), lot No: l-asparagine (l-Asn), BH-00303-100101; d-asparagine (d-Asn), BH-00301-100801; l-glutamine (l-Gln), BH-00802-100101; d-glutamine (d-Gln) BH-00801-100101; l-leucinol (l-Leo), BH-01338-100501; d-leucinol (d-Leo) BH-01337-100501; l-leucine (l-Leu), BH-01302-110501; d-leucine (d-Leu), BH-01301-110101; l-aspartic acid (l-Asp) BH-00403-110501; d-aspartic acid (d-Asp), BH-00401-110502; l-glutamic acid (l-Glu), BH-00702-110501; d-glutamic acid (d-Glu), BH-00701-110501.

ZHWY-2102 rotary shakers (Shanghai ZhiCheng Analyzing Instrument Manufactory Co., Ltd., Shanghai, China) were used for the fermentation of fungal strains. The VERSAmax-BN03152 micro plate reader (Molecular Device) was used in bioassays to read the optical density (OD) and the AE31 EF-INV inverted microscope (Motic China Group Co., Ltd., Xiamen, Fujian, China) was used for morphological examination of tumor cells.

Human chronic myelogenous leukemia K562 cell line was provided by Prof. Dr. Lili Wang (Beijing Institute of Pharmacology and Toxicology, Beijing, China). Human acute promyelocytic leukemia HL-60, human cervical cancer HeLa, Human gastric adenocarcinoma BGC-823, and human breast cancer MCF-7 cell lines were provided by Wenxia Zhou (Beijing Institute of Pharmacology and Toxicology, Beijing, China). Fetal bovine serum was purchased from Tianjin Hao Yang Biological manufacture Co., Ltd. (Tianjin, China). The RPMI-1640 medium was purchased from Gibco (lot No. 1403238) and MTT from Amresco (lot No. 0793). Streptomycin (lot No. 071104) and penicillin (lot No. X1103302) were both purchased from North China Pharmaceutical Group Corporation, China. The 5-fluorouracil (5-FU, lot No.5402) was purchased from Aladdin Chemistry Co., Ltd. (Shanghai, China).

### 3.2. MTT Assay

EtOAc extracts and fractions were dissolved in MeOH at 10 mg/mL and the MeOH solutions were used in MTT assays. Pure compounds and 5-FU were dissolved in MeOH and DMSO to prepare 10.0 mg/mL stock solutions, respectively, and serial dilutions were made for MTT assay. 5-FU was used as positive control, and MeOH and DMSO were used as blank control, respectively.

Exponentially growing K562, HL-60, HeLa, BGC-823 and MCF-7 cells were suspended in fresh RPMI-1640 medium containing 10% fetal bovine serum and 100 µg/mL penicillin and streptomycin at the cell density of 5 × 10^4^ cells/mL and seeded into 96-well plates each 200 µL/well. The suspension cells, K562 and HL-60, were incubated at 37 °C for 2 h, whereas the adherent cells, HeLa, BGC-823 and MCF-7, were incubated at 37 °C for 12 h. Then, 2 µL of MeOH or DMSO for control and the test sample solutions was added to each well, and the cells were cultured at 37 °C for 24 h. After morphological examination of the cells under an inverted microscope, MTT (20 µL; 5 mg/mL in PBS) was added into each well, incubated at 37 °C for 4 h, and centrifuged at 2000 rpm, 4 °C for 20 min. After removal of the supernatant by aspirating, 150 µL DMSO was added into each well, and shaken for 5 min to dissolve formazan crystals. The OD value at 570 nm was read for each well using the VERSAmax-BN03152 plate reader. Each three wells were set for control and test groups, respectively, and the inhibition rate (IR%) was calculated using OD mean values according to the formula, IR% = (OD_control_ − OD_sample_)/OD_control_ × 100%. The IC_50_ value for a sample was obtained from the IR% values of the sample at different concentrations.

### 3.3. Fermentation and EtOAc Extract Preparation

#### 3.3.1. Initial Fungal Strain and its Mutant the **1**–**14** Producing Strain

The initial fungal strain, *Penicillium purpurogenum* G59 that was used as control strain in the present study, was isolated from a soil sample collected at the tideland of Bohai Bay around Lüjühe in Tanggu district of Tianjin, China, in September 2004 [14]. *P. purpurogenum* G59 was identified by Professor Dr. Liangdong Guo, Institute of Microbiology, Chinese Academy of Sciences, China. This strain has been deposited at the China General Microbiological Culture Collection Center under the accession number CGMCC No.3560. The strain *P. purpurogenum* G59 did not produce any secondary metabolites with antitumor activities in repeated MTT assays using K562 cells at high concentrations of 100 and 1000 µg/mL [10,11,12,13,14].

The producing strain used for **1**–**14** production in present study is a bioactive mutant AD-2-1 that was obtained by DES mutagenesis of the strain G59 [12]. Fresh G59 spores in 50% (v/v) DMSO were treated with 1% (v/v) DES at 4 °C for 1 day, and single colony isolation on the treated spores followed by bioassays afforded the mutant. EtOAc extracts of the mutant cultures inhibited K562 cells (an IR% of 49.8% at 100 µg/mL), whereas the EtOAc extract of strain G59 by the fermentation at the same time and same conditions did not inhibit the K562 cells (an IR% of 5.6% at 100 µg/mL) [12]. The mutant AD-2-1 has been deposited at the China General Microbiological Culture Collection Center under the accession number CGMCC No.3561.

#### 3.3.2. Preparation of Spore Suspensions

The mutant AD-2-1 was inoculated onto potato dextrose agar (PDA) plates from a PDA slant stock stored at 4 °C and incubated at 28 °C for 4 days. Fresh spores formed on the PDA plates were harvested and suspended in 80 mL of sterilized, distilled water with several glass beads in a 100 mL cone-shaped flask and scattered well by shaking enough to prepare a crude spore suspension. A 100 µL portion of this crude spore suspension was added into a well of 96-well plates, diluted with water with its OD at 600 nm measured using a VERSAmax-BN03152 plate reader, and the dilution ratio was recorded when the OD value reached 0.35. Then, the remaining whole crude spore suspension was diluted with sterilized, distilled water in the same proportion to obtain a mutant AD-2-1 spore suspension. This mutant spore suspension was used for the producing fermentation in the following experiments.

Similarly, the G59 spore suspension was also prepared in the same manner as mentioned above using fresh spores formed by cultivation of the G59 strain at 28 °C for 3 days on PDA plates. This G59 spore suspension was used as control strain in the following experiments.

#### 3.3.3. Fermentation and Extraction

Aliquot (200 µL) of the mutant AD-2-1 spore suspension was inoculated into 100 cone-shaped 500 mL flasks containing 200 mL of the liquid medium (glucose 2%, maltose 1%, mannitol 2%, glutamic acid 1%, peptone 0.5% and yeast extract 0.3% in distilled water, adjusted to pH 6.0 prior to sterilization) and fermented at 28 °C for 12 days on rotary shakers at 200 rpm to obtain approximate 20 L of fermentation broth. The whole broth (20 L) was filtered to separate into a filtrate (17 L) and a mycelial cake. The filtrate was extracted three times with same volumes of EtOAc (3 × 17 L) to give an EtOAc extract. The mycelial cake was extracted three times with 3 L acetone-water (2:1) by ultrasonication for 2 h. The aqueous acetone solution obtained by filtration was evaporated under reduced pressure to remove acetone. Then, the remaining water layer (3 L) was extracted three times with same volumes of EtOAc (3 × 3 L) to give another EtOAc extract. The EtOAc extracts both from the filtrate and mycelia gave same TLC spot patterns and thus were combined to afford total 20 g of the EtOAc extract. This EtOAc extract showed an inhibitory effect on K562 cells with the IR% value of 64.6% at the 100 µg*/*mL, which was used for the isolation of **1**–**14** in the following experiments.

On the other hand, 200 µL of the G59 spore suspension was inoculated into one cone-shaped 500 mL flask with 200 mL of the same liquid medium and fermented at the same time and same conditions to obtain 200 mL of fermentation broth. The whole broth (200 mL) was extracted as described above for mutant AD-2-1 to afford an EtOAc extract (98 mg), which did not show inhibitory effect on K562 cells (an IR% value of 6.8% at 100 µg*/*mL). This G59 extract was used in the MTT assays and TLC analyses as negative control and also in the HPLC-PDAD-UV and HPLC-ESI-MS analyses for detecting **1**–**14** in the following experiments.

### 3.4. Isolation of Compounds **1**–**14**

In preliminary TLC analyses of the EtOAc extracts of the control G59 strain and mutant AD-2-1, the lipopeptide components containing **1**–**9** were detected in the mutant extract as white spots on the silica gel TLC plates that were developed using CH_2_Cl_2_ (D)−MeOH (M)−ammonia (A) (90:20:2→90:30:2) as developing solvents, but not detected in the G59 extract. Spots of the lipopeptide components were visualized by spraying distilled water onto the plates. The components of polyketides including **10**–**14** were also detected only in the mutant extract as several spots on the silica gel TLC plates developed by dichloromethane (D)−acetone (100:1→1:1) or on the polyamide film developed by MeOH−Water (60:40→90:10). Spots of the polyketide components were examined under sunlight and UV lights (254 and 360 nm) or by using the 5% FeCl_3_ reagent, Vaughan’s reagent or 10% sulfuric acid reagent. Thus, the separation of the mutant extract was performed tracing these newly produced metabolites under the guidance of MTT assay and TLC analysis by direct comparison with the control G59 extract.

The EtOAc extract (20 g) of the mutant AD-2-1 was separated into MeOH soluble (15 g) and insoluble (5 g) parts by dissolving in approximate 200 mL of MeOH. The MeOH insoluble part (5 g) did not show inhibitory effect on K562 cells. The MeOH soluble part (15 g) was dissolved in 100 mL EtOH and subjected to a Sephadex LH-20 column (bed 5 cm × 40 cm in EtOH). Elution of the column using EtOH as eluent afforded five fractions in the order of elution: **Fr-1** (3.6 g), **Fr-2** (3.1 g), **Fr-3** (2.7 g), **Fr-4** (3.4 g) and **Fr-5** (2.2 g). These fractions inhibited the K562 cells with the IR% values of 50.4% (**Fr-1**), 25.5% (**Fr-2**), 18.9% (**Fr-3**), 50.1% (**Fr-4**), and 30.4% (**Fr-5**) at the 100 µg/mL. TLC analyses of the fractions indicated that the aimed metabolites newly produced by the mutant, compared to the control G59 strain, existed in two fractions: The lipopeptide components including **1**–**9** in the fraction **Fr-1** and the polyketide components including **10**–**14** in the fraction **Fr-4**.

**Fr-1** (3.6 g) was further subjected to vacuum liquid chromatography (VLC) on an ODS column (bed 4.8 cm × 10 cm) dry-packed with 25 g of ODS. A stepwise elution with Water (W)–EtOH (E) (100:0→5:95) gave five fractions in the order of elution: **Fr-1-1** (0.32 g, elution of water), **Fr-1-2** (0.15 g, eluted by WE 4:1→7:3), **Fr-1-3** (0.2 g, eluted by WE 7:3), **Fr-1-4** (2.3 g, eluted by WE 3:2→1:1), and **Fr-1-5** (0.6 g, eluted by WE 2:3→3:7). These fractions inhibited the K562 cells with the IR% values of 24.9% (**Fr-1-1**), 11.5% (**Fr-1-2**), 40.3% (**Fr-1-3**), 70.3% (**Fr-1-4**), and 28.9% (**Fr-1-5**) at the 100 µg/mL. TLC analysis indicated that **Fr-1-3** contained **7** and **Fr-1-4** contained **1**–**6**, **8**, and **9**. Thus, **Fr-1-3** (0.2 g) was subjected to preparative TLC (PTLC) on silica gel GF_254_ plates (20 cm × 20 cm, 0.5 mm thickness) using CH_2_Cl_2_ (D)−MeOH (M)−ammonia (A) (90:30:2) as developing solvent. Silica gels on a band with R*_f_* value of 0.6, which was visualized by spraying distilled water, were harvested and adsorbed substances were extracted by eluting with acetone. Further purification of the substances by passing through a small Sephadex LH-20 column in MeOH afforded compound **7** (30 mg) as a white amorphous powder from MeOH solution. **Fr-1-4** (0.5 g) was dissolved in 5 mL MeOH and subjected to PTLC using DMA (90:20:2) as developing solvent. Silica gels on four bands (R*_f_* values: 0.45, 0.5, 0.6, and 0.7) were harvested, respectively, which were visualized by spraying distilled water. Then, substances adsorbed in the silica gels were extracted by eluting with acetone to obtain four fractions: **Fr-1-4-1** (60 mg, R*_f_* ≈ 0.7), **Fr-1-4-2** (210 mg, R*_f_* ≈ 0.6), **Fr-1-4-3** (40 mg, R*_f_* ≈ 0.5), and **Fr-1-4-4** (30 mg, R*_f_* ≈ 0.45). These fractions inhibited the K562 cells with the IR% values of 40.5% (**Fr-1-4-1**), 43.0% (**Fr-1-4-2**), 70.8% (**Fr-1-4-3**), and 37.3% (**Fr-1-4-4**) at the 100 µg/mL. **Fr-1-4-1** (60 mg) was further separated by repeated semi-preparative HPLC (column: a reversed-phase Click β-CD column, 8 mm × 150 mm, column temperature: 40 °C; mobile phase: MeOH–H_2_O 42:58, flow rate: 2 mL/min; detecting wave length: 210 nm) to give compounds **1** (3 mg, *t*_R_ = 48 min), **3** (5 mg, *t*_R_ = 54 min), **2** (2.5 mg, *t*_R_ = 67 min), and **4** (7 mg, *t*_R_ = 76 min). **Fr-1-4-2** (60 mg) and **Fr-1-4-3** (40 mg) were subjected also to repeated semi-preparative HPLC (column: a reversed-phase Click β-CD column, 8 mm × 150 mm, column temperature: 26 °C; mobile phase: MeOH–H_2_O 48:52, flow rate: 2 mL/min; detecting wave length: 210 nm) to obtain compound **8** (13 mg, *t*_R_ = 54 min) from **Fr-1-4-2** and compound 9 (10 mg, *t*_R_ = 42 min) from **Fr-1-4-3**, respectively. On the other hand, repeated semi-preparative HPLC separation (column: a reversed-phase Click β-CD column, 8 mm × 150 mm, column temperature: 26 °C; mobile phase: MeOH–H_2_O 45:55, flow rate: 2 mL/min; detecting wave length: 210 nm) of **Fr-1-4-4** (30 mg) afforded compound **5** (1 mg, *t*_R_ = 42 min) and **6** (2 mg, *t*_R_ = 52 min).

**Fr-4** (3.4 g) was subjected to VLC on a silica gel column (dry-packed with 25 g silica gel, bed2 cm × 10 cm). Stepwise elution of the column with b.p. 60–90 °C Petroleum ether (P)–Dichloromethane (D) (100:0→0:100) and then DM (90:10→0:100) as eluting solvents gave five fractions: **Fr-4-1** (10 mg, eluted by PD 1:1→9:1), **Fr-4-2** (0.8 g, eluted by PD 9:1→1:9), **Fr-4-3** (1.3 g, eluted by D), **Fr-4-4** (1.2 g, eluted by DM 98:2→92:8), and **Fr-4-5** (0.12 g, eluted by DM 92:8→4:1). These fractions inhibited the K562 cells with IR% values of 16.9% (**Fr-4-1**), 49.0% (**Fr-4-2**), 35.9% (**Fr-4-3**), 53.9% (**Fr-4-4**), and 13.2% (**Fr-4-5**) at the 100 µg/mL. Among these, three fractions, **Fr-4-2**, **Fr-4-3** and **Fr-4-4**, were known to contain targeted polyketide components by TLC examination. **Fr-4-2** (0.8 g) was subjected to Polyamide column (bed 2 cm × 30 cm) chromatography and stepwise elution with WM (100:0→0:100) afforded compounds **10** (24 mg) from a fraction eluted by WM (3:7) and **12** (18 mg) from a fraction eluted by WM (1:4). **Fr-4-3** (1.3 g) was separated by Sephadex LH-20 column (bed 2.5 cm × 60 cm in DM 1:1) chromatography (eluting solvent, DM 1:1) to obtain three fractions: **Fr-4-3-1** (0.3 g), **Fr-4-3-2** (0.61 g), and **Fr-4-3-3** (0.24 g). These fractions inhibited the K562 cells with the IR% values of 19.0% (**Fr-4-3-1**), 41.6% (**Fr-4-3-2**), and 10.5% (**Fr-4-3-3**) at the 100 µg/mL. Recrystallization of **Fr-4-3-2 **(0.61 g) in DM (1:1) solution gave compound **14** (400 mg) as yellowish needles. **Fr-4-4** (1.2 g) was separated by Polyamide column (bed 2 cm × 30 cm) chromatography using WM (100:0→0:100) as eluting solvent to give a fraction (0.6 g) eluted by WM (3:7→1:9), which contained targeted polyketides. This fraction (0.6 g) was further separated by VLC on a silica gel column (bed 2.5 cm × 8 cm) using PD (1:3→1:5) as eluting solvent to obtain compounds **11** (13 mg) from PD 1:3 eluent and **13** (20 mg) from PD 1:5 eluent.

### 3.5. Physicochemical and Spectral Data for Compounds **1**–**14**

Penicimutalide A (**1**): White amorphous powder (MeOH), 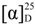 −18.0 (*c* 0.1, MeOH). Positive ESI-MS *m*/*z*: 594 [M + Na]^+^; negative ESI-MS *m*/*z*: 570 [M − H]^−^, 606 [M + Cl]^−^, 616 [M + HCOO]^−^, 660 [M + HC_2_O_4_]^−^. Positive HR-ESI-MS *m*/*z*: measured 594.3834 [M + Na]^+^, calcd for C_28_H_53_N_5_O_7_Na [M + Na]^+^ 594.3843, measured 610.3626 [M + K]^+^, calcd for C_28_H_53_N_5_O_7_Na [M + K]^+^ 610.3582. IR ν_max_ cm^−1^ (KBr): 3422, 3283, 2958, 2922, 2852, 1663, 1626, 1552, 1536, 1516, 1443, 1384, 1311, 1261, 1095, 1018, 928, 879. ^1^H and ^13^C NMR data: see Table 1 and Table 2 and see also Table S1 in the Supplementary Information.

Penicimutalide B (**2**): White amorphous powder (MeOH), 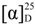 −17.0 (*c* 0.1, MeOH). Positive ESI-MS *m*/*z*: 594 [M + Na]^+^; negative ESI-MS *m*/*z*: 570 [M − H]^−^, 606 [M + Cl]^−^, 616 [M + HCOO]^−^, 660 [M + HC_2_O_4_]^−^. Positive HR-ESI-MS *m*/*z*: measured 594.3847 [M + Na]^+^, calcd for C_28_H_53_N_5_O_7_Na [M + Na]^+^ 594.3843, measured 610.3612 [M + K]^+^, calcd for C_28_H_53_N_5_O_7_K [M + K]^+^ 610.3582. IR ν_max_ cm^−1^ (Diamond ATR crystal): 3283, 3215, 2934, 2862, 1654, 1540, 1419, 1320, 1290, 1255, 1147, 1122, 1063, 883, 865. ^1^H and ^13^C NMR data: see Table 1 and Table 2 and see also Table S2 in the Supplementary Information.

Penicimutalide C (**3**): White amorphous powder (MeOH), 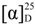 −23.7 (*c* 0.3, MeOH). Positive ESI-MS *m*/*z*: 558 [M + H]^+^, 580 [M + Na]^+^; negative ESI-MS *m*/*z*: 592 [M + Cl]^−^. Positive HR-ESI-MS *m*/*z*: measured 580.3695 [M + Na]^+^, calcd for C_27_H_51_N_5_O_7_Na [M + Na]^+^ 580.3686. IR ν_max_ cm^−1^ (Diamond ATR crystal): 3268, 2934, 2862, 1650, 1536, 1418, 1325, 1282, 1256, 1196, 1145, 1114, 1062, 979, 949. ^1^H and ^13^C NMR data: see Table 1 and Table 2 and see also Table S3 in the Supplementary Information.

Penicimutalide D (**4**): White amorphous powder (MeOH), 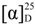 −24.8 (*c* 0.3, MeOH). Positive ESI-MS *m*/*z*: 558 [M + H]^+^, 580 [M + Na]^+^, 596 [M + K]^+^; negative ESI-MS *m*/*z*: 556 [M − H]^−^,592 [M + Cl]^−^. Positive HR-ESI-MS *m*/*z*: measured 580.3684 [M + Na]^+^, calcd for C_27_H_51_N_5_O_7_Na [M + Na]^+^ 580.3686, measured 596.3426 [M + K]^+^, calcd for C_28_H_53_N_5_O_7_K [M + K]^+^ 596.3426. IR ν_max_ cm^−1^ (Diamond ATR crystal): 3274, 2930, 2859, 1650, 1632, 1537, 1417, 1325, 1281, 1255, 1197, 1139, 1112, 1056, 946, 865. ^1^H and ^13^C NMR data: see Table 1 and Table 2 and see also Table S4 in the Supplementary Information.

Penicimutalide E (**5**): White amorphous powder (MeOH), 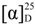 −15.0 (*c* 0.04, MeOH). Positive ESI-MS *m*/*z*: 571 [M + H]^+^; negative ESI-MS *m*/*z*: 569 [M − H]^−^. Positive HR-ESI-MS *m*/*z*: measured 571.3819 [M + H]^+^, calcd for C_27_H_51_N_6_O_7_ [M + H]^+^ 571.3819; measured 593.3634 [M + Na]^+^, calcd for C_27_H_50_N_6_O_7_Na [M + Na]^+^ 593.3639. IR ν_max_ cm^−1^ (Diamond ATR crystal): 3273, 2931, 2859, 1642, 1546, 1537, 1418, 1324, 1283, 1156, 1134, 1074, 864, 783. ^1^H and ^13^C NMR data: see Table 1 and Table 2 and see also Table S5 in the Supplementary Information.

Penicimutalide F (**6**): White amorphous powder (MeOH), 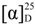 −18.8 (*c* 0.04, MeOH). Positive ESI-MS *m*/*z*: 571 [M + H]^+^; negative ESI-MS *m*/*z*: 569 [M − H]^−^. Positive HR-ESI-MS *m*/*z*: measured 571.3805 [M + H]^+^, calcd for C_27_H_51_N_6_O_7_ [M + H]^+^ 571.3819. IR ν_max_ cm^−1^ (Diamond ATR crystal): 3273, 2933, 2860, 1653, 1628, 1540, 1418, 1177, 1132, 1024. ^1^H and ^13^C NMR data: see Table 1 and Table 2 and see also Table S6 in the Supplementary Information.

Penicimutalide G (**7**): White amorphous powder (MeOH), 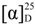 −7.5 (*c* 0.1, MeOH). Positive ESI-MS *m*/*z*: 458 [M + H]^+^, 480 [M + Na]^+^; negative ESI-MS *m*/*z*: 456 [M − H]^−^, 492 [M + Cl]^−^. Positive HR-ESI-MS *m*/*z*: measured 458.2970 [M + H]^+^, calcd for C_21_H_40_N_5_O_6_ [M + H]^+^ 458.2979; measured 480.2798 [M + Na]^+^, calcd for C_21_H_39_N_5_O_6_Na [M + Na]^+^ 480.2798; measured 496.2526 [M + K]^+^, calcd for C_27_H_5_N_5_O_7_K [M + K]^+^ 496.2537. IR ν_max_ cm^−1^ (Diamond ATR crystal): 3274, 3205, 2929, 2857, 1651, 1632, 1540, 1414, 1320, 1281, 1254, 1200, 1133, 1095, 1070, 1042, 958, 866. ^1^H and ^13^C NMR data: see Table 1 and Table 2 and see also Table S7 in the Supplementary Information.

Fellutamide B (**8**): White amorphous powder (MeOH), 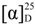 −28.0 (*c* 0.5, MeOH). Positive ESI-MS *m*/*z*: 556 [M + H]^+^; negative ESI-MS*m*/*z*: 554 [M − H]^−^. ^1^H and ^13^C NMR data: see Table 1 and Table 2.

Fellutamide C (**9**): White amorphous powder (MeOH), 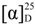 −33.0 (*c* 0.2, MeOH). Positive ESI-MS *m*/*z*: 558 [M + H]^+^, 580 [M + Na]^+^; negative ESI-MS *m*/*z*: 592 [M + Cl]^−^. Positive HR-ESI-MS *m*/*z*: measured 558.3862 [M + H]^+^, calcd for C_27_H_52_N_5_O_7_ [M + H]^+^ 558.3867; measured 580.3692 [M + Na]^+^, calcd for C_27_H_51_N_5_O_7_Na [M + Na]^+^ 580.3686; measured 596.3413 [M + K]^+^, calcd for C_27_H_51_N_5_O_7_K [M + K]^+^ 596.3426. ^1^H and ^13^C NMR data: see Table 1 and Table 2.

1′-*O*-Methylaverantin (**10**): Crystalline orange-red powder (CH_2_Cl_2_), mp 214−216 °C, 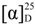 +1.8 (*c* 0.4, MeOH), ESI-MS *m*/*z*: 387 [M + H]^+^. ^1^H NMR (acetone-*d*_6_, 400MHz) δ: 12.78 (1H, s, 1-OH), 12.18 (1H, s, 8-OH), 10.10 (1H, br s, 6-OH), 9.69 (1H, br s, 3-OH) 7.23 (1H, d, *J* = 2.4 Hz, H-5), 7.13 (1H, s, H-4), 6.64 (1H, d, *J* = 2.4 Hz, H-7), 4.97 (1H, dd, *J* = 7.9, 5.1 Hz, H-1′), 3.49 (3H, s, 1′-OCH_3_), 1.95–1.75 (2H, m, H_2_-3′), 1.58–1.38 (2H, m, H_2_-2′), 1.38–1.24 (4H, m, H_2_-4′, H_2_-5′), 0.89 (3H, t, *J* = 6.8 Hz, H_3_-6′).

Averantin (**11**): Crystalline orange-red powder (CH_2_Cl_2_), mp 279−280 °C, 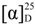 −130.0 (*c* 0.52, MeOH). ESI-MS *m*/*z*: 373 [M + H]^+^. ^1^H NMR (acetone-*d*_6_, 400MHz) δ: 12.63 (1H, s, 1-OH), 12.08 (1H, s, 8-OH), 9.00 (2H, br s, 1-OH, 3-OH), 7.09 (1H, d, *J* = 2.4 Hz, H-5), 7.00 (1H, s, H-4), 6.50 (1H, d, *J* = 2.4 Hz, H-7), 5.29 (1H, dd, *J* = 7.8, 4.5 Hz, H-1′), 1.80–1.63 (2H, m, H_2_-3′), 1.50–1.30 (2H, m, H_2_-2′), 1.30–1.10 (4H, m, H_2_-4′, H_2_-5′), 0.76 (3H, t, *J* = 6.8 Hz, H_3_-6′). ^13^C NMR (acetone-*d*_6_, 100 MHz) δ: 190.7 (C-9), 181.8 (C-10), 166.0 (C-8), 165.9 (C-1), 165.0 (C-6),161.7 (C-3), 136.4 (C-4a), 134.5 (C-10a), 122.0 (C-2), 110.3 (C-5), 110.2 (C-9a), 109.4 (C-8a), 108.9 (C-7), 108.8 (C-4), 69.9 (C-1′), 36.9 (C-2′), 32.3 (C-3′), 25.6 (C-4′), 23.2 (C-5′), 14.3 (C-6′).

Averufin (**12**): Crystalline orange-red powder (CH_2_Cl_2_), mp 280−282 °C, 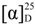 −1.7 (*c* 0.3, CHCl_3_). ^1^H NMR (acetone-*d*_6_, 400MHz) δ: 12.51 (1H, s, 1-OH), 12.19 (1H, s, 8-OH), 10.09 (1H, s, 6-OH), 7.22 (1H, d, *J* = 2.5 Hz, H-5), 7.09 (1H, s, H-4), 6.63 (1H, d, *J* = 2.5 Hz, H-7), 5.29 (1H, d, *J* = 2.8 Hz, H-1′), 2.15–1.25 (6H, m, H_2_-2′,3′,4′), 1.55 (3H, s, H_3_-6′). ^13^C NMR (acetone-*d*_6_, 100MHz) δ: 190.7 (C-9), 181.6 (C-10), 166.0 (C-8), 165.8 (C-1), 161.5 (C-6), 159.8 (C-3), 136.3 (C-4a), 134.4 (C-10a), 117.1 (C-2), 110.2 (C-5), 109.5 (C-9a), 109.4 (C-8a), 108.9 (C-7), 108.4 (C-4), 102.2 (C-5′), 67.4 (C-1′), 36.3 (C-2′), 28.0 (C-4′), 27.9 (C-6′), 16.5 (C-3′).

Nidurufin (**13**): Crystalline orange-red powder (CH_2_Cl_2_), mp 188−190 °C, 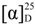 +0.3 (*c* 0.2, CHCl_3_). ESI-MS *m*/*z*: 385 [M + H]^+^. ^1^H NMR (acetone-*d*_6_, 400MHz) δ: 12.58 (1H, s, 1-OH), 12.22 (1H, s, 8-OH), 10.17 (1H, s, 6-OH), 7.26 (1H, d, *J* = 2.5 Hz, H-5), 7.14 (1H, s, H-4), 6.67 (1H, d, *J* = 2.5 Hz, H-7), 5.17 (1H, br s, H-1′), 3.95 (1H, br s, H-2′), 2.30–1.30 (4H, m, H_2_-3′, H_2_-4′), 1.58 (3H, s, H_3_-6′).

Sterigmatocystin (**14**): Pale yellow needles (CH_2_Cl_2_), mp 246 °C (dec.), 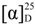 −384 (*c* 0.5, CHCl_3_), ESI-MS *m*/*z*: 325 [M + H]^+^, 347 [M + Na]^+^. ^1^H NMR (400 MHz, CDCl_3_) δ: 13.23(1H, s, 3-OH), 7.47 (1H, dd, *J* = 8.4, 8.0 Hz, H-5), 6.80 (1H, d, *J* = 7.0 Hz, H-14), 6.79 (1H, dd, *J* = 8.0, 1.0 Hz, H-6), 6.72 (1H, dd, *J* = 8.4, 1.0 Hz, H-4), 6.50 (1H, t, *J* = 2.5 Hz, H-17), 6.40 (1H, s, H-11), 5.43 (1H, t, *J* = 2.5 Hz, H-16), 4.76 (1H, dt, *J* = 7.0, 2.5 Hz, H-15), 3.98 (3H, s, H_3_-18). ^13^C NMR (100 MHz, CDCl_3_) δ: 181.2 (C-1), 164.4 (C-10), 163.1 (C-12), 162.1 (C-3), 154.8 (C-7), 153.8 (C-8), 145.2 (C-17), 135.6 (C-5), 113.2 (C-14), 111.1 (C-4), 108.7 (C-2), 106.4 (C-6), 105.8 (C-9), 105.7 (C-13), 102.4 (C-16), 90.4 (C-11), 56.7 (C-18), 47.9 (C-15).

### 3.6. Marfey Analysis for **1–9**

#### 3.6.1. Hydrolysis and Derivatization with FDAA

To nine glass tubes that one terminal has been sealed was added each 50 µL of **1**–**9** solutions in MeOH at 1 mg/mL, respectively. After blown inside the tubes with nitrogen gas to dryness, 100 µL of aqueous 6 N HCl solution was added into each tube. After the open terminal of the tubes was sealed by blast burner, the tubes were kept at 110 °C for 24 h to hydrolyze **1**–**9**. Then, one sealed terminal of the tubes was cut out, reaction solutions were blown with nitrogen gas to dryness, and the tubes were maintained in vacuo overnight to clean up remained HCl. At the same time, each 500 µg of standards in 500 µL of aqueous 6 N HCl solution, l-Asn, d-Asn, l-Gln, d-Gln, l-Leu, d-Leu, l-Leu amide and d-Leu amide, was also hydrolyzed in the same manner and same conditions. At this condition, the l-Leu amide and d-Leu amide were hydrolyzed to l-Leu and d-Leu, respectively. Both hydrolyzates of the standards and **1**–**9** were dissolved in each 10 μL of distilled water, mixed with 10 μL of 10 mM l-FDAA in acetone and 10 μL of 1 N NaHCO_3_ aqueous solution, respectively, and reacted at 45 °C for 1.5 h. The reaction mixtures were neutralized with 5 μL of 2 N HCl, respectively. Then, the reaction mixtures were filtered, and the filtrates were subjected to HPLC analysis.

#### 3.6.2. Oxidization of Leucinal in **8** to Leucine, then Hydrolysis and Derivatization with FDAA

Because we had the l-/d-leucine standards in hand but lacked the l-/d-leucinal standards, in order to determine the absolute configuration of leucinal in **8**, the leucianl in **8** was oxidized into leucine at first. The 100 µg of **8** was suspended in 100 µL distilled water, then 2 µg KMnO_4_ was added, and reacted by stirring at room temperature for 30 min to oxidize the leucianl in **8** into the leucine. The reaction mixture was extracted with *n*-BuOH (250 μL × 2) to obtain an oxidized product of **8** from the *n*-BuOH solution. Then, the total of this oxidized product was hydrolyzed using 100 μL of aqueous 6 N HCl at 110 °C for 24 h, derivatized as mentioned above with FDAA at the same time and same conditions, and then subjected to the HPLC analysis, in the same manner as mentioned.

#### 3.6.3. HPLC Analysis of FDAA Derivatives of **1**–**9** to Determine Absolute Configurations

HPLC analysis of the FDAA derivatives was performed on a Venusil MP C_18_ column (5 µm, 100 Å, 4.6 mm × 250 mm) at room temperature, using mixed A (H_2_O containing 1% HCOOH) and B (acetonitrile) solutions in a linear gradient (15% B at initial time 0 min→50% B at 60 min→100% B at 70 min; flow rate, 1 mL/min) as mobile phase. The acquired photodiode array (PDA) data were processed by Empower PDA software and targeted derivatives were detected at 340 nm.

In the HPLC conditions, the FDAA derivatives of d- and l-standards were separated well as given their retention times (*t*_R_) in Table 4. The FDAA derivatives of **1**–**9** were analyzed by injection of the derivatives alone and co-injection with those of related l- and d-standards, respectively.

**Table 4:** Retention times (*t*_R_) for the FDAA derivatives of l- and d-standards.

FDAA derivative	*t*_R_ (min)	FDAA derivative	*t*_R_ (min)
l-Aspartic acid	28.60	d-Aspartic acid	30.55
l-Glutamic acid	30.85	d-Glutamic acid	33.38
l-leucinol	51.14	d-leucinol	59.53
l-leucine	53.11	d-leucine	59.85

### 3.7. Hydrolysis of **1**–**9** for Determination of the 23R Absolute Configuration in **1**–**9**

Each 0.3 mg of **1**–**9** was mixed, and the mixture was suspended in 1 mL of aqueous 6 N HCl in a sealed tube and hydrolyzed at 110 °C for 1 h. The solution was then extracted three times with 1 mL of CHCl_3_ to obtain a CHCl_3_ extract. This extract was purified by silica gel PTLC (0.5 mm thickness, 20 mm × 20 mm; developing solvent: DM 95:5) to obtain 3*R*-hydroxydodecanoic acid (0.8 mg, R*_f_* = 0.5): 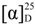 −16 (*c* 0.05, CHCl_3_). Positive ESI-MS *m*/*z*: 239 [M + Na]^+^; negative ESI-MS *m*/*z*: 215 [M − H]^−^. ^1^H NMR (400 MHz, CDCl_3_) δ: 4.02 (1H, m), 2.57 (2H, dd, *J* = 16.6, 3.0 Hz), 2.47 (1H, dd, *J* = 16.6, 9 Hz), 1.40–1.58 (2H, m), 1.20–1.36 (14H, m), 0.87 (3H, t, *J* = 6.6 Hz). ^13^C NMR (CDCl_3_) δ: 176.8, 68.0, 40.8, 36.5, 31.9, 29.7, 29.5, 29.4, 29.3, 25.4, 22.7, 14.1. The 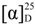 and ^1^H and ^13^C NMR data are identical with those reported for 3*R*-hydroxydodecanoic acid in the literature [24].

### 3.8. HPLC-PDAD-UV Analysis for Detecting **1**–**14** in the G59 and AD-2-1 Extracts

EtOAc extracts of the control G59 strain and the mutant AD-2-1 were dissolved in MeOH to prepare sample solutions at 10 mg/mL for HPLC analysis. Crude samples of the compounds **1**–**14** in MeOH at 10 mg/mL were used as reference standards in the HPLC-PDAD-UV analysis. The MeOH solutions of **1**–**6** at 10 mg/mL were mixed between **1**/**2**, **3**/**4**, and **5**/**6** in a ratio of 1:1, respectively, and the mixed sample solutions were used, because the two compounds in these pairs were known to be eluted as a peak with the same retention time in the given HPLC condition by pilot tests of HPLC analyses.

The HPLC-PDAD-UV analysis was carried out on a Venusil MP C_18_ column (5 µm, 100 Å, 4.6 mm × 250 mm; Agela Technologies) using the Waters HPLC equipment mentioned above. The sample and standard solutions were filtered using 0.22 µm pore membrane filters, respectively, and each 5 µL of the solutions was injected into the column. Then, elution was performed using MeOH–H_2_O in linear gradient (20% MeOH at initial time 0 min→100% MeOH at 60 min→100% MeOH at 90 min; flow rate, 1 mL/min). The acquired photodiode array (PDA) data were processed using the Empower PDA software to obtain targeted HPLC-PDAD-UV data.

In the HPLC-PDAD-UV analysis, examination of **1**–**14** was performed on the basis of both HPLC retention times and UV absorption curves.

Retention times of **1**–**9**: 56.12 min (**1**/**2**), 54.57 min (**3**/**4**), 50.62 min (**5**/**6**), 46.52 min (**7**), 52.77 min (**8**), and 52.57 min (**9**). Because **1**–**9** showed only end UV absorptions, they were hardly identified by the HPLC-PDAD-UV analysis both in the mutant AD-2-1 and the control G59 extracts due to the lack of typical UV absorptions and the baseline drift (Figure S1-A1 and A2 in the Supplementary Information).

Retention times of **10**–**14**: 63.90 min (**10**), 59.50 min (**11**), 63.00 min (**12**), 52.12 min (**13**), and 50.25 min (**14**). All of **10**–**14** were detected in the mutant AD-2-1 extract but not in the parent G59 extract (see Figure 2 and see also Figure S1-B1,B2,C1,C2 in the Supplementary Information).

### 3.9. HPLC-ESI-MS Analysis for Detecting **10**–**14** in the G59 and AD-2-1 Extracts

The MeOH solutions of crude **1**–**14** and the EtOAc extracts of G59 strain and mutant AD-2-1, which were used in the HPLC-PDAD-UV analysis, were used also in HPLC-ESI-MS analysis for detecting **1**–**14** from the EtOAc extracts.

The HPLC-ESI-MS analysis was performed on an LC-MS equipment equipped with Agilent 1100 HPLC system, AB Sciex API 3000 LC-MS/MS system and AB Sciex Analyst 1.4 software. HPLC was carried out on the Venusil MP C_18_ column (5 µm, 100 Å, 4.6 mm × 250 mm; Agela Technologies) at the conditions the same as mentioned for the HPLC-PDAD-UV analysis. The mass detector was set to scan a range from *m*/*z* 150–1500 both in the positive and negative modes. The acquired data were processed by Analyst 1.4 software to obtain targeted HPLC-ESI-MS data.

In the HPLC-ESI-MS analysis, both positive and negative ion peaks of **1**–**14** appeared with shortened retention times than those in their HPLC-PDAD-UV analysis because of the shortened flow length from the outlet of the HPLC column to the inlet of MS. Retention times of **1**–**14**: 52.80 min (**1**/**2**), 51.25 min (**3**/**4**), 47.48 min (**5**/**6**), 43.40 min (**7**), 49.59 min (**8**), and 49.28 min (**9**), 62.38 min (**10**), 58.87 min (**11**), 61.83 min (**12**), 52.05 min (**13**), and 47.74 min (**14**). Detection of **1**–**14** in the control G59 and the mutant AD-2-1 extracts was achieved by selective ion ([M + Na]^+^ for **1**/**2** and **14**; [M − H]^−^ for **5**/**6**, **7**, **8**, and **10**–**13**; and [M + Cl]^−^ for **3**/**4** and **9**) monitoring with both extracted ion chromatograms and related MS spectra (see Figure S2A–K in the Supplementary Information).

## 4. Conclusions

AD-2-1 is an antitumor fungal mutant obtained by the DES mutagenesis of originally inactive, parent *Penicillium purpurogenum* G59 strain. Fourteen metabolites in the mutant AD-2-1 extract, including seven new (**1**–**7**) and two known lipopeptides (**8** and **9**) as well as five known polyketides **10**–**14**, were isolated by tracing newly produced metabolites in the mutant AD-2-1 extract under the guidance of MTT assay and TLC analysis using the parent G59 extract as control. Structures of the seven new compounds including their absolute configurations were determined by spectroscopic data and chemical evidences, and named penicimutalides A–G (**1**–**7**), respectively. All of **1**–**14** inhibited several human cancer cell lines to varying extents. Both present bioassays and HPLC-PDAD-UV and HPLC-ESI-MS analyses demonstrated that the production of **1**–**14** in the mutant AD-2-1 was caused by the activated production of silent metabolites in original fungal G59 strain by the DES mutagenesis.

## Supplementary Files

Supplementary File 1 Supplementary Information (PDF, 5380 KB)
