# Supplementary material for: Seven New and Two Known Lipopeptides as well as Five Known Polyketides: The Activated Production of Silent Metabolites in a Marine-Derived Fungus by Chemical Mutagenesis Strategy Using Diethyl Sulphate
Source: Mar Drugs. 2014 Mar 28;12(4):1815–38. doi: 10.3390/md12041815 (PMC4012460; doi:10.3390/md12041815)

Supplementary Information

Table S1. 600 MHz ^{1}H and 150 MHz ^{13}C NMR data of **1** in DMSO-d_6 [a].

**Position**	δ_C [b,c]	δ_H (*J* in Hz) [b]	**COSY** [d]	**HMBC** [e]
1	—	—	—	—
2	77.2 d	5.07 dt (9.2, 6.9)	Ha-3, Hb-3, H-7 (NH)	C-3, 4, 1'
3	43.3 t	Ha 1.48 dt (13.8, 6.9)	H-2, 4	C-2, 4, 5, 6
		Hb 1.40-1.33 m	H-2, H-4	C-2, 4, 5, 6
4	24.0 d	1.65-1.57 m	Ha-3, Hb-3, H-5, 6	C-2, 3, 5, 6
5	22.3 q	0.83 d (6.8)	H-4, 6	C-3, 4
6	22.4 q	0.86 d (6.7)	H-4, 5	C-3, 4
7 (NH)	—	8.02 d (9.2)	H-2	C-3
8	171.5 s	—	—	
9	52.6 d	4.13 td (7.8, 4.6)	Ha-10, Hb-10, H-14 (NH)	C-8, 11
10	27.6 t	Ha 1.99-1.89 m	H-9, 11	C-8, 9, 11, 12
		Hb 1.78-1.68 m	H-9, 11	C-8, 9, 11, 12
11	31.4 t	2.12-2.04 m	Ha-10, Hb-10	C-9, 10, 12
12	173.7 s	—	—	
13 (NH_2)	—	Ha 7.21 br s	Hb-13 (NH)	C-12
		Hb 6.75 br s	Ha-13 (NH)	C-11, 12
14 (NH)	—	7.94 d (7.8)	H-9	C-9, 10, 15
15	171.0 s	—	—	
16	49.9 d	4.49 q (7.0)	Ha-17, Hb-17, H-20 (NH)	C-15, 17, 18
17	36.9 t	Ha 2.54 dd (15.6, 7.0)	H-16, Hb-17	C-15, 16, 18
		Hb 2.43 dd (15.6, 7.0)	H-16, Ha-17	C-15, 16, 18
18	171.6 s	—	—	
19 (NH_2)	—	Ha 7.37 br s	Hb-19 (NH)	C-18
		Hb 6.90 br s	Ha-19 (NH)	C-17, C-18
20 (NH)	—	8.08 d (7.0)	H-16	C-16, 17, 21
21	171.2 s	—	—	
22	43.4 t	2.26-2.16 m	H-23	C-23, 24
23	67.4 d	3.82-3.75 m	H-22, 24, 23-O<u>H</u>	C-25
24	36.8 t	1.40-1.33 m	H-23, Ha-25, Hb-25	C-25, 26
25	25.1 t	Ha 1.40-1.33 m	H-24, Hb-25, H-26	C-23, C-24, C-26
		Hb 1.30-1.18 m	H-24, Ha-25, H-26	
26	29.1t	1.30-1.18 m		
27	29.1t	1.30-1.18 m		
28	29.0 t	1.30-1.18 m		
29	28.7 t	1.30-1.18m		
30	31.3 t	1.30-1.18 m		
31	22.0 t	1.30-1.18 m		
32	13.9 q	0.85 t (7.0)	H-31	C-31
1'	61.8 t	Ha 3.44 dq (14.1, 7.0)	Hb-1', H-2'	C-2, 2'
		Hb 3.27 dq (14.1, 7.0)	Ha-1', H-2'	C-2, 2'
2'	15.0 q	1.05 t (7.0)	Ha-1', Hb-1'	C-1'
23-O<u>H</u>	—	4.64 d (4.8)	H-23	C-22, 23, 24

[a] Signals assignments were based on the results of DEPT, ^{1}H-^{1}H COSY, HMQC, and HMBC experiments. [b] Chemical shift values (δ_H and δ_C) were recorded using the solvent signals (DMSO-d_6: δ_H 2.50/δ_C 39.52) as references, respectively. [c] Multiplicities of the carbon signals were determined by DEPT experiments and are indicated as s (singlet), d (doublet), t (triplet) and q (quartet), respectively. [d] The numbers in each line of this column indicate the protons that correlated with the proton in the corresponding line in ^{1}H-^{1}H COSY. [e] The numbers in each line of this column indicate the carbons that showed HMBC correlations with the proton in the corresponding line in the HMBC experiments optimized for the 8.3 Hz of long-range J_{CH} value.

Table S2. 600 MHz ^{1}H and 150 MHz ^{13}C NMR data of **2** in DMSO-d_6 [a].

**Position**	**δ_C** [b,c]	**δ_H (*J* in Hz)** [b]	**COSY** [d]	**HMBC** [e]
1	—	—	—	—
2	77.3 d	5.06 dt (9.1, 6.9)	Ha-3, Hb-3, H-7 (NH)	C-3, 4, 1'
3	43.1 t	Ha 1.45 dt (13.8, 6.9)	H-2, 4	C-2, 4, 5, 6
		Hb 1.40 dt (13.8, 6.9)	H-2, 4	C-2, 4, 5, 6
4	23.9 d	1.60-1.52 m	Ha-3, Hb-3, H-5, 6	C-2, 3, 5, 6
5	22.2 q	0.81 d (6.6)	H-4, 6	C-3, 4
6	22.5 q	0.84 d (6.8)	H-4, 5	C-3, 4
7 (NH)	—	8.04 d (9.1)	H-2	C-3
8	171.6 s	—	—	—
9	52.8 d	4.13-4.05 m	Ha-10, Hb-10, H-14 (NH)	C-8, 11
10	27.5 t	Ha 1.97-1.90 m	H-9, 11	C-8, 9, 11, 12
		Hb 1.78-1.70 m	H-9, 11	C-8, 9, 11, 12
11	31.4 t	2.13-2.03 m	Ha-10, Hb-10	C-9, 10, 12
12	173.7 s	—	—	—
13 (NH_2)	—	Ha 7.20 br s	Hb-13 (NH)	C-12
		Hb 6.74 br s	Ha-13 (NH)	C-11, 12
14 (NH)	—	8.06 d (7.6)	H-9	C-9, 10, 15
15	171.1 s	—	—	—
16	49.7 d	4.50 q (7.0)	Ha-17, Hb-17, H-20 (NH)	C-15, 17, 18
17	37.0 t	Ha 2.55 dd (15.6, 7.0)	H-16, Hb-17	C-15, 16, 18
		Hb 2.43 dd (15.6, 7.0)	H-16, Ha-17	C-15, 16, 18
18	171.7 s	—	—	—
19 (NH_2)	—	Ha 7.40 br s	Hb-19 (NH)	C-18
		Hb 6.94 br s	Ha-19 (NH)	C-17, 18
20 (NH)	—	8.05 d (7.0)	H-16	C-16, 17, 21
21	171.1 s	—	—	—
22	43.4 t	2.25-2.16 m	H-23	C-23, 24
23	67.4 d	3.80-3.75 m	H-22, 24, 23-O<u>H</u>	C-25
24	36.8 t	1.39-1.30 m	H-23, Ha-25, Hb-25	C-25, 26
25	25.1 t	Ha 1.39-1.30 m	H-24, Hb-25, H-26	C-23, C-24, C-26
		Hb 1.30-1.18 m	H-24, Ha-25, H-26	
26	29.1t	1.30-1.18 m		
27	29.1t	1.30-1.18 m		
28	29.0 t	1.30-1.18 m		
29	28.7 t	1.30-1.18 m		
30	31.3 t	1.30-1.18 m		
31	22.0 t	1.30-1.18 m		
32	13.9 q	0.86 t (7.2)	H-31	C-30, 31
1'	61.8 t	Ha 3.46 dq (14.1, 7.0)	Hb-1', H-2'	C-2, 2'
		Hb 3.28 dq (14.1, 7.0)	Ha-1', H-2'	C-2, 2'
2'	15.1 q	1.05 t (7.0)	Ha-1', Hb-1'	C-1'
23-O<u>H</u>	—	4.62 d (4.8)	H-23	C-22, 23, 24

[a] Signals assignments were based on the results of DEPT, ^{1}H-^{1}H COSY, HMQC, and HMBC experiments. [b] Chemical shift values (δ_H and δ_C) were recorded using the solvent signals (DMSO-d_6: δ_H 2.50/δ_C 39.52) as references, respectively. [c] Multiplicities of the carbon signals were determined by DEPT experiments and are indicated as s (singlet), d (doublet), t (triplet) and q (quartet), respectively. [d] The numbers in each line of this column indicate the protons that correlated with the proton in the corresponding line in ^{1}H-^{1}H COSY. [e] The numbers in each line of this column indicate the carbons that showed HMBC correlations with the proton in the corresponding line in the HMBC experiments optimized for the 8.3 Hz of long-range J_{CH} value.

Table S3. 400 MHz ^{1}H and 100 MHz ^{13}C NMR data of **3** in DMSO-d_6 [a].

Position	δ_C [b,c]	δ_H (J in Hz) [b]	COSY [d]	HMBC [e]
1	—	—	—	—
2	78.9 d	4.97 dt (9.1, 6.9)	Ha-3, Hb-3, H-7 (NH)	C-1'
3	43.0 t	Ha 1.49 dt (13.8, 6.9)	H-2, 4	C-2, 4, 5, 6
		Hb 1.42-1.30 m	H-2, 4	C-2, 4, 5, 6
4	24.1 d	1.65-1.54 m	Ha-3, Hb-3, H-5, 6	C-2, 3, 5, 6
5	22.4 q	0.83 d (6.7)	H-4, 6	C-3, 4
6	22.5 q	0.85 d (6.7)	H-4, 5	C-3, 4
7 (NH)	—	8.03 d (9.1)	H-2	C-8
8	171.7 s	—	—	—
9	52.8 d	4.18-4.08 m	H-10, H-14 (NH)	C-8
10	27.6 t	Ha 2.02-1.88 m	H-9, Ha-10, H-11	
		Hb 1.80-1.68 m	H-9, Hb-10, H-11	C-9, 11
11	31.5 t	2.14-2.03 m	Ha-10, Hb-10	C-9, 10, 12
12	173.9 s	—	—	—
13 (NH_2)	—	Ha 7.25 br s	Hb-13 (NH)	
		Hb 6.79 br s	Ha-13 (NH)	C-11
14 (NH)	—	8.05 d (8.0)	H-9	C-9, 15
15	171.3 s	—	—	—
16	50.0 d	4.49 q (6.9)	Hb-17, Ha-17, H-20 (NH)	C-15, 17, 18
17	36.9 t	Ha 2.54 dd (15.5, 6.9)	H-16, Hb-17	C-15, 16, 18
		Hb 2.43 dd (15.5, 6.9)	H-16, Ha-17	C-15, 16, 18
18	171.8 s	—	—	—
19 (NH_2)	—	Ha 7.41 br s	Hb-19 (NH)	
		Hb 6.93 br s	Ha-19 (NH)	C-17
20 (NH)	—	8.12 d (6.9)	H-16	C-16, 21
21	171.2 s	—	—	—
22	43.5 t	2.27-2.16 m	H-23	C-21, 23, 24
23	67.5 d	3.84-3.73 m	H-22, 24, 23-O<u>H</u>	C-22
24	37.0 t	1.42-1.30 m	H-23, Ha-25, Hb-25	C-25
25	25.2 t	Ha 1.42-1.30 m	H-24, Hb-25, H-26	
		Hb 1.30-1.18 m	H-24, Ha-25, H-26	C-24
26	29.2 t	1.30-1.18 m		
27	29.1 t	1.30-1.18 m		
28	29.1 t	1.30-1.18 m		
29	28.8 t	1.30-1.18 m		
30	31.4 t	1.30-1.18 m		
31	22.2 t	1.30-1.18 m		
32	14.0 q	0.85 t (7.2)	H-31	C-30, 31
1'	54.3 q	3.11 s		C-2
23-O<u>H</u>	—	4.68 d (4.8)	H-23	C-22, 23, 24

[a] Signals assignments were based on the results of DEPT, ^{1}H-^{1}H COSY, HMQC, and HMBC experiments. [b] Chemical shift values (δ_H and δ_C) were recorded using the solvent signals (DMSO-d_6: δ_H 2.50/δ_C 39.52) as references, respectively. [c] Multiplicities of the carbon signals were determined by DEPT experiments and are indicated as s (singlet), d (doublet), t (triplet) and q (quartet), respectively. [d] The numbers in each line of this column indicate the protons that correlated with the proton in the corresponding line in ^{1}H-^{1}H COSY. [e] The numbers in each line of this column indicate the carbons that showed HMBC correlations with the proton in the corresponding line in the HMBC experiments optimized for the 8.3 Hz of long-range J_{CH} value.

Table S4. 400 MHz ^{1}H and 100 MHz ^{13}C NMR data of **4** in DMSO-d_6 [a].

Position	δ_C [b,c]	δ_H (J in Hz) [b]	COSY [d]	HMBC [e]
1	—	—	—	—
2	78.9 d	4.96 dt (9.4, 7.2)	H-3, H-7 (NH)	C-1'
3	42.8 t	1.49-1.38 m	H-2, 4	C-2, 5, 6
4	24.0 d	1.61-1.49 m	H-3, 5, 6	C-2, 3, 5, 6
5	22.2 q	0.81 d (6.6)	H-4, 6	C-3, 4
6	22.7 q	0.84 d (6.6)	H-4, 5	C-3, 4
7 (NH)	—	8.04 d (9.4)	H-2	C-8
8	171.8 s	—	—	—
9	53.9 d	4.13-4.05 m	Ha-10, Hb-10, H-14 (NH)	C-8
10	27.5 t	Ha 2.00-1.89 m	H-9, Ha-10, H-11	
		Hb 1.81-1.69 m	H-9, Hb-10, H-11	C-9, 11
11	31.5 t	2.13-2.05 m	Ha-10, Hb-10	C-9, 10, 12
12	173.8 s	—	—	—
13 (NH_2)	—	Ha 7.24 br s	Hb-13 (NH)	
		Hb 6.78 br s	Ha-13 (NH)	C-11
14 (NH)	—	8.12 d (7.6)	H-9	C-15
15	171.2 s	—	—	—
16	49.8 d	4.50 q (7.0)	Hb-17, Ha-17, H-20 (NH)	C-15, 17, 18
17	36.9 t	Ha 2.55 dd (15.6, 7.0)	H-16, Hb-17	C-15, 16, 18
		Hb 2.42 dd (15.6, 7.0)	H-16, Ha-17	C-15, 16, 18
18	171.9 s	—	—	—
19 (NH_2)	—	Ha 7.43 br s	Hb-19 (NH)	
		Hb 7.24 br s	Ha-19 (NH)	C-17
20 (NH)	—	8.08 d (7.0)	H-16	C-21
21	171.1 s	—	—	—
22	43.5 t	2.25-2.16 m	H-23	C-21, 23, 24
23	67.5 d	3.82-3.72 m	H-22, 24, 23-O<u>H</u>	
24	37.1 t	1.38-1.30 m	H-23, Ha-25, Hb-25	C-25
25	25.2 t	Ha 1.38-1.30 m	H-24, Hb-25, H-26	
		Hb 1.30-1.18 m	H-24, Ha-25, H-26	
26	29.2 t	1.30-1.18 m		
27	29.2 t	1.30-1.18 m		
28	29.1 t	1.30-1.18 m		
29	28.8 t	1.30-1.18 m		
30	31.4 t	1.30-1.18 m		
31	22.2 t	1.30-1.18 m		
32	14.0 q	0.86 t (6.8)	H-31	C-31
1'	54.4 q	3.12 s		C-2
23-O<u>H</u>	—	4.65 d (5.1)	H-23	C-23

[a] Signals assignments were based on the results of DEPT, ^{1}H-^{1}H COSY, HMQC, and HMBC experiments. [b] Chemical shift values (δ_H and δ_C) were recorded using the solvent signals (DMSO-d_6: δ_H 2.50/δ_C 39.52) as references, respectively. [c] Multiplicities of the carbon signals were determined by DEPT experiments and are indicated as s (singlet), d (doublet), t (triplet) and q (quartet), respectively. [d] The numbers in each line of this column indicate the protons that correlated with the proton in the corresponding line in ^{1}H-^{1}H COSY. [e] The numbers in each line of this column indicate the carbons that showed HMBC correlations with the proton in the corresponding line in the HMBC experiments optimized for the 8.3 Hz of long-range J_{CH} value.

Table S5. 600 MHz ^{1}H and 150 MHz ^{13}C NMR data of **5** in DMSO-d_6 [a].

Position	δ_C [b,c]	δ_H (*J* in Hz) [b]	Position	δ_C [b,c]	δ_H (*J* in Hz) [b]
1	174.2 s	—	18	171.8 s	—
2	49.9 d	4.07-4.02 m	19 (NH_2)	—	Ha 7.46 br s<br>Hb 6.95 br s
3	40.03	Ha 1.59-1.50 m<br>Hb 1.48-1.42 m	20 (NH)	—	8.26 d (7.2)
4	24.2 d	1.59-1.50 m	21	171.2 s	—
5	21.2 q	0.80 d (6.6)	22	43.4 t	2.24-2.16 m
6	23.0 q	0.87 d (6.6)	23	67.4 d	3.80-3.74 m
7 (NH)	—	7.86 d (8.4)	24	37.0 t	1.40-1.18 m
8	170.9 s	—	25	25.0 t	1.40-1.18 m
9	53.2 d	4.13-4.09 m	26	29.1 t	1.40-1.18 m
10	27.1 t	Ha 1.95-1.88 m<br>Hb 1.78-1.72 m	27	29.1 t	1.40-1.18 m
11	31.4 t	2.12-2.04 m	28	28.9 t	1.40-1.18 m
12	173.8 s	—	29	28.7 t	1.40-1.18 m
13 (NH_2)	—	Ha 7.22 br s<br>Hb 6.76 br s	30	31.2 t	1.40-1.18 m
14 (NH)	—	8.10 d (7.2)	31	22.0 t	1.40-1.18 m
15	170.9 s	—	32	13.9 q	0.85 t (6.9)
16	49.9 d	4.48 q (7.2)	1' (NH_2)	—	Ha 6.97 br s<br>Hb 6.95 br s
17	36.9 t	2.58-2.42 m	23-OH	—	4.60 d (4.8)

[a] The ^{1}H and ^{13}C NMR signals of **5** in this Table were assigned by the comparison with the data of its epimer **6**. The 600 MHz ^{1}H and 150 MHz ^{13}C NMR data of **6** in DMSO-d_6 are given in Table S6 in this Supplementary Data. [b] Chemical shift values (δ_H and δ_C) were recorded using the solvent signals (DMSO-d_6: δ_H 2.50/δ_C 39.52) as references, respectively. [c] Multiplicities of the carbon signals were determined by DEPT experiments and are indicated as s (singlet), d (doublet), t (triplet) and q (quartet), respectively.

Table S6. 600 MHz ^{1}H and 150 MHz ^{13}C NMR data of **6** in DMSO-d_6 [a].

**Position**	δ_C [b,c]	δ_H (*J* in Hz) [b]	**COSY** [d]	**HMBC** [e]
1	174.3 s	—	—	—
2	48.7 d	4.15-4.07 m	Hb-1, H-3, H-7 (NH)	C-1, 3
3	40.2 t	Ha 1.59-1.48 m	H-2, Hb-3, H-4	C-2, 4, 5, 6
		Hb 1.48-1.40 m	H-2, Ha-3, H-4	C-2, 4, 5, 6
4	24.1 d	1.59-1.48 m	H-3, 5, 6	C-2, 3, 5, 6
5	21.8 q	0.79 d (6.2)	H-4, 6	C-2, 3, 4, 6
6	23.4 q	0.84 d (5.6)	H-4, 5	C-2, 3, 4, 5
7 (NH)	—	7.87 d (8.4)	H-2	C-2, 3, 8
8	170.5 s	—	—	—
9	52.6 d	4.15-4.07 m	H-10, H-14 (NH)	C-8, 10, 11
10	27.7 t	Ha 2.00-1.92 m	H-9, Ha-10, H-11	C-9, 11, 12
		Hb 1.77-1.67 m	H-9, Hb-10, H-11	C-9, 11, 12
11	31.5 t	2.04 t (7.5)	Ha-10, Hb-10	C-9, 10, 12
12	173.9 s	—	—	—
13 (NH_2)	—	Ha 7.18 br s	Hb-13 (NH_2)	C-12
		Hb 6.71 br s	Ha-13 (NH_2)	C-11, 12
14 (NH)	—	8.09 d (8.4)	H-9	C-9, 10, 15
15	170.9 s	—	—	—
16	49.8 d	4.45 q (6.9)	Hb-17, Ha-17, H-20 (NH)	C-15, 17
17	36.8 t	Ha 2.54 dd (15.6, 6.9)	H-16, Hb-17	C-16, 18
		Hb 2.43 dd (15.6, 6.9)	H-16, Ha-17	C-16, 18
18	171.8 s	—	—	—
19 (NH_2)	—	Ha 7.41 br s	Hb-19 (NH)	C-18
		Hb 6.91 br s	Ha-19 (NH)	C-17, 18
20 (NH)	—	8.15 d (7.2)	H-16	C-16, 17, 21
21	171.2 s	—	—	—
22	43.4 t	2.25-2.15 m	H-23	C-21, 23, 24
23	67.4 d	3.77 m	H-22, 24, 23-O<u>H</u>	
24	36.9 t	1.40-1.18 m	H-23, Ha-25, Hb-25	C-23, C-25
25	25.1 t	1.40-1.18 m	H-24, Hb-25, H-26	C-23, C-26
26	28.7 t	1.40-1.18 m		
27	28.9 t	1.40-1.18 m		
28	29.0 t	1.40-1.18 m		
29	29.1 t	1.40-1.18 m		
30	31.3 t	1.40-1.18 m		
31	22.1 t	1.40-1.18 m		
32	13.9 q	0.84 t (6.9)	H-31	C-31
1' (NH_2)	—	Ha 7.17 br s	Hb-1' (NH)	C-1
		Hb 6.97 br s	Ha-1' (NH)	C-1, 2
23-O<u>H</u>	—	4.66 br s	H-23	

[a] Signals assignments were based on the results of DEPT, ^{1}H-^{1}H COSY, HMQC, and HMBC experiments. [b] Chemical shift values (δ_H and δ_C) were recorded using the solvent signals (DMSO-d_6: δ_H 2.50/δ_C 39.52) as references, respectively. [c] Multiplicities of the carbon signals were determined by DEPT experiments and are indicated as s (singlet), d (doublet), t (triplet) and q (quartet), respectively. [d] The numbers in each line of this column indicate the protons that correlated with the proton in the corresponding line in ^{1}H-^{1}H COSY. [e] The numbers in each line of this column indicate the carbons that showed HMBC correlations with the proton in the corresponding line in the HMBC experiments optimized for the 8.3 Hz of long-range J_{CH} value.

Table S7. 400 MHz ^{1}H and 100 MHz ^{13}C NMR data of **7** in DMSO-d_6 [a].

**Position**	δ_C [b,c]	δ_H (*J* in Hz) [b]	**COSY** [d]	**HMBC** [e]
1	—	—	—	—
2	—	—	—	—
3	—	—	—	—
4	—	—	—	—
5	—	—	—	—
6	—	—	—	—
7 (NH_2)	—	Ha 7.34 br s	Hb-7 (NH)	C-8
		Hb 7.08 br s	Ha-7 (NH)	C-9
8	173.8 s	—	—	—
9	52.3 d	4.06 td (8.0, 4.6)	Ha-10, Hb-10, H-8 (NH)	
10	27.5 t	Ha 1.95 dtd (13.2, 7.5, 4.6)	H-9, 11	C-11
		Hb 1.70 ddt (13.2, 8.0, 7.5)	H-9, 11	C-8, 9, 11
11	31.5 t	2.06 t (7.5)	Ha-10, Hb-10	C-8, 9, 11
12	173.3 s	—	—	—
13 (NH_2)	—	Ha 7.21 br s	Hb-13 (NH)	
		Hb 6.75 br s	Ha-13 (NH)	C-11
14 (NH)	—	7.98 d (8.0)	H-9	C-15
15	170.8 s	—	—	—
16	49.8 d	4.48 q (7.0)	Ha-17, Hb-17, H-20 (NH)	C-15, 17, 18
17	36.9 t	Ha 2.57 dd (15.5, 7.0)	H-16, Hb-17	C-15, 16, 18
		Hb 2.43 dd (15.5, 7.0)	H-16, Ha-17	C-15, 16, 18
18	171.9 s	—	—	—
19 (NH_2)	—	Ha 7.42 br s	Hb-19 (NH)	
		Hb 6.96 br s	Ha-19 (NH)	C-17
20 (NH)	—	8.09 d (7.0)	H-16	C-16, 21
21	171.1 s	—	—	—
22	43.5 t	2.25-2.14 m	H-23	C-21, 23
23	67.5 d	3.82-3.73 m	H-22, 24, 23-O<u>H</u>	
24	36.9 t	1.40-1.20 m	H-23	C-25
25	25.1 t	1.40-1.20 m		
26	28.7 t	1.40-1.20 m		
27	29.0 t	1.40-1.20 m		
28	29.1 t	1.40-1.20 m		
29	29.1 t	1.40-1.20 m		
30	31.3 t	1.40-1.20 m		
31	22.1 t	1.40-1.20 m		
32	14.0 q	0.86 t (6.8)	H-31	C-30, 31
23-O<u>H</u>	—	4.65 d (5.1)	H-23	C-22, 23, 24

[a] Signals assignments were based on the results of DEPT, ^{1}H-^{1}H COSY, HMQC, and HMBC experiments. [b] Chemical shift values (δ_H and δ_C) were recorded using the solvent signals (DMSO-d_6: δ_H 2.50/δ_C 39.52) as references, respectively. [c] Multiplicities of the carbon signals were determined by DEPT experiments and are indicated as s (singlet), d (doublet), t (triplet) and q (quartet), respectively. [d] The numbers in each line of this column indicate the protons that correlated with the proton in the corresponding line in ^{1}H-^{1}H COSY. [e] The numbers in each line of this column indicate the carbons that showed HMBC correlations with the proton in the corresponding line in the HMBC experiments optimized for the 8.3 Hz of long-range J_{CH} value.

Figure S1. HPLC-PDAD-UV analysis of the AD-2-1 and G59 extracts for detecting **1**–**14**.

A1: HPLC profiles of **1**–**9** and the EtOAc extracts of mutant AD-2-1 and parent G59 strain

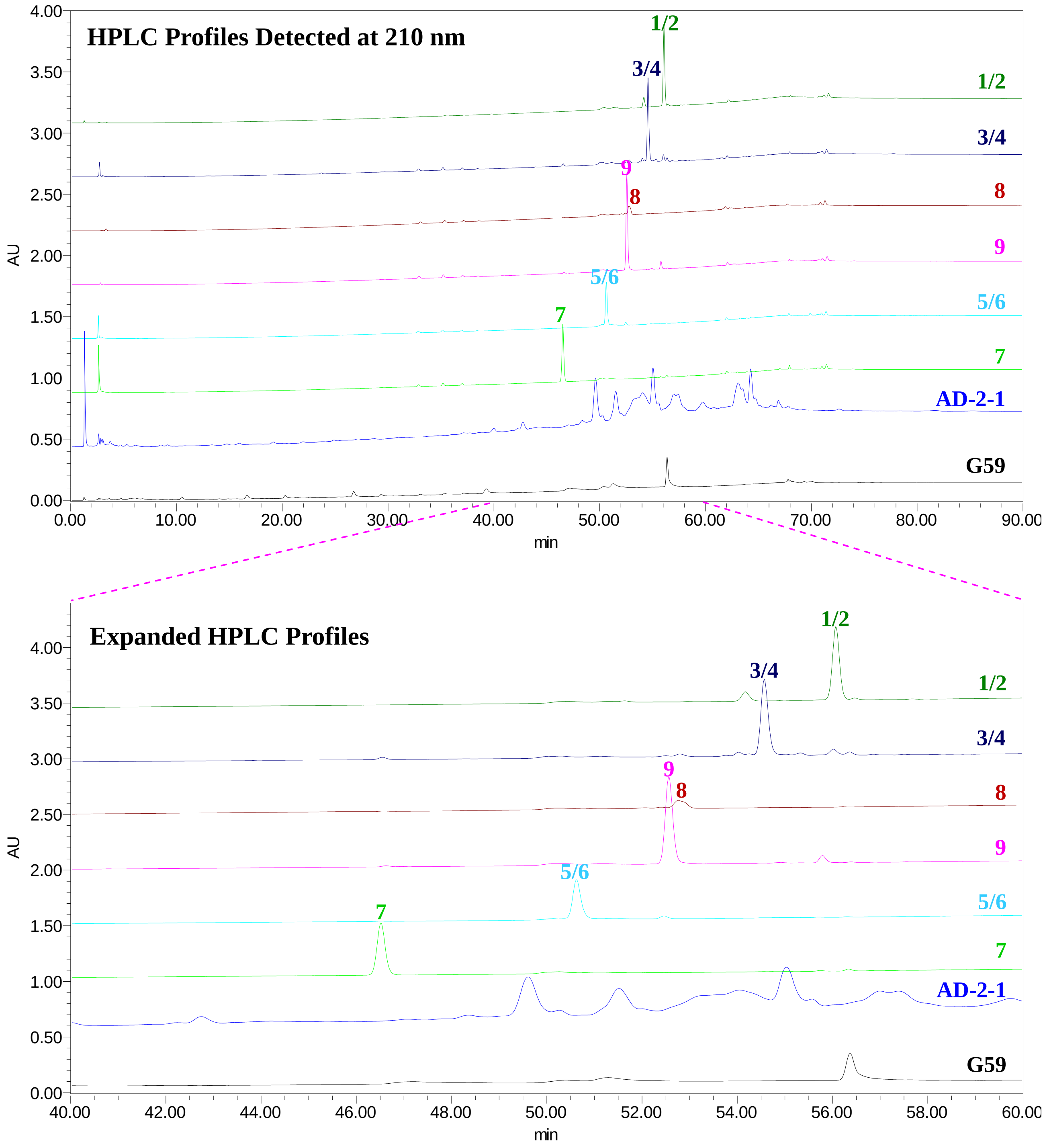

Figure S1. *Cont.*

A2: UV spectra of **1-9** and the AD-2-1and G59 extracts at the same retention time (t_R)

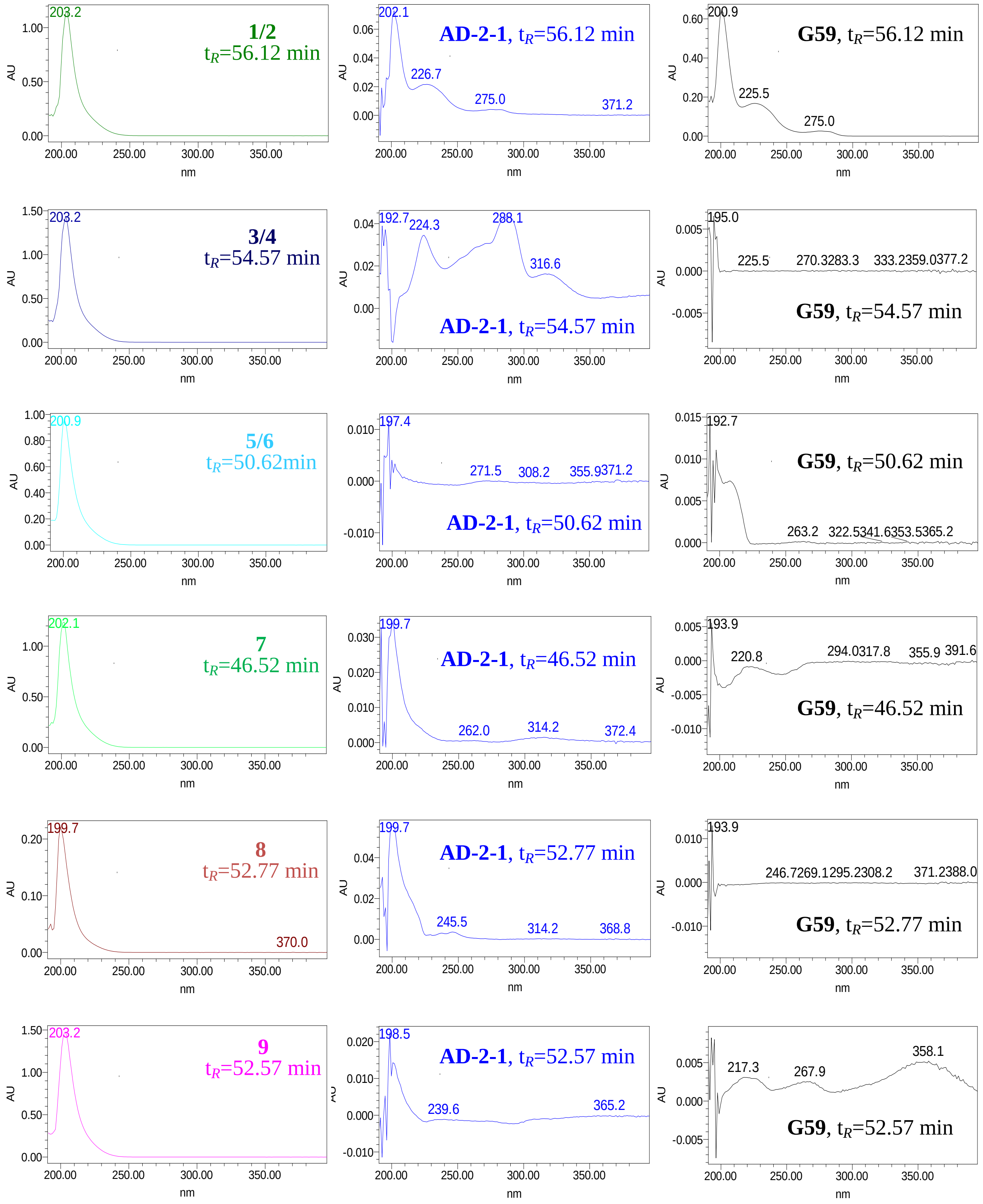

Figure S1. *Cont.*

B1: HPLC profiles of **10–13** and the EtOAc extracts of mutant AD-2-1 and parent G59 strain

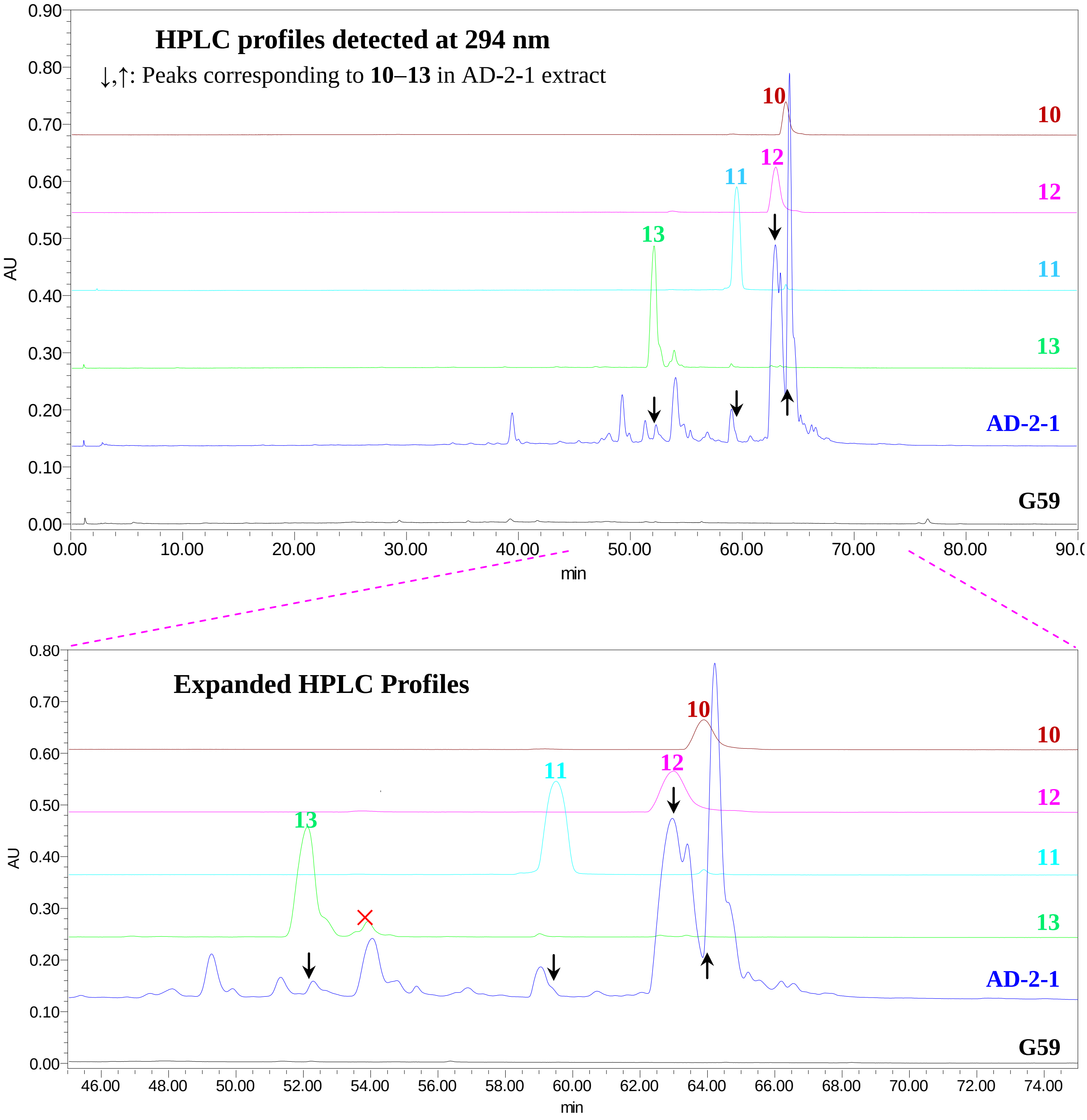

Figure S1. *Cont.*

B2: UV spectra of **10-13** and the AD-2-1 and G59 extracts at the same retention times (t_R)

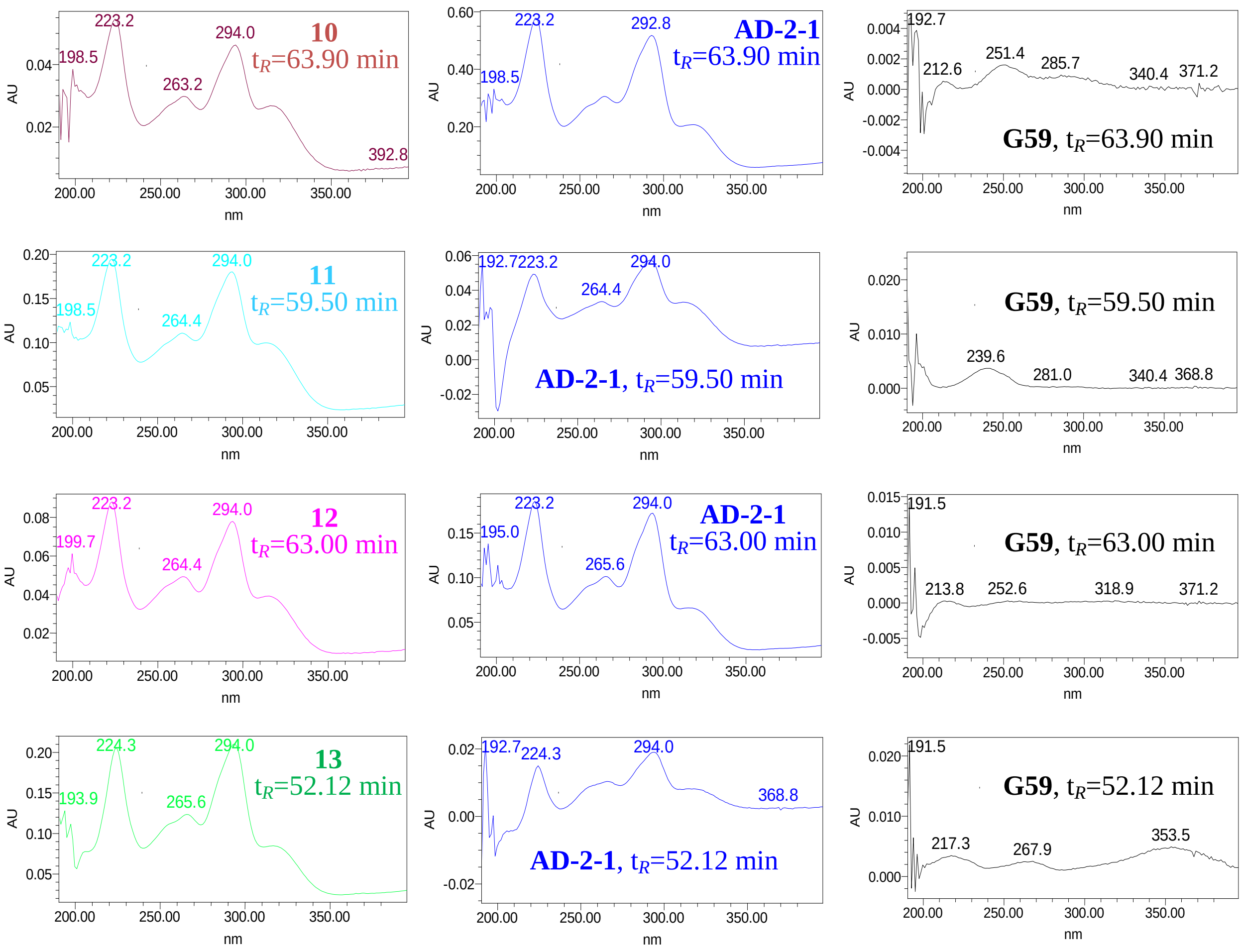

Figure S1. *Cont.*

C1: HPLC profiles of **14** and the EtOAc extracts of mutant AD-2-1 and parent G59 strain

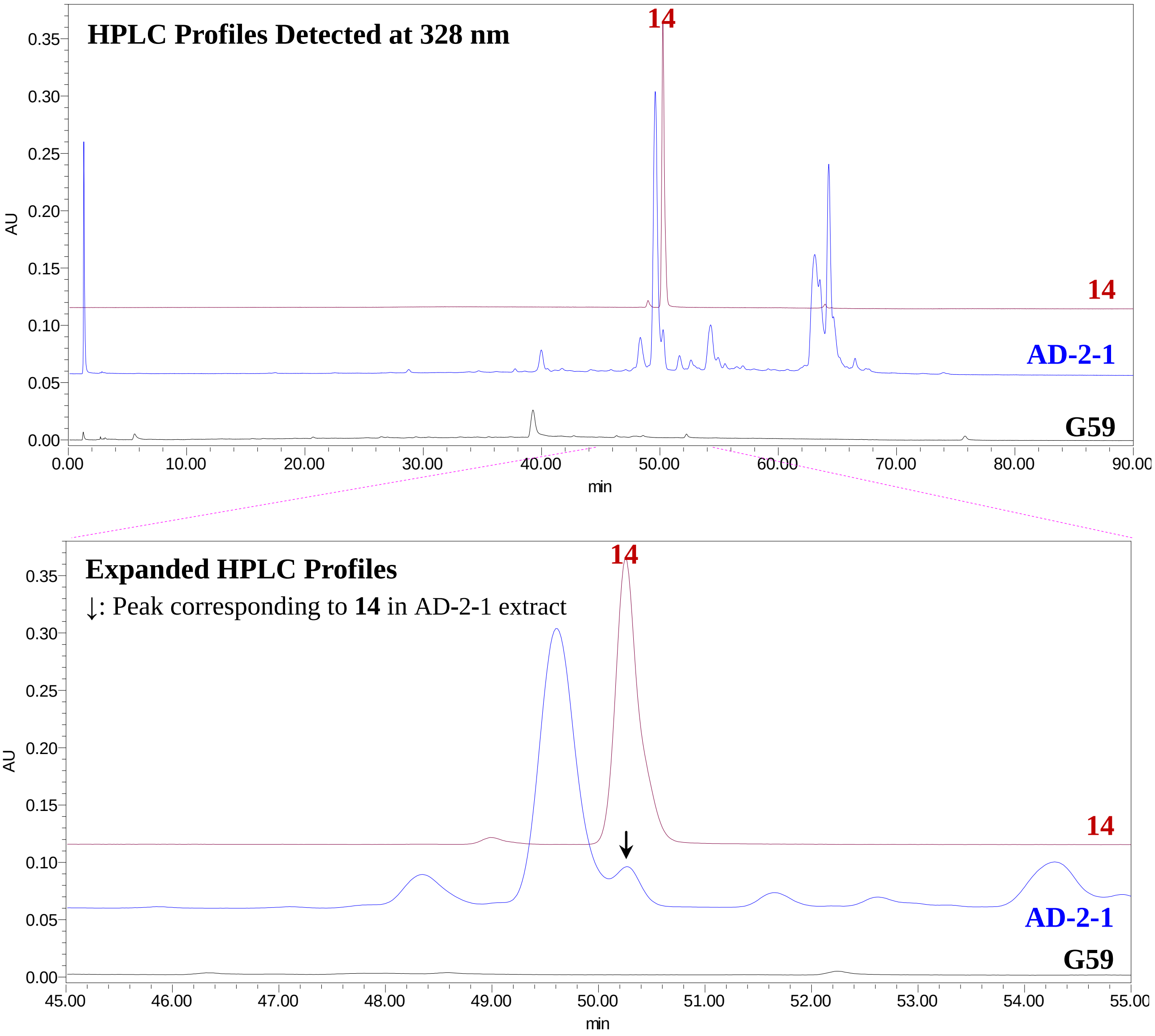

C2: UV spectra of **14** and the AD-2-1 and G59 extracts at the same retention times (t_R)

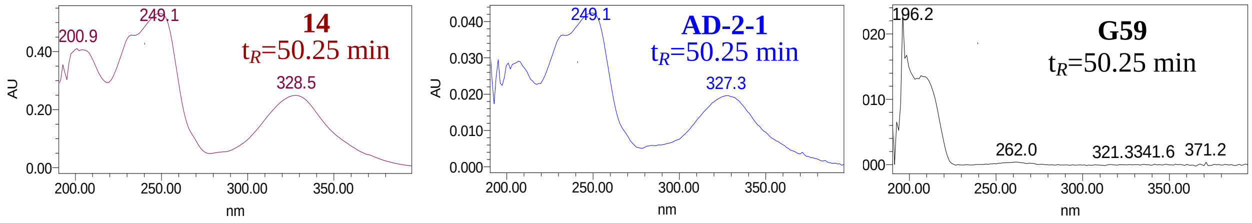

Figure S2. HPLC-ESI-MS analysis of EtOAc extracts of the strain **G59** and the mutant **AD-2-1** for detecting **1–14**.

A: HPLC-Positive ion ESI-MS analysis (ESIMS *m/z*: 594 [M+Na]$^{+}$ for both **1** and **2**)

Extracted Positive Ion Chromatograms (*m/z* 594.0-595.0) ESIMS Spectra in the Range of 52.652-52.978 min Retention

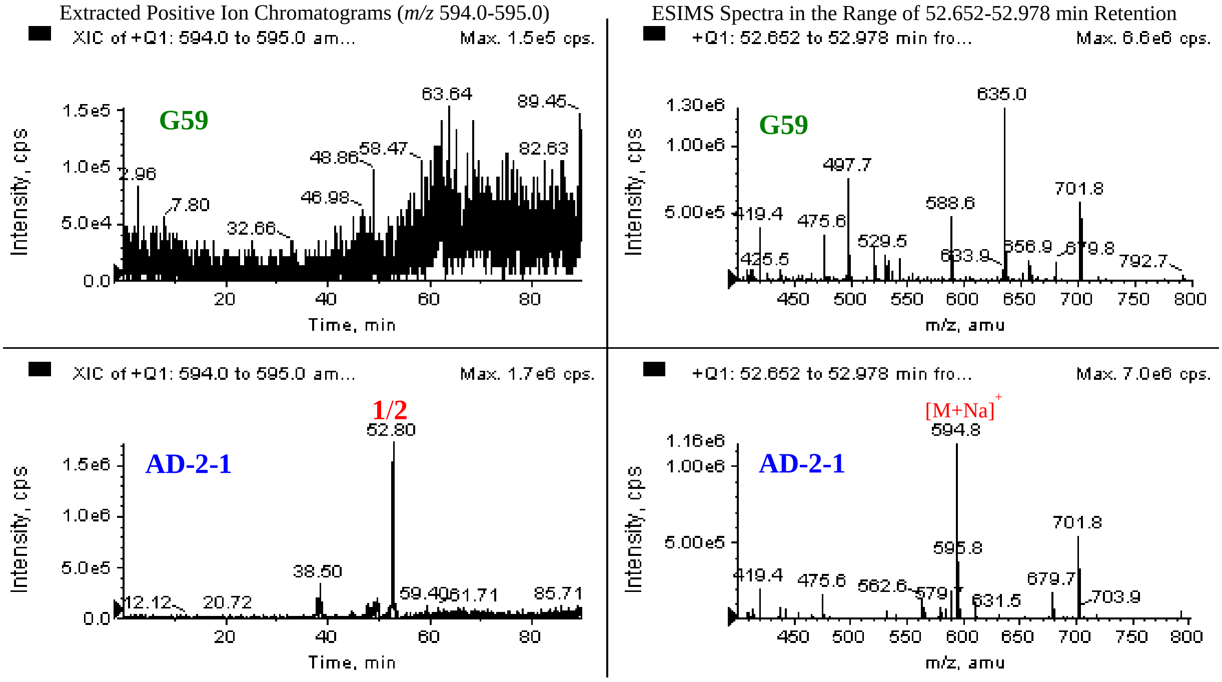

B: HPLC-Negative ion ESI-MS analysis (ESIMS *m/z*: 556 [M-H]$^{-}$ and 592 [M+Cl]$^{-}$ for both **3** and **4**)

Extracted Negative Ion Chromatograms (*m/z* 591.5-593.0) ESIMS Spectra in the Range of 51.1-51.4 min Retention Times

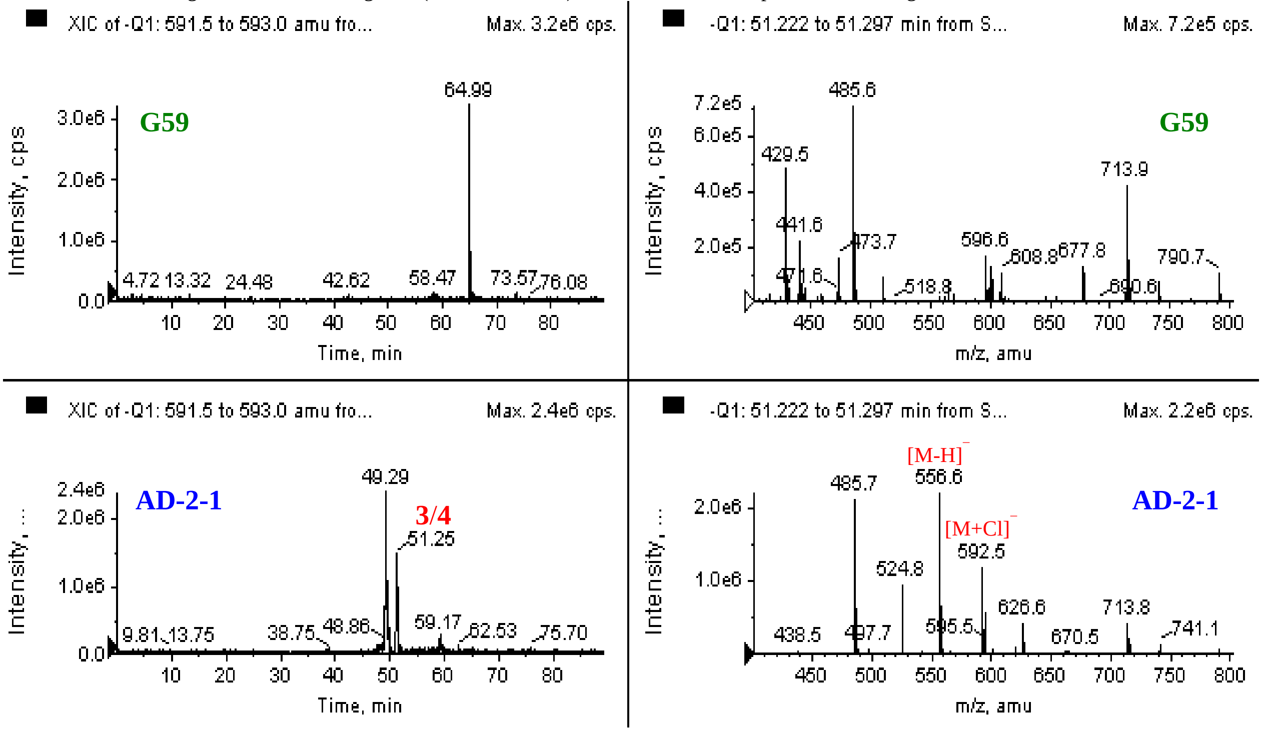

Figure S2. *Cont.*

C: HPLC-Negative ion ESI-MS analysis (ESIMS *m/z*: 569 [M-H]⁻ for 5/6)

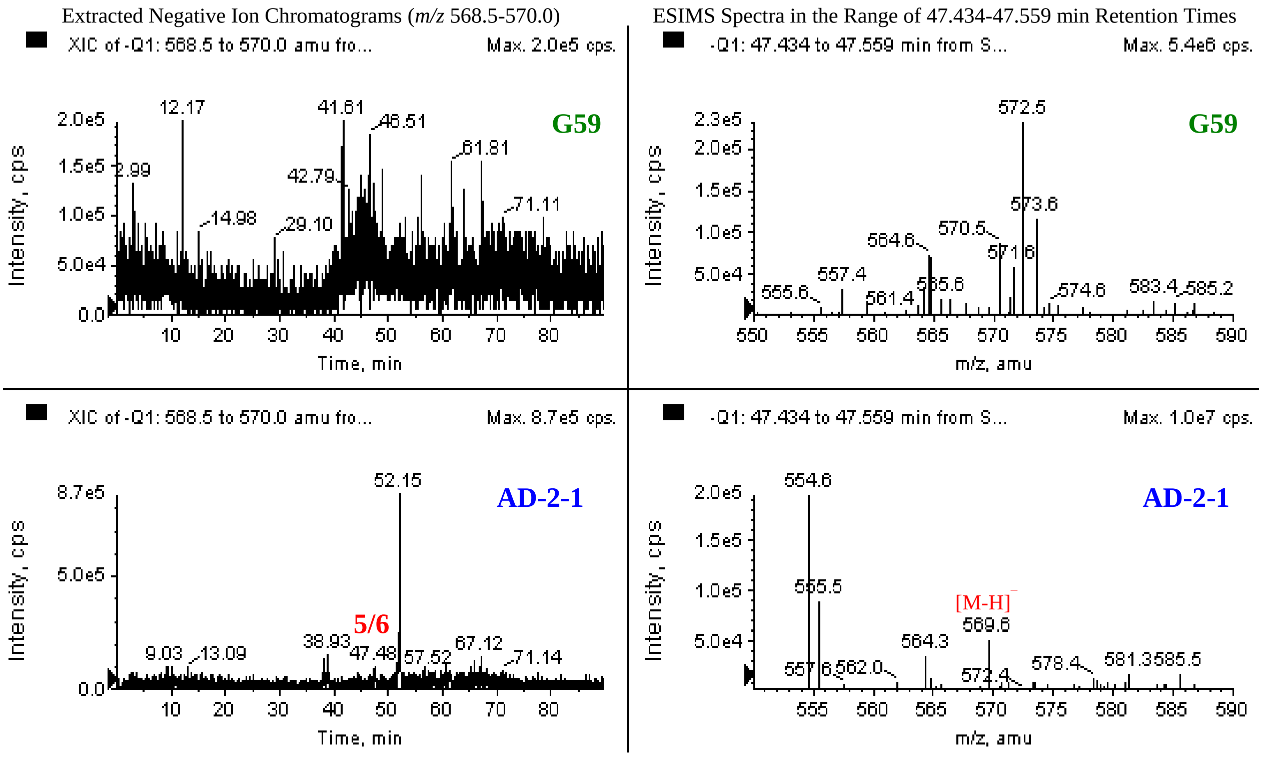

D: HPLC-Negative ion ESI-MS analysis (ESIMS *m/z*: 457 [M-H]⁻ for 7)

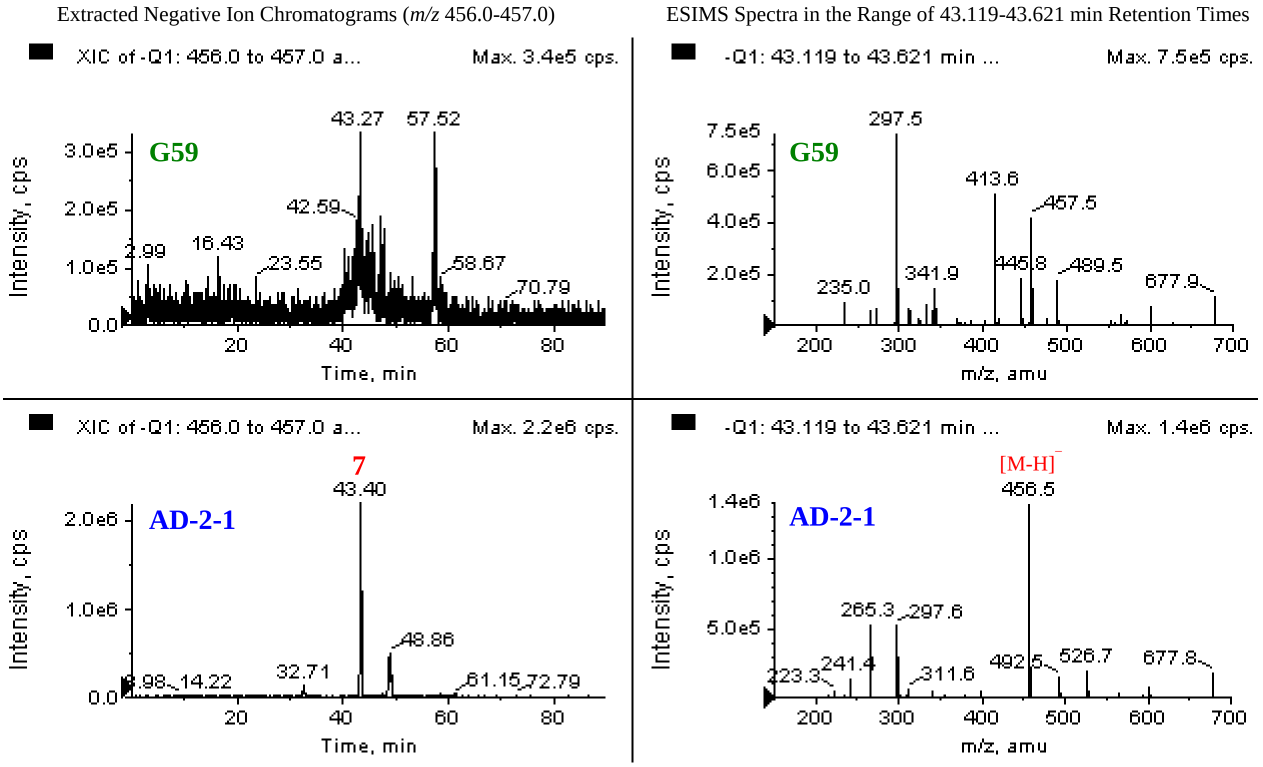

Figure S2. *Cont.*

E: HPLC-Negative ion ESI-MS analysis (ESIMS *m/z*: 454 [M-H]⁻ for **8**)

Extracted Negative Ion Chromatograms (*m/z* 554.0-555.0) ESIMS Spectra in the Range of 49.265-49.967min Retention Times

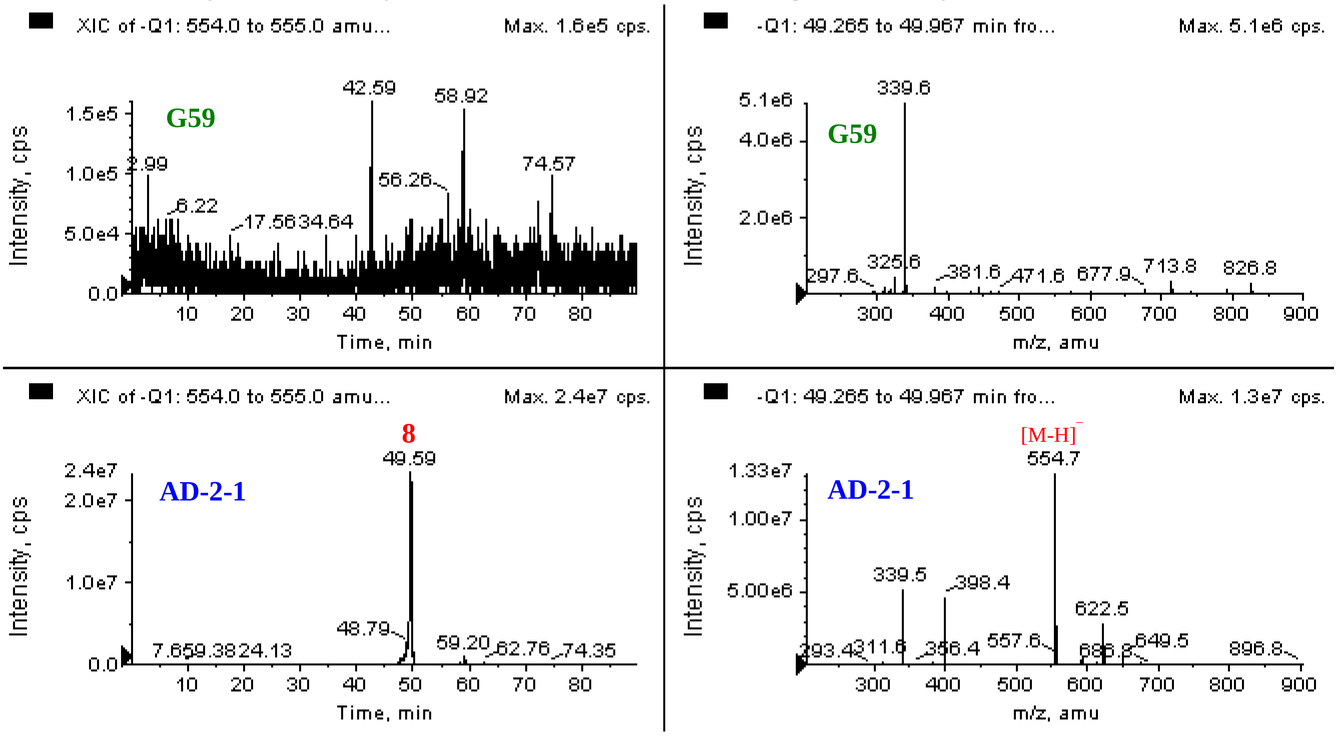

F: HPLC-Negative ion ESI-MS analysis (ESIMS *m/z*: 456 [M-H]⁻ and 592 [M+Cl]⁻ for **9**)

Extracted Negative Ion Chromatograms (*m/z* 592.0-593.0) ESIMS Spectra in the Range of 49.240-49.340 min Retention Times

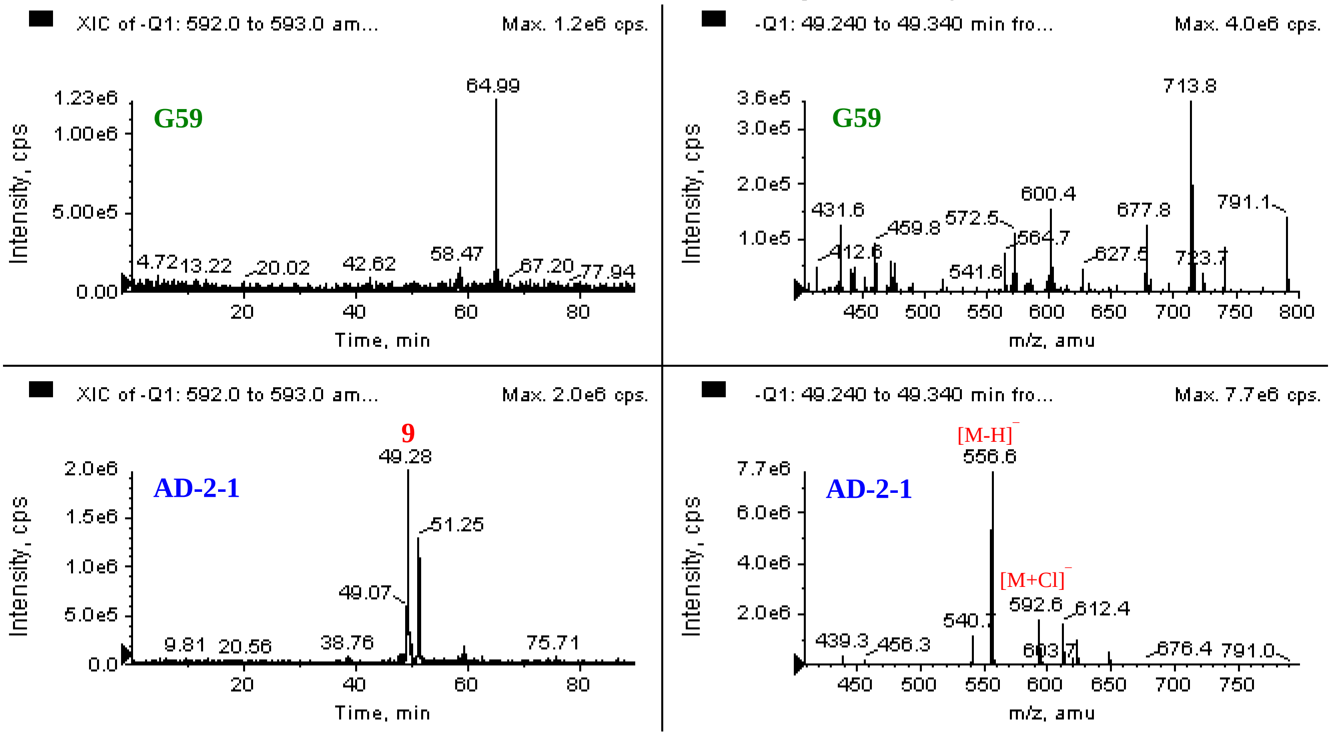

Figure S2. *Cont.*

G: HPLC-Negative ion ESI-MS analysis (ESIMS *m/z*: 385 [M-H]$^-$ for **10**)

Extracted Negative Ion Chromatograms (*m/z* 385.0-386.0) ESIMS Spectra in the Range of 62.334-62.434 min Retention Times

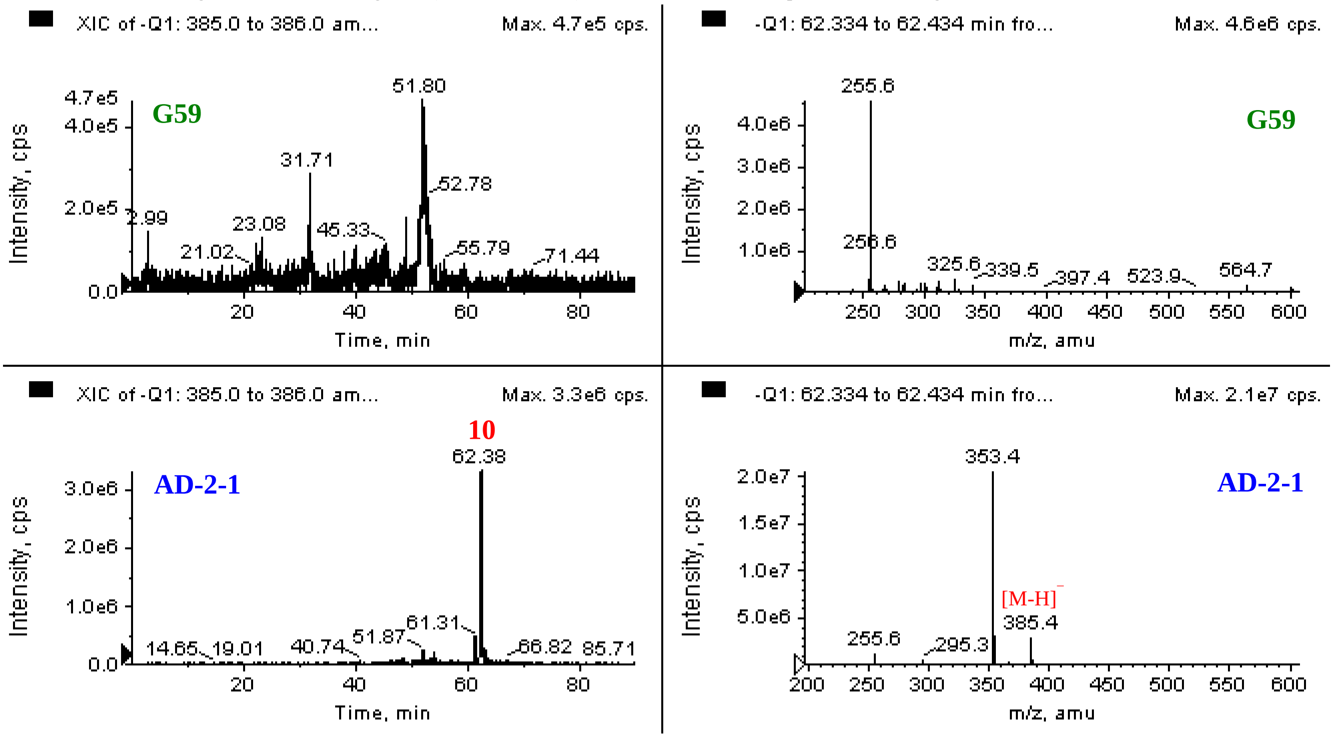

H: HPLC-Negative ion ESI-MS analysis (ESIMS *m/z*: 371 [M-H]$^-$ for **11**)

Extracted Negative Ion Chromatograms (*m/z* 371.0-372.0) ESIMS Spectra in the Range of 58.822-58.922 min Retention Times

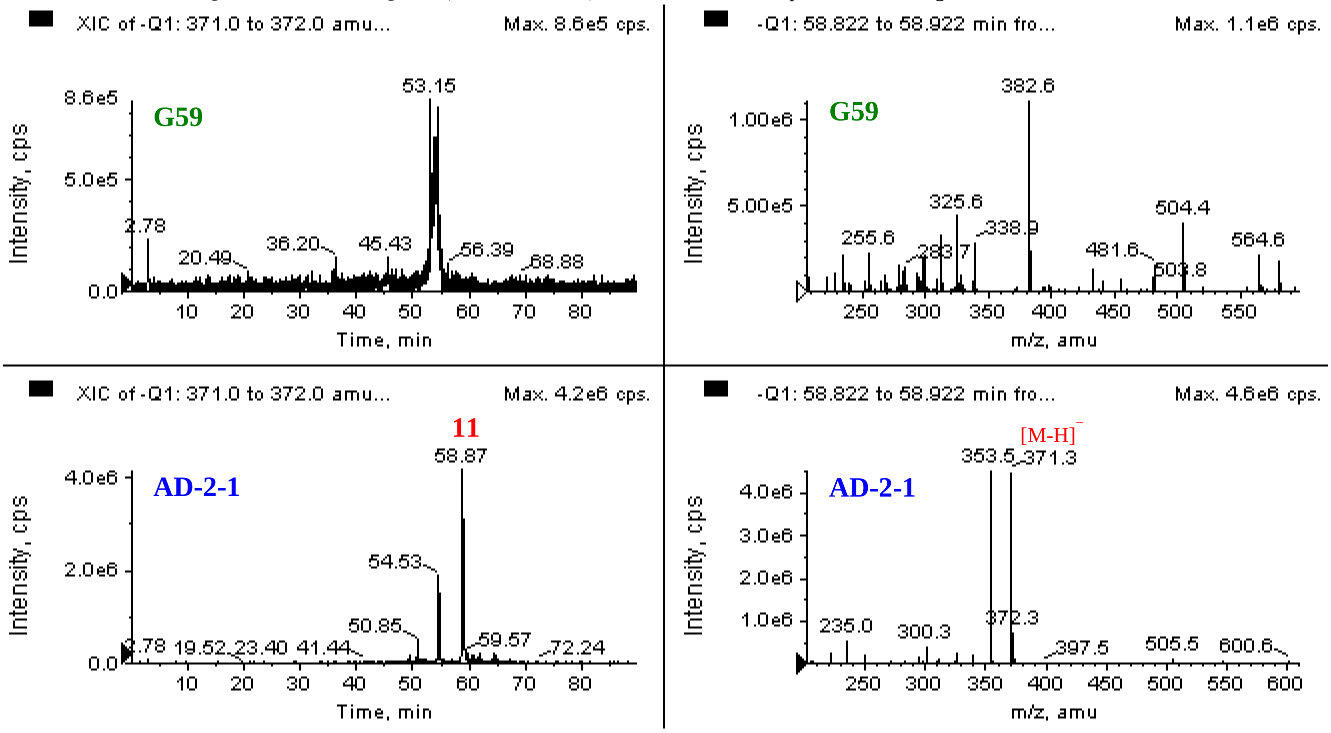

Figure S2. *Cont.*

I: HPLC-Negative ion ESI-MS analysis (ESIMS *m/z*: 367 [M-H]⁻ for **12**)

Extracted Negative Ion Chromatograms (*m/z* 367.0-368.0) ESIMS Spectra in the Range of 61.5-62.1 min Retention Times

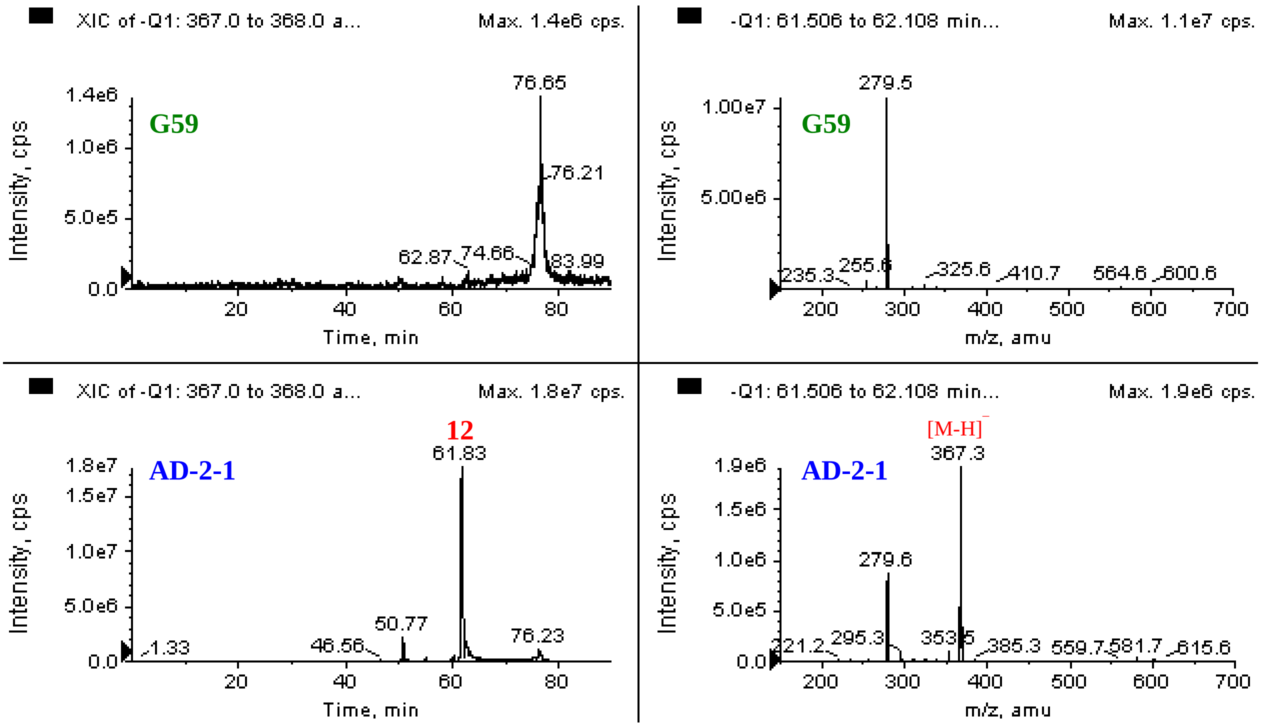

J: HPLC-Negative ion ESI-MS analysis (ESIMS *m/z*: 383 [M-H]⁻ for **13**)

Extracted Negative Ion Chromatograms (*m/z* 383.0-384.0) ESIMS Spectra in the Range of 51.798-52.400 min Retention Times

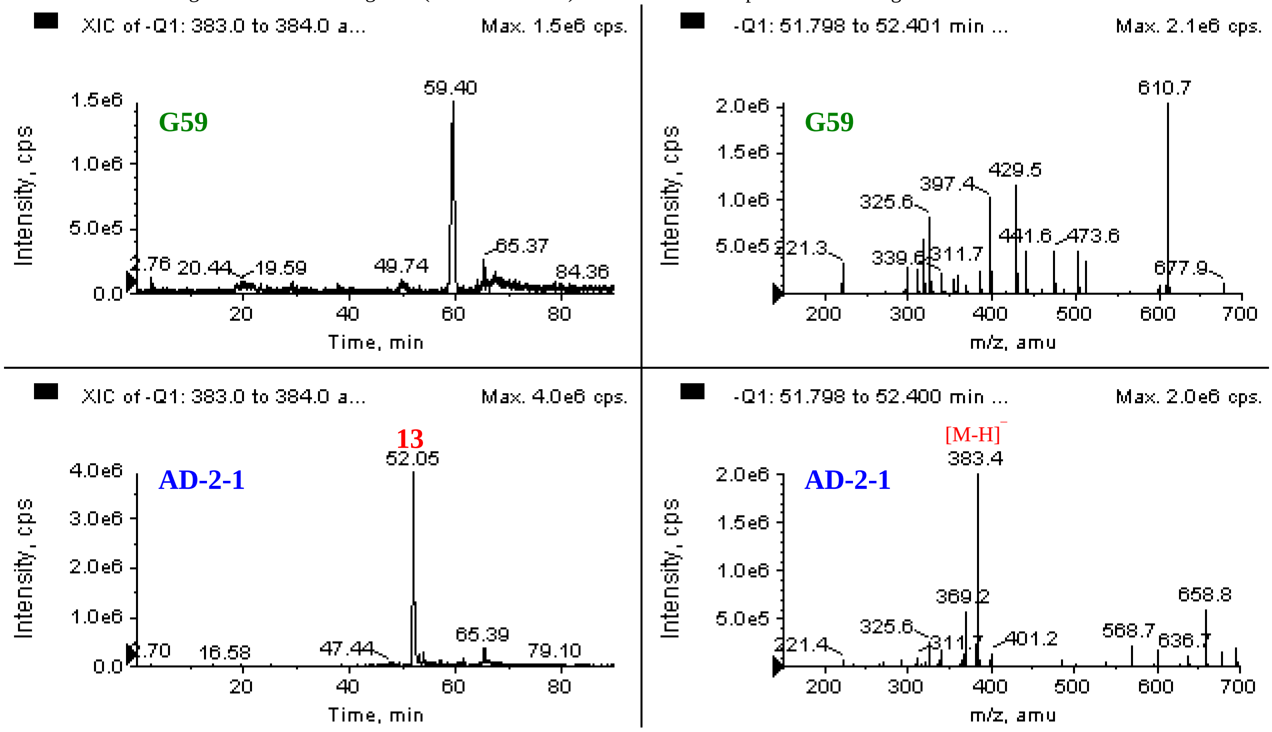

Figure S2. *Cont.*

K: HPLC-Positive ion ESI-MS analysis (ESIMS *m/z*: 347 $[M+Na]^+$ for **14**)

Extracted Positive Ion Chromatograms (*m/z* 347.0-348.0) ESIMS Spectra in the Range of 47.459-48.086 min Retention Times

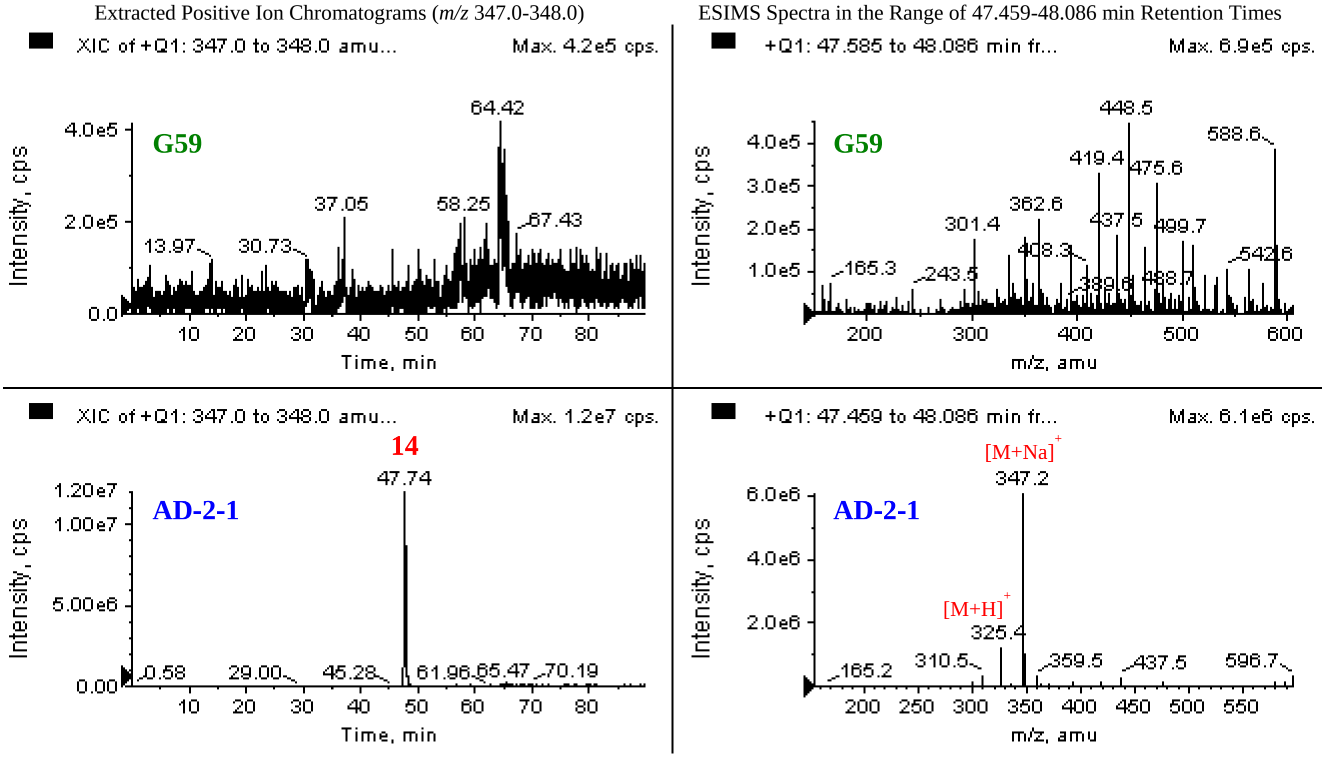

Appendix of Spectra

Figure SP1. A: Positive (A) and negative (B) ESIMS spectra of **1**.

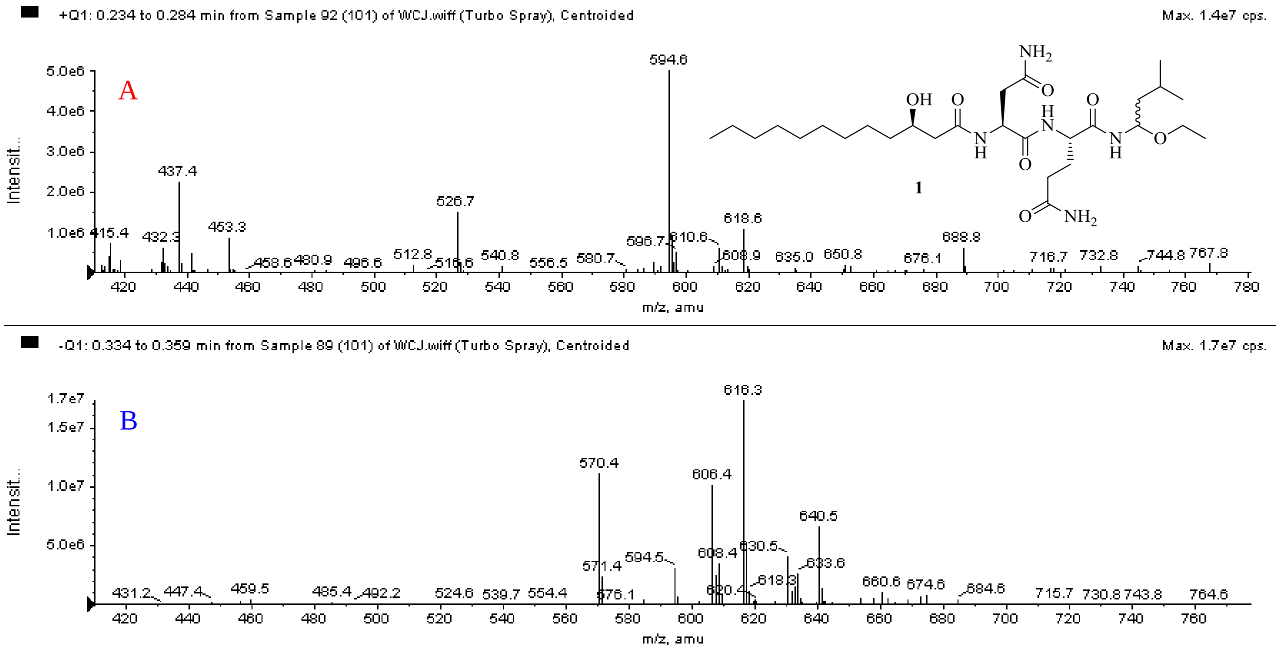

Figure SP1. B: Positive HRESIMS spectrum of **1**.

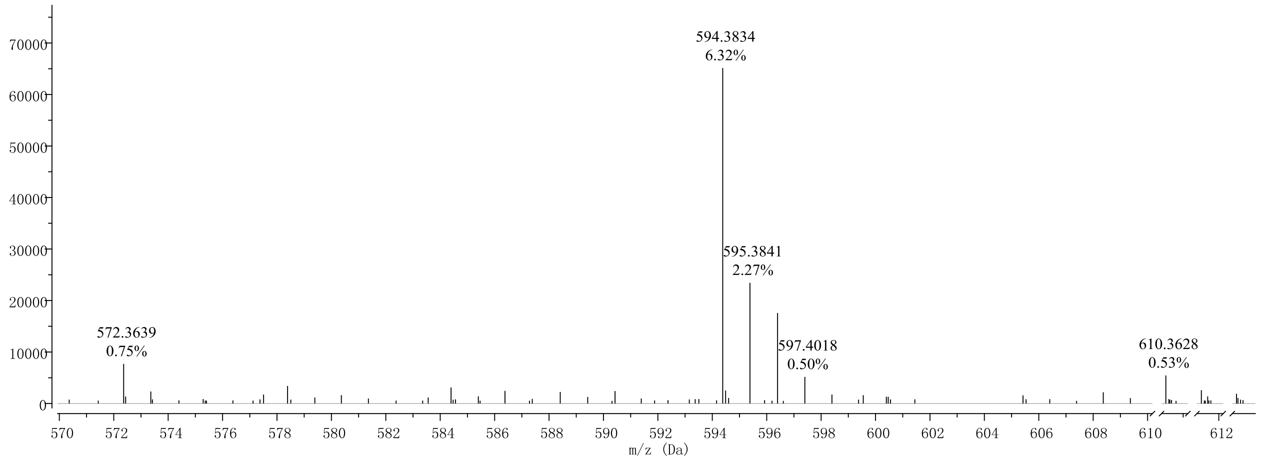

Figure SP1. C: IR spectrum of **1**.

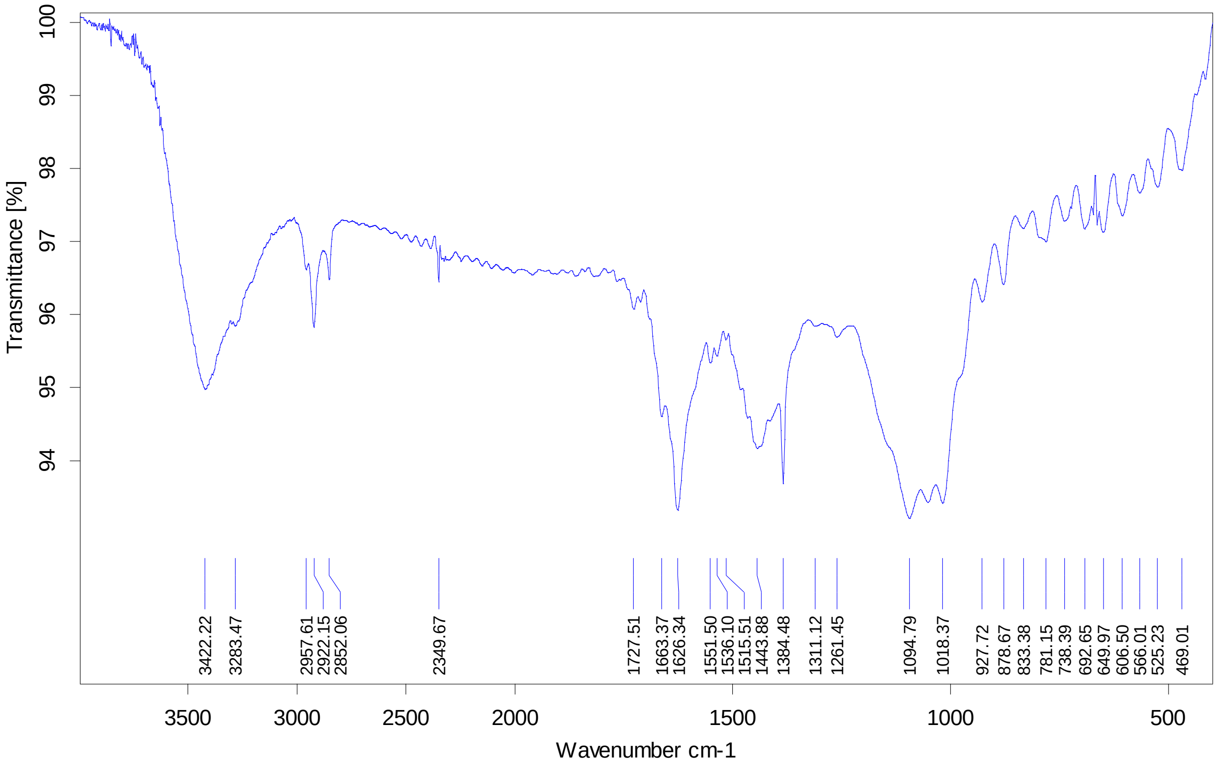

Figure SP1. D: 600 MHz ^{1}H NMR spectrum of **1** in DMSO-d_6.

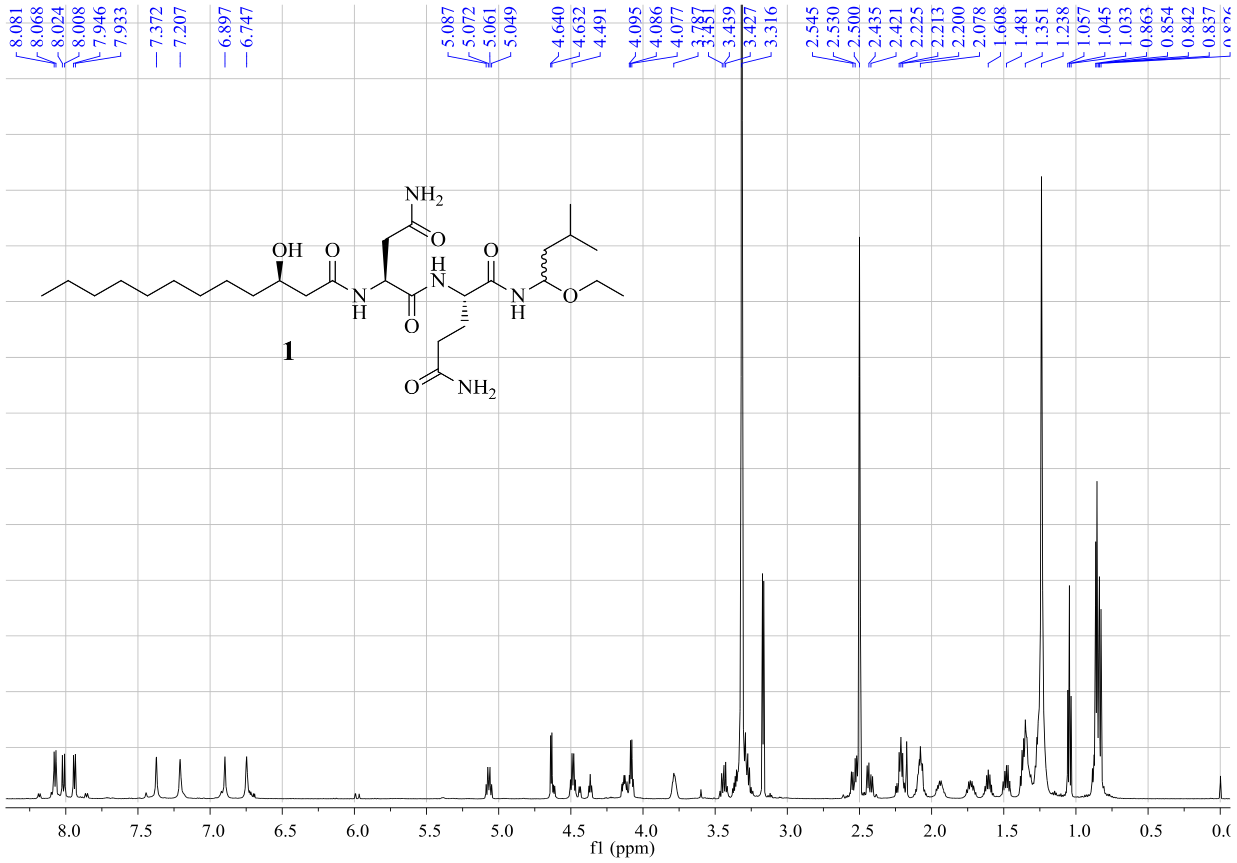

Figure SP1. E: 150 MHz ^{13}C NMR spectrum of **1** in DMSO-d_6.

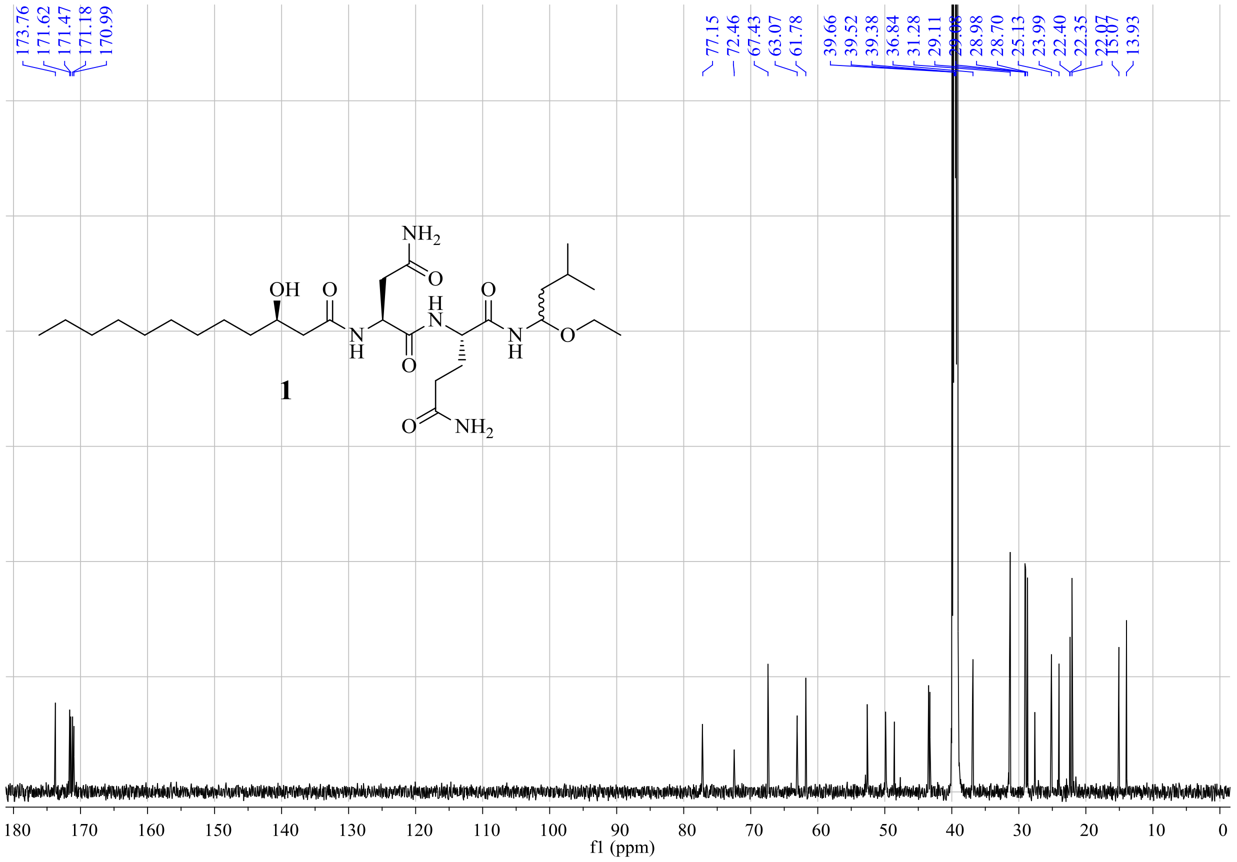

Figure SP1. F: DEPT spectra of **1** in DMSO-d_6.

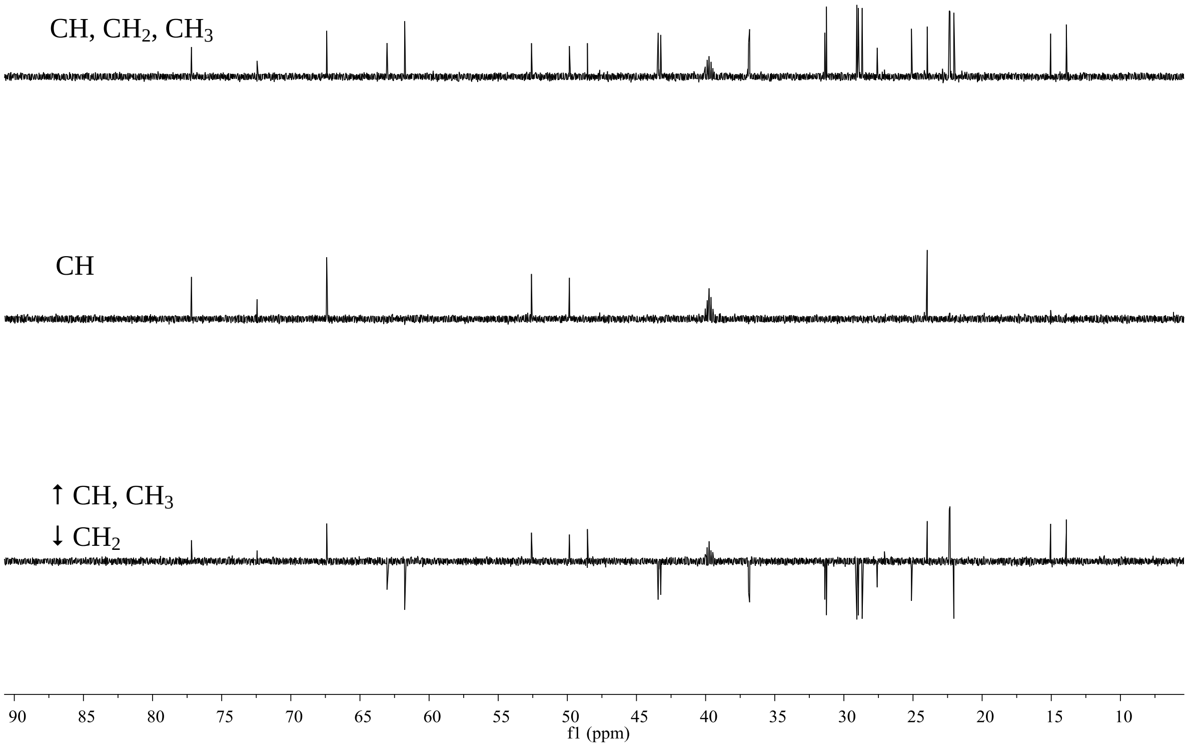

Figure SP1. G: ^{1}H-^{1}H COSY spectrum of **1** in DMSO-d_6.

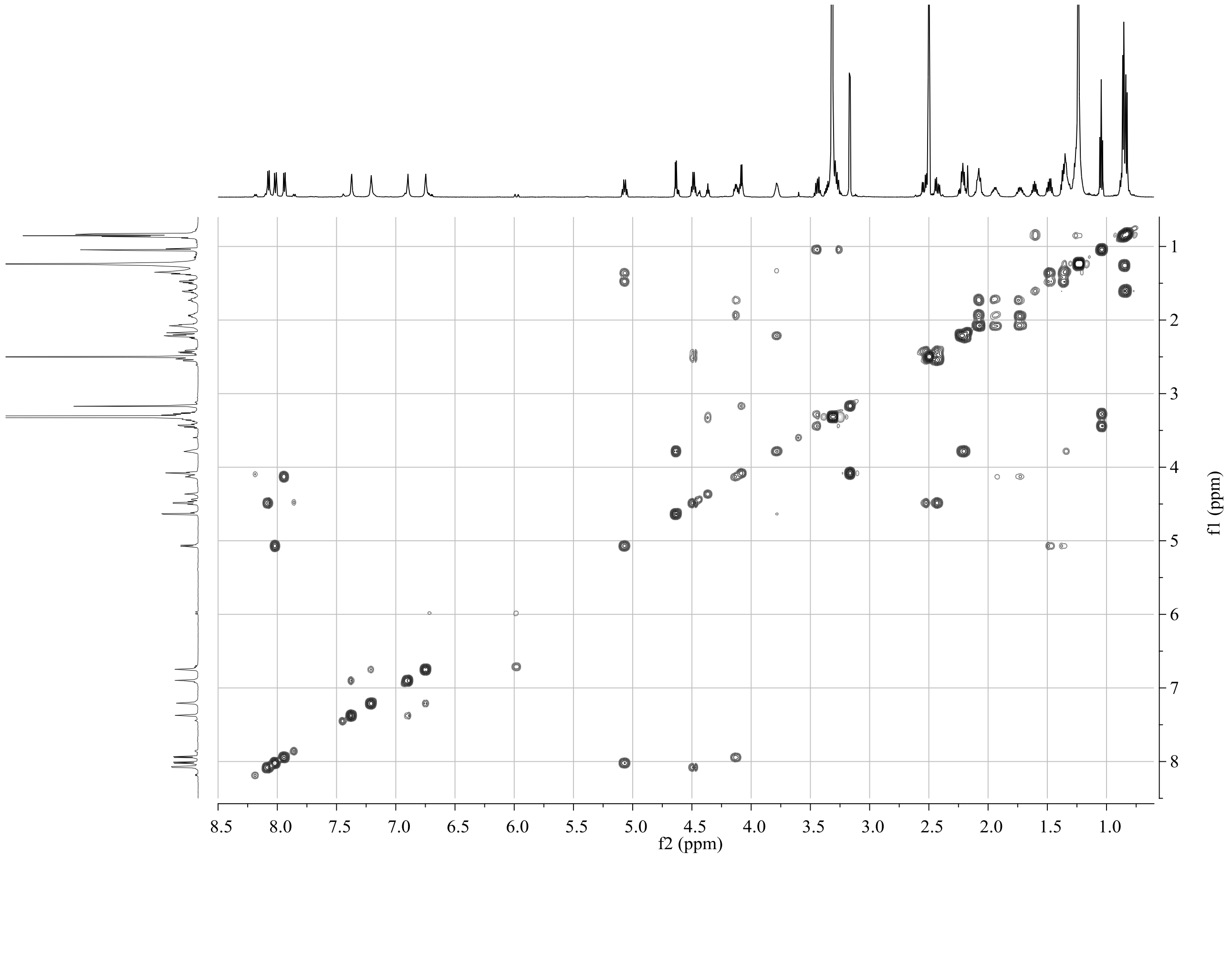

Figure SP1. H: HMQC spectrum of **1** in DMSO-d_6.

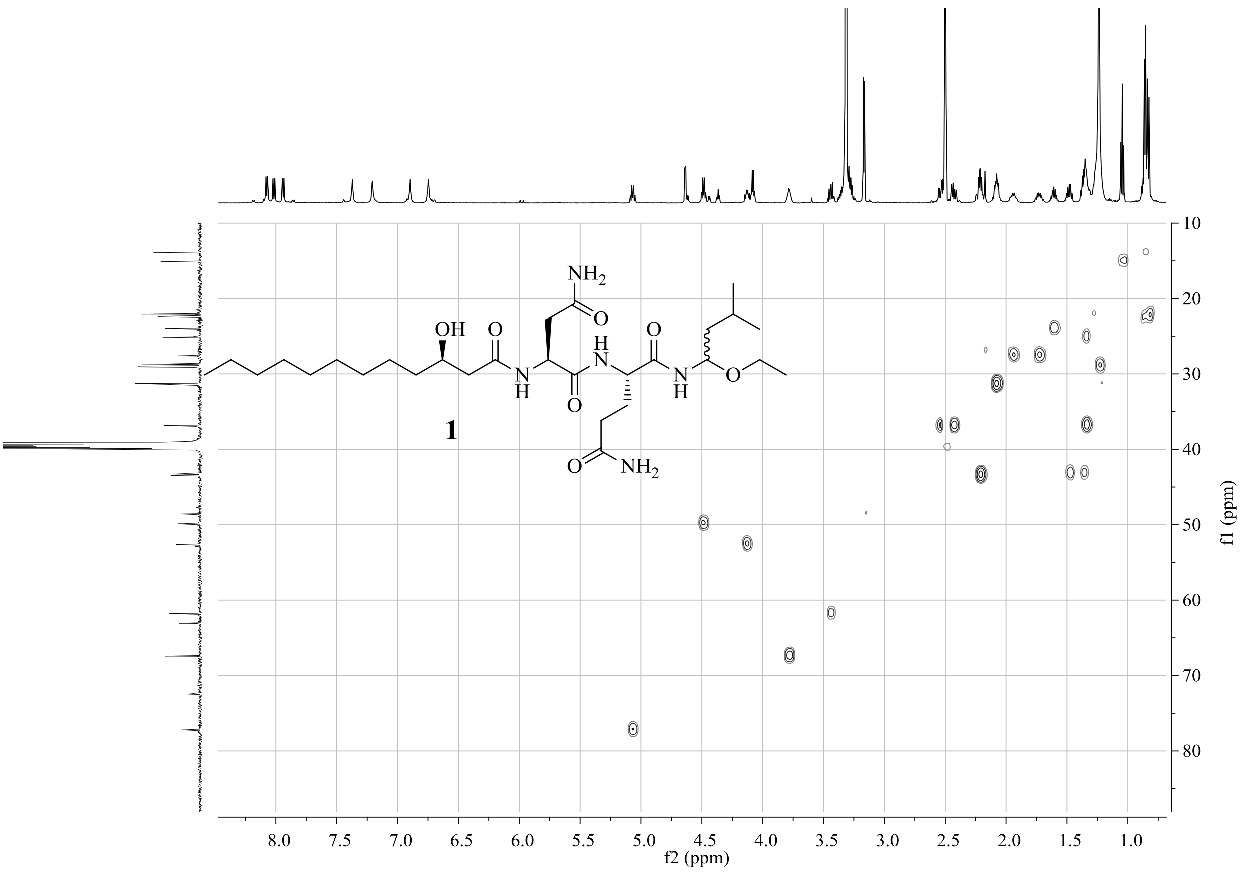

Figure SP1. I: HMBC spectrum of **1** in DMSO-d_6.

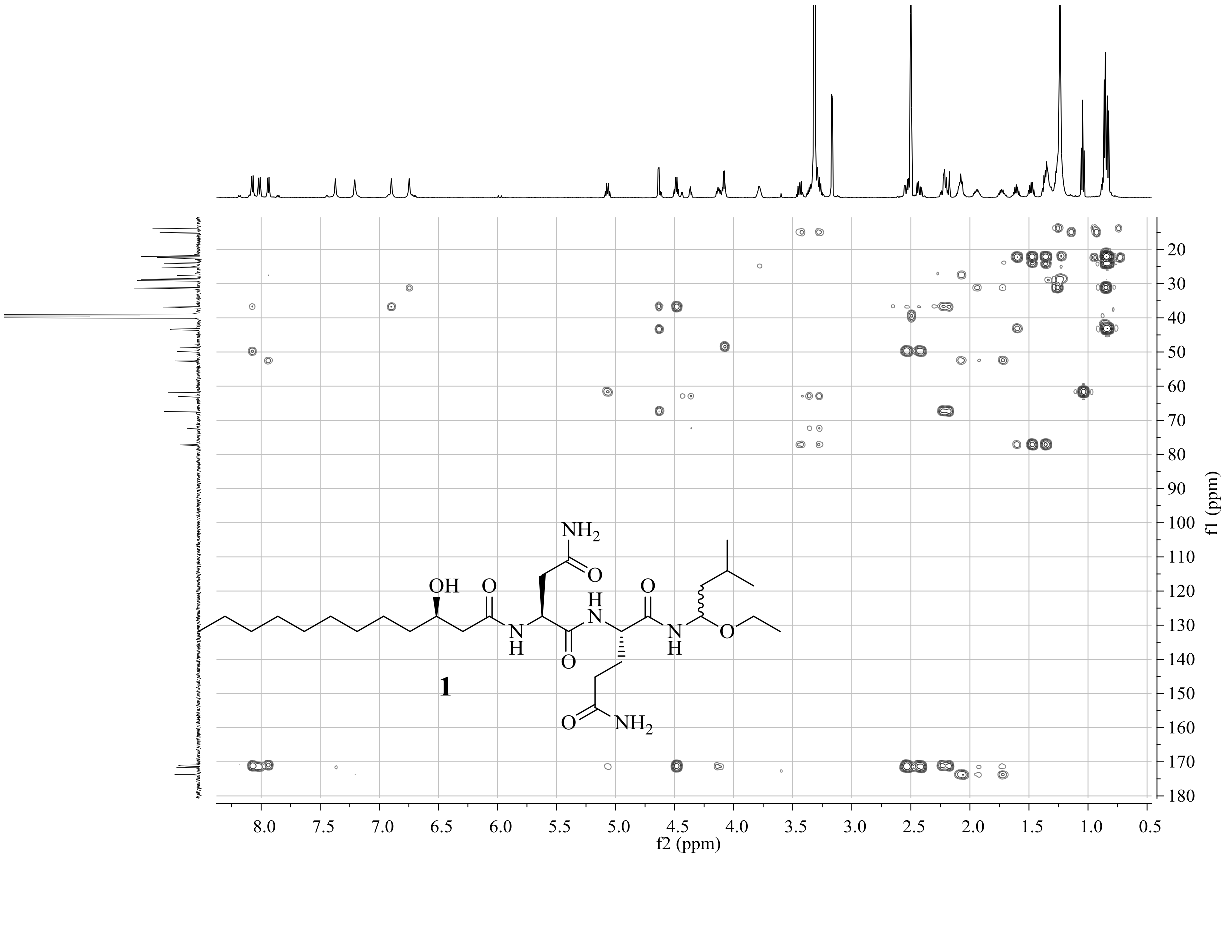

Figure SP2. A: Positive (A) and negative (B) ESIMS spectra of **2**.

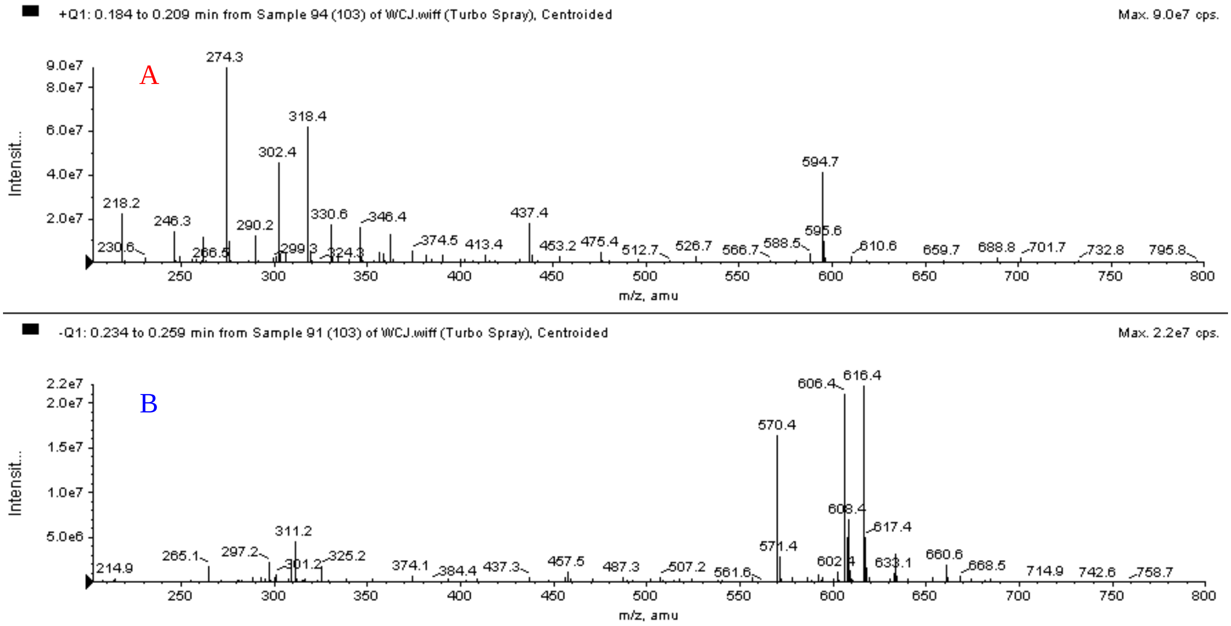

Figure SP2. B: Positive HRESIMS spectrum of **2**.

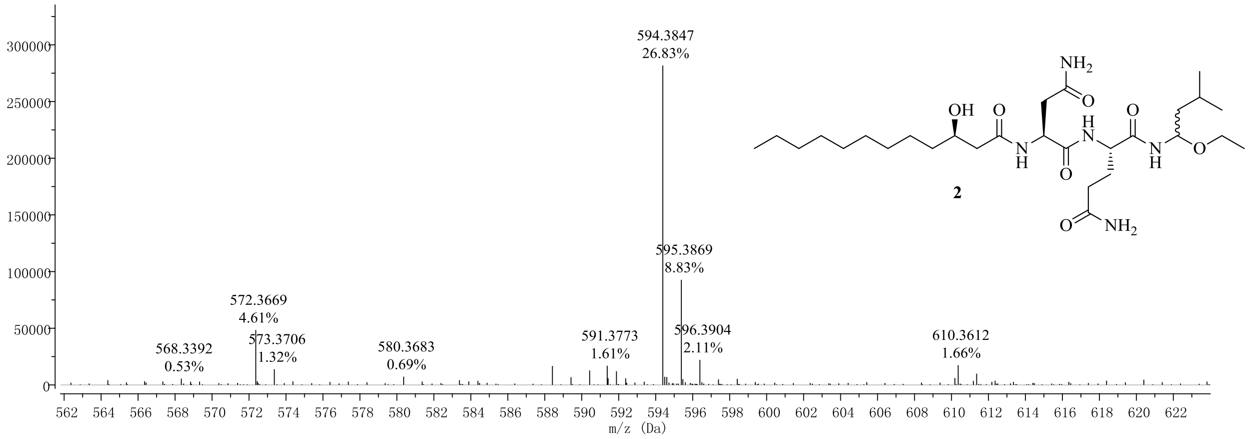

Figure SP2. C: IR spectrum of **2**.

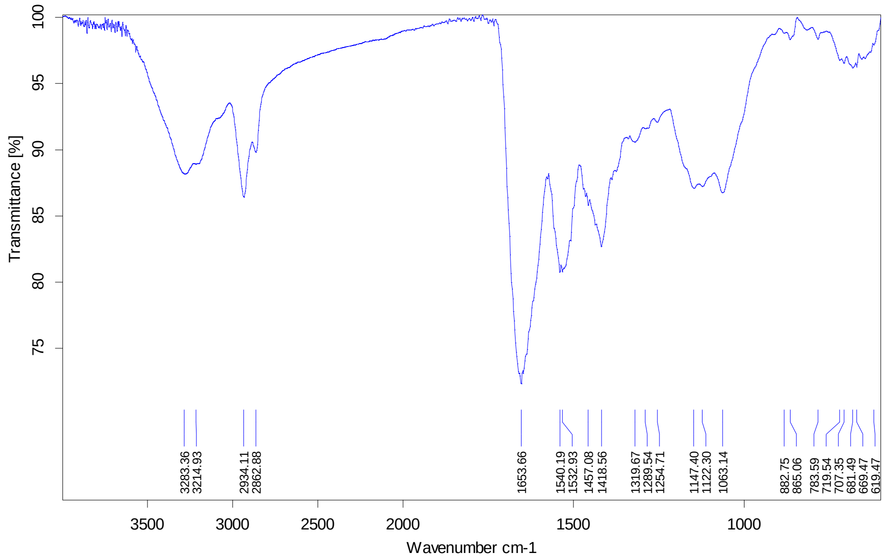

Figure SP2. D: 600 MHz ^{1}H NMR spectrum of **2** in DMSO-d_6.

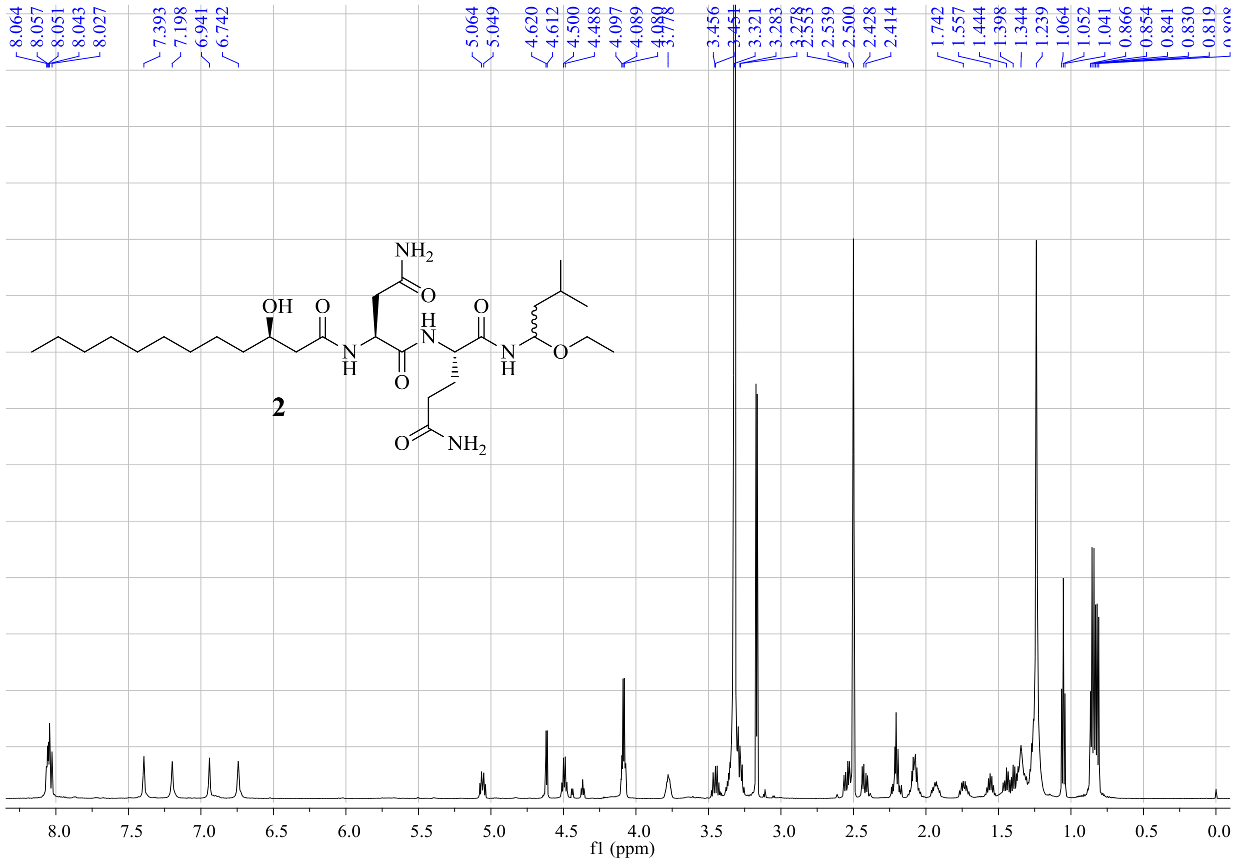

Figure SP2. E: 150 MHz ^{13}C NMR spectrum of **2** in DMSO-d_6.

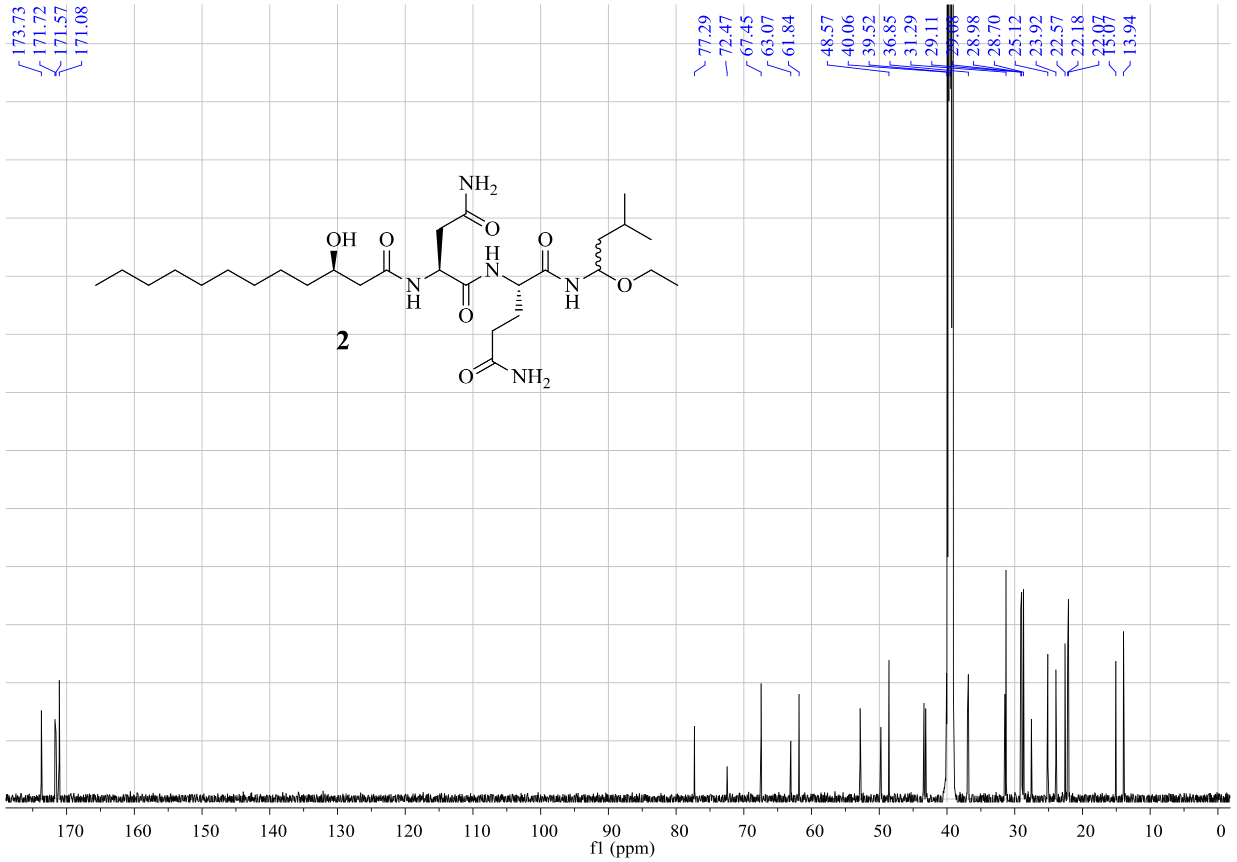

Figure SP2. F: DEPT spectra of **2** in DMSO-d_6.

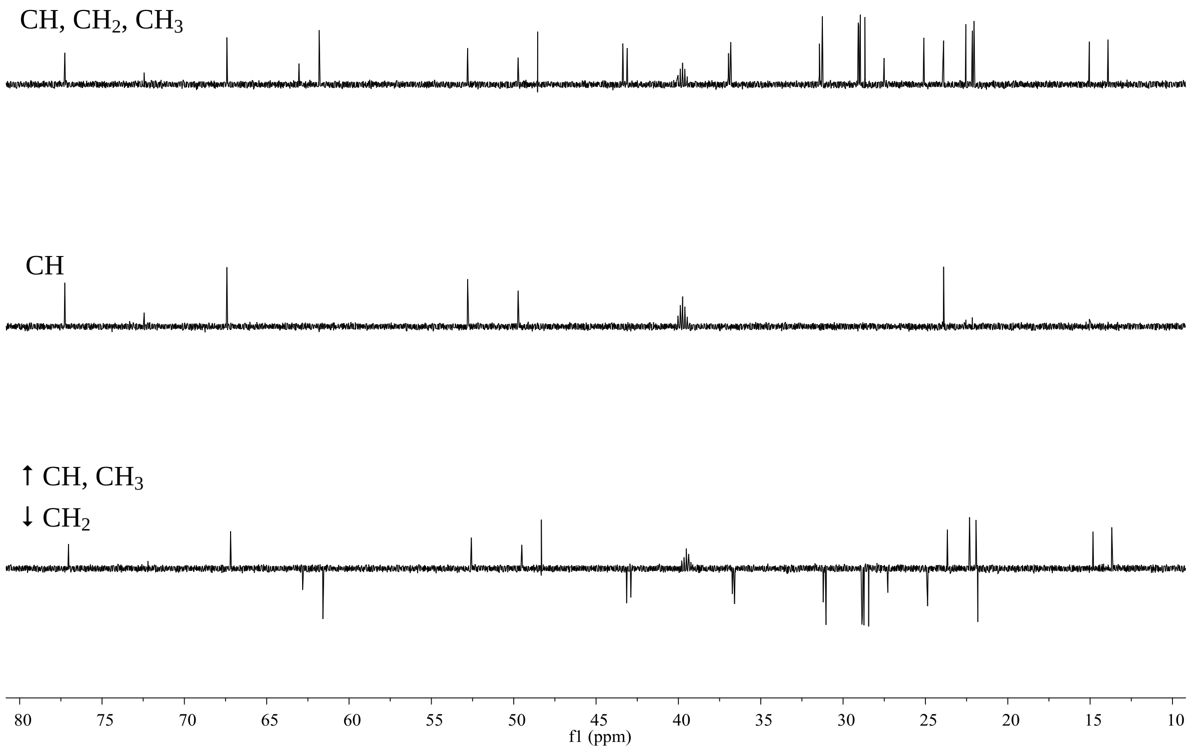

Figure SP2. G: ^{1}H-^{1}H COSY spectrum of **2** in DMSO-d_6.

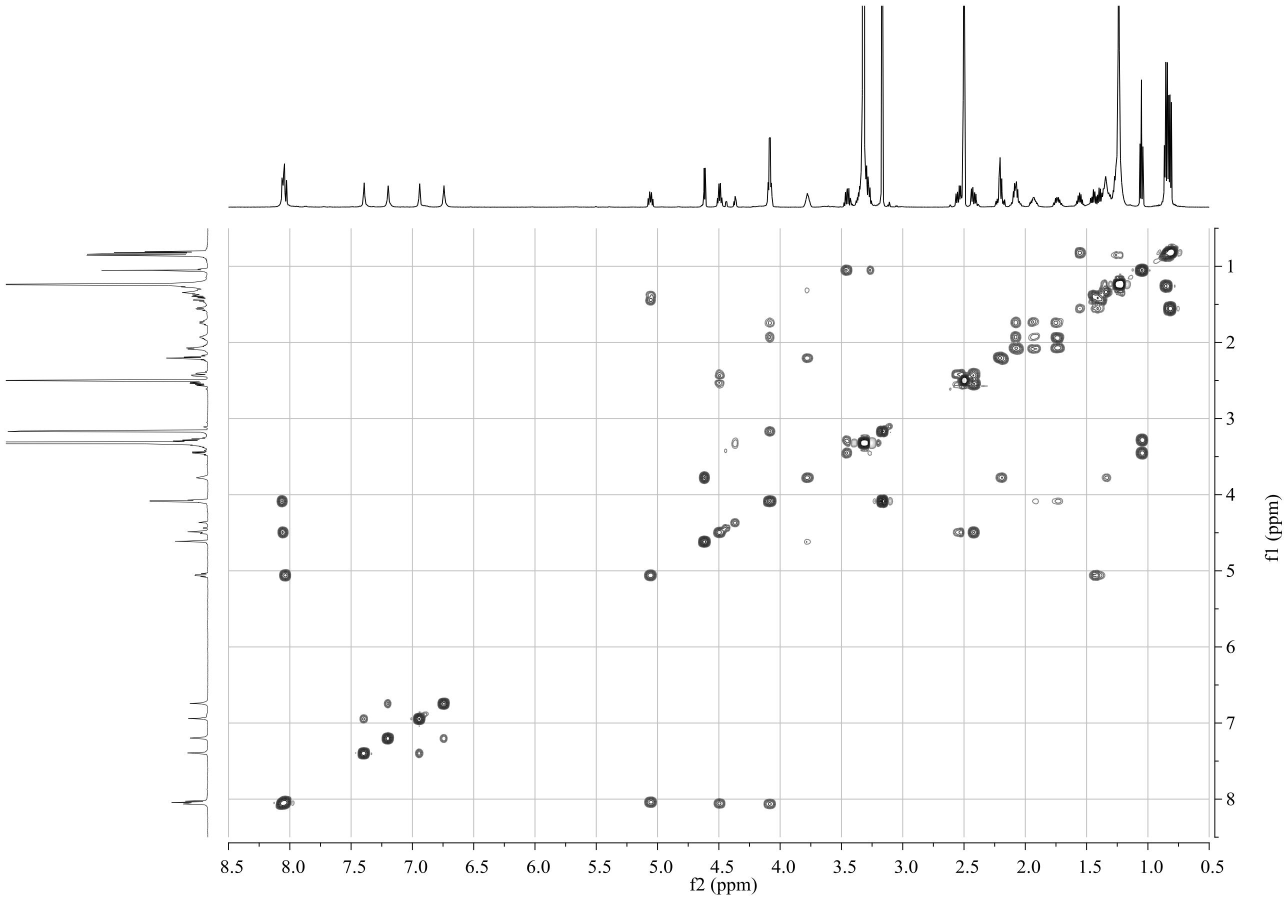

Figure SP2. H: HMQC spectrum of **2** in DMSO-d_6.

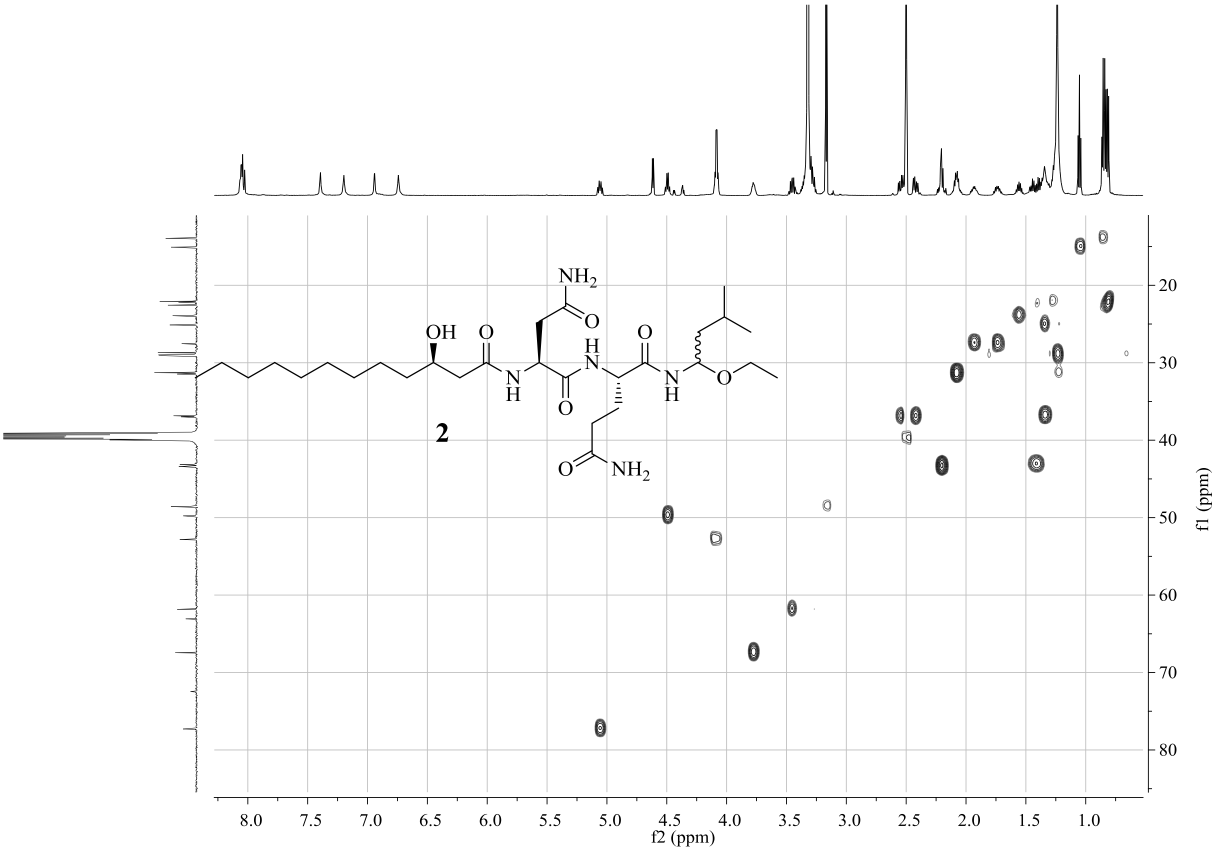

Figure SP2. I: HMBC spectrum of **2** in DMSO-d_6.

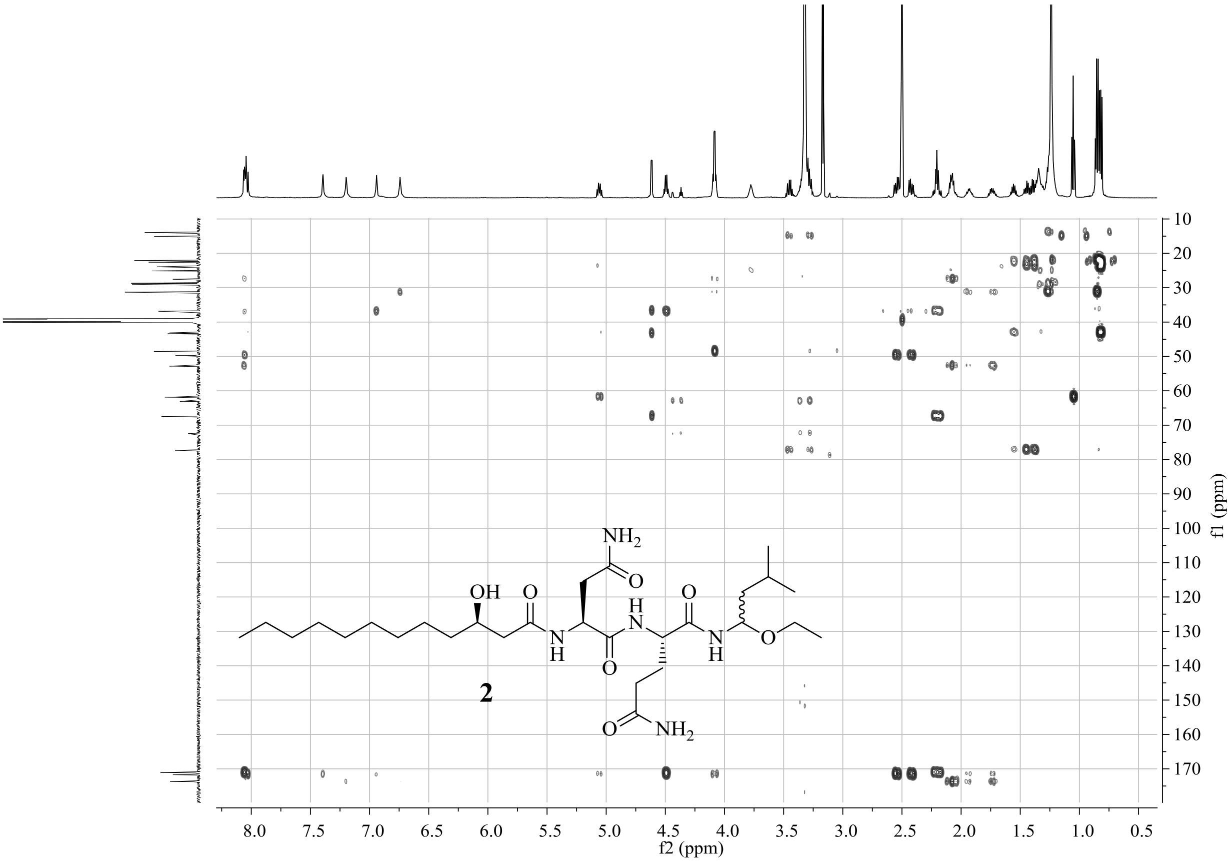

Figure SP3. A: Positive (A) and negative (B) ESIMS spectra of **3**.

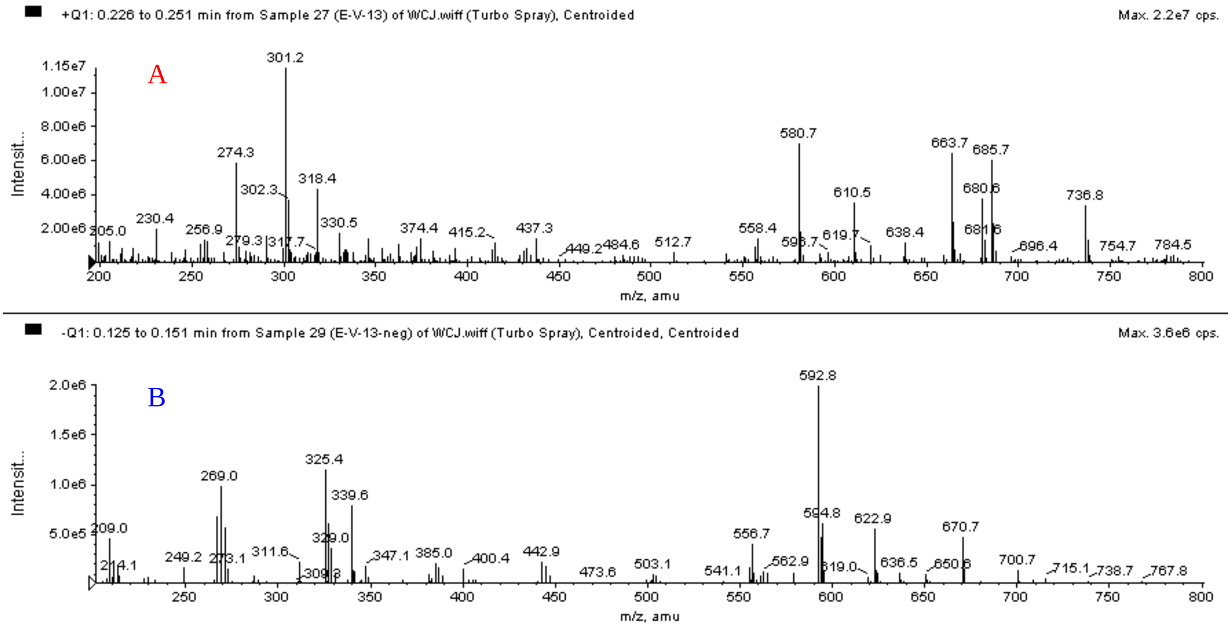

Figure SP3. B: Positive HRESIMS spectrum of **3**.

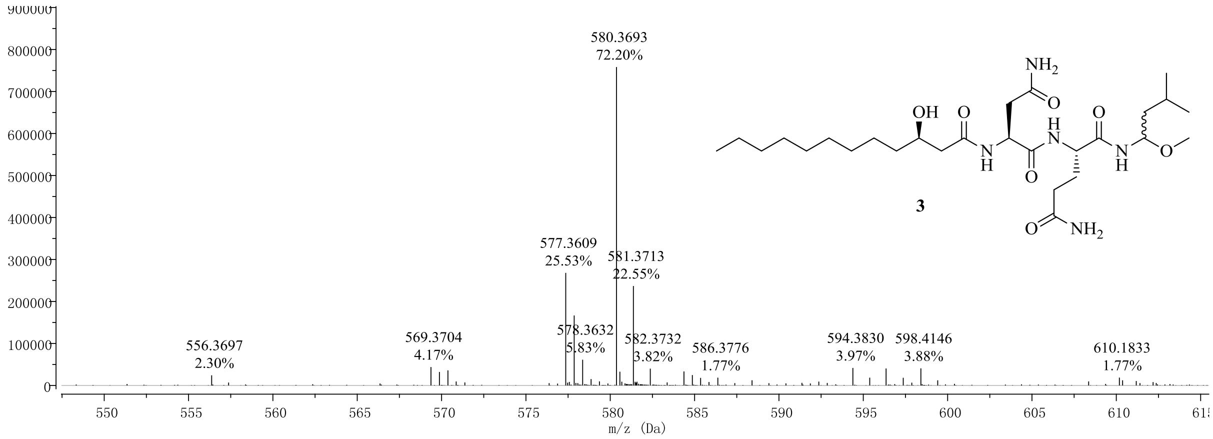

Figure SP3. C: IR spectrum of 3.

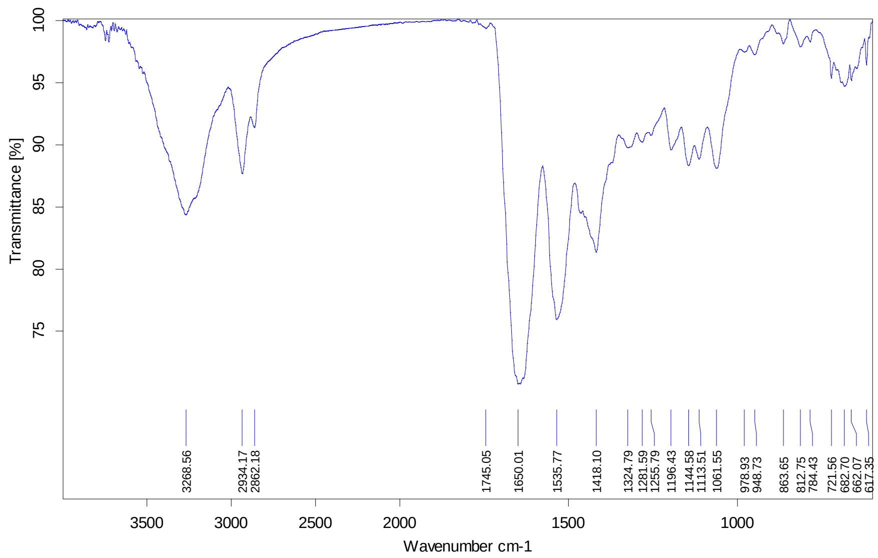

Figure SP3. D: 400 MHz ^{1}H NMR spectrum of **3** in DMSO-d_6.

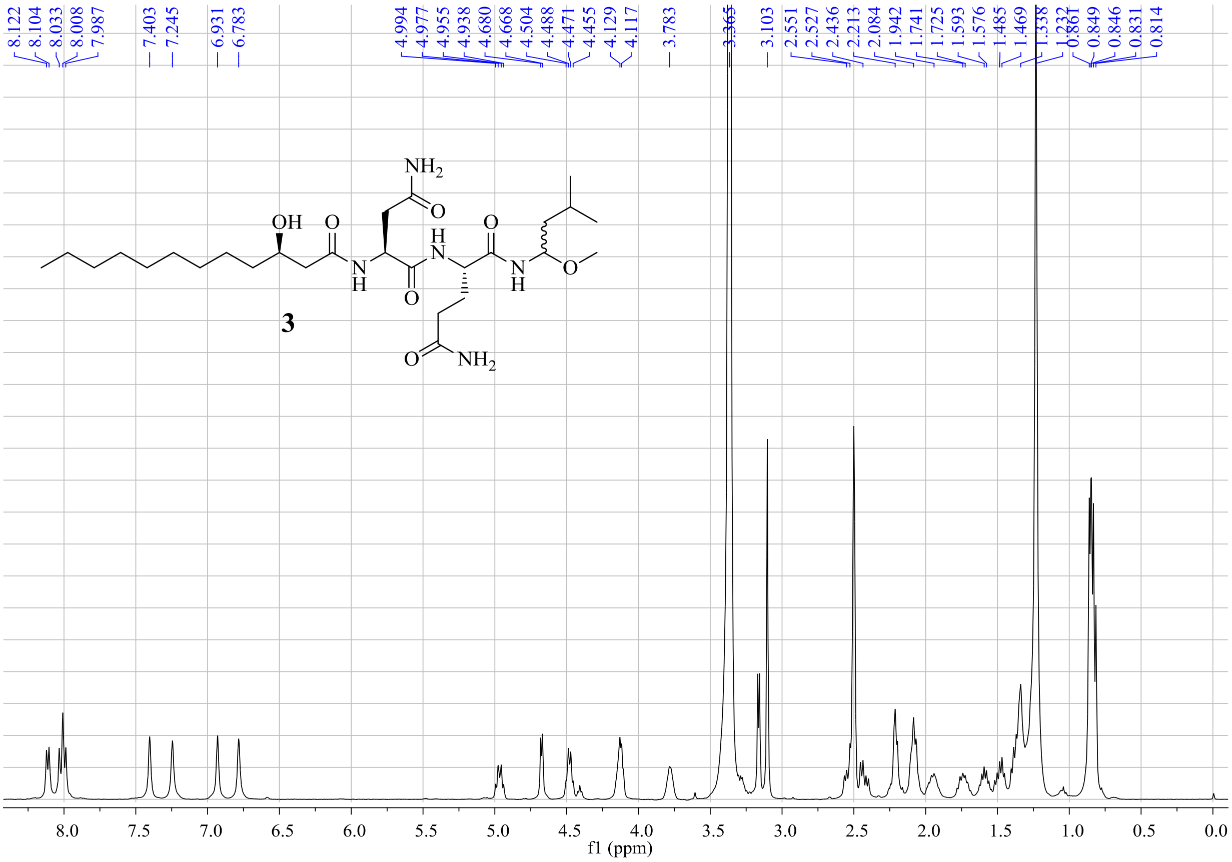

Figure SP3. E: 100 MHz ^{13}C NMR spectrum of **3** in DMSO-d_6.

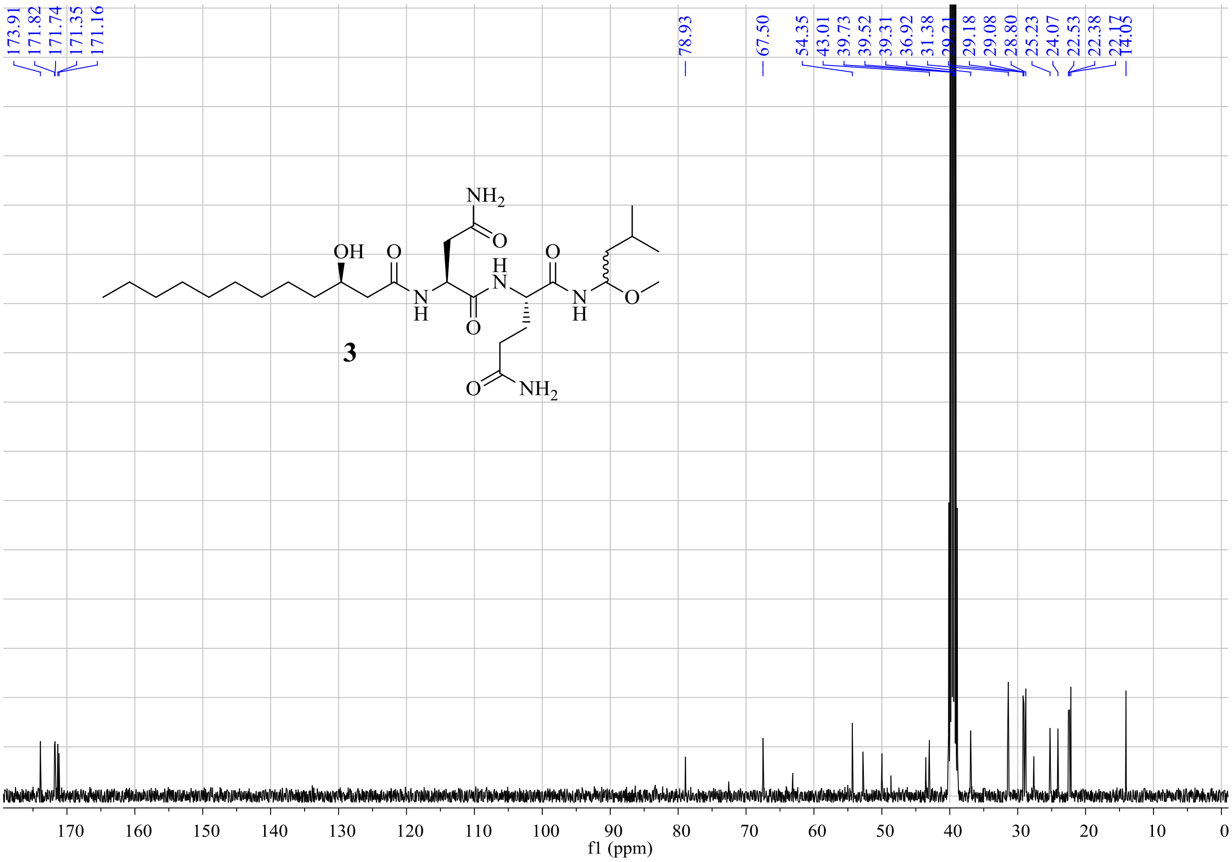

Figure SP3. F: DEPT spectra of **3** in DMSO-d_6.

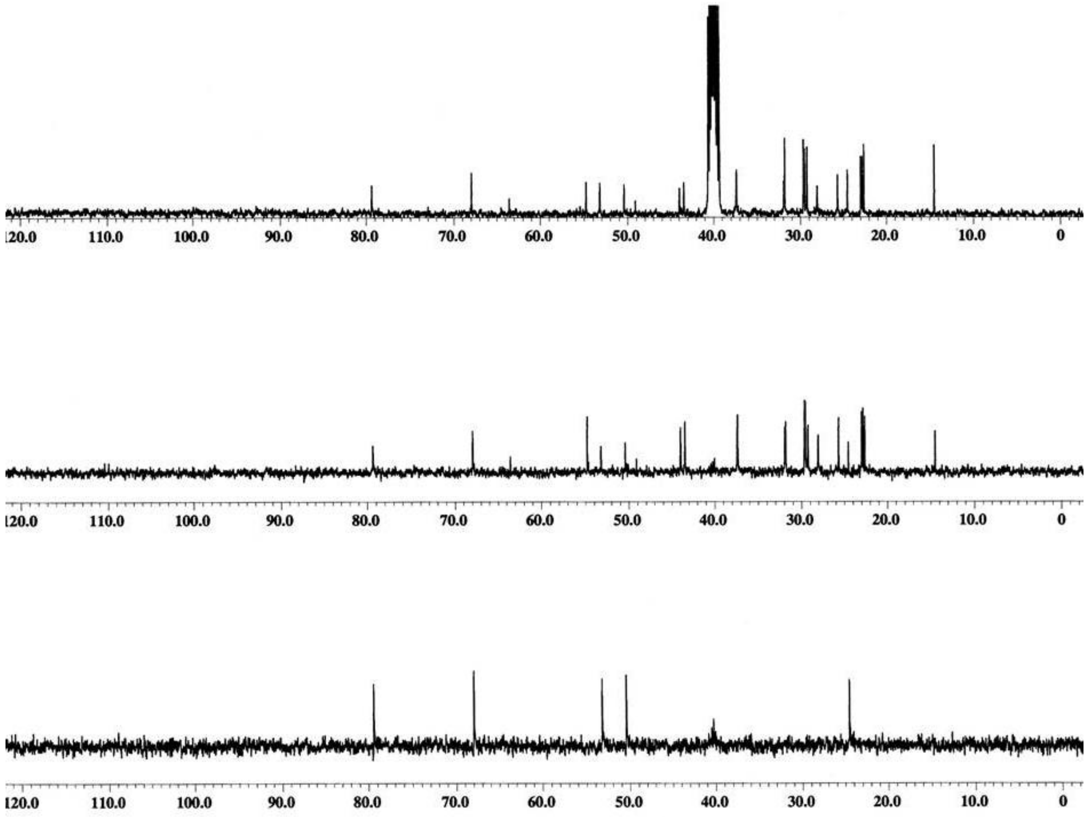

Figure SP3. G: ^{1}H-^{1}H COSY spectrum of **3** in DMSO-d_6.

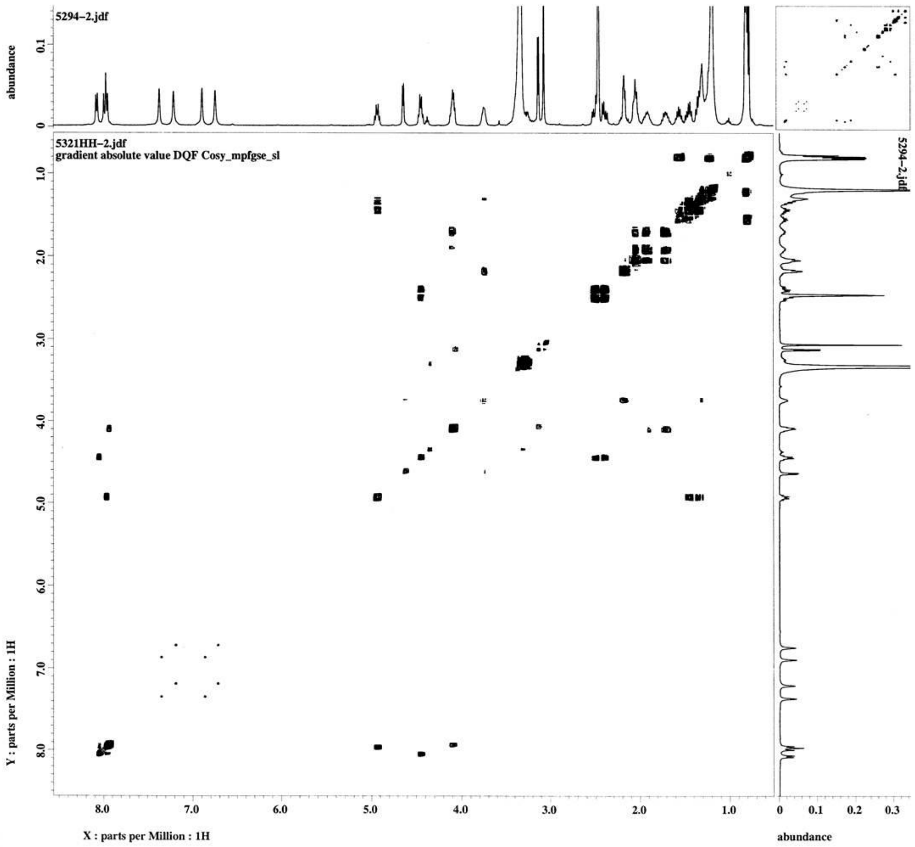

Figure SP3. H: HMQC spectrum of **3** in DMSO-d_6.

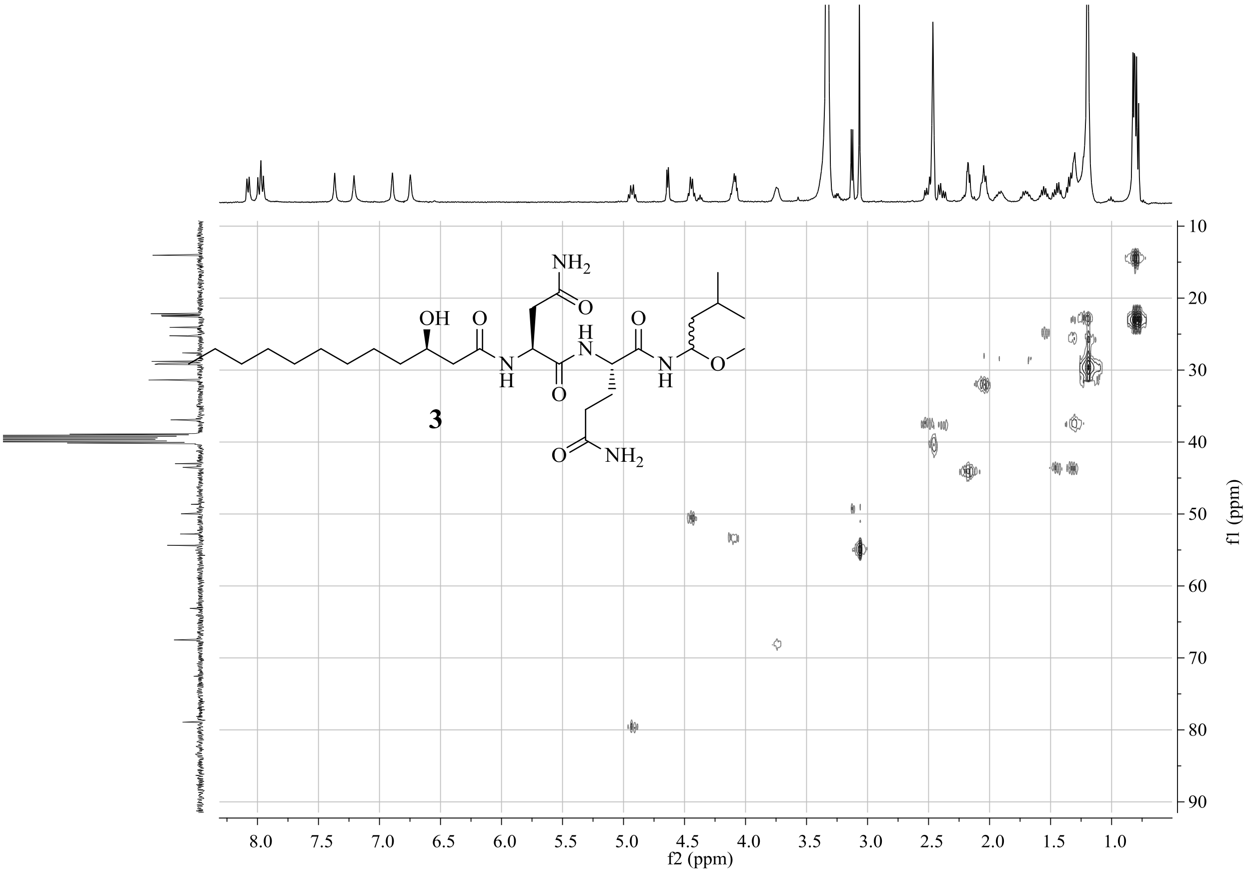

Figure SP3. I: HMBC spectrum of **3** in DMSO-d_6.

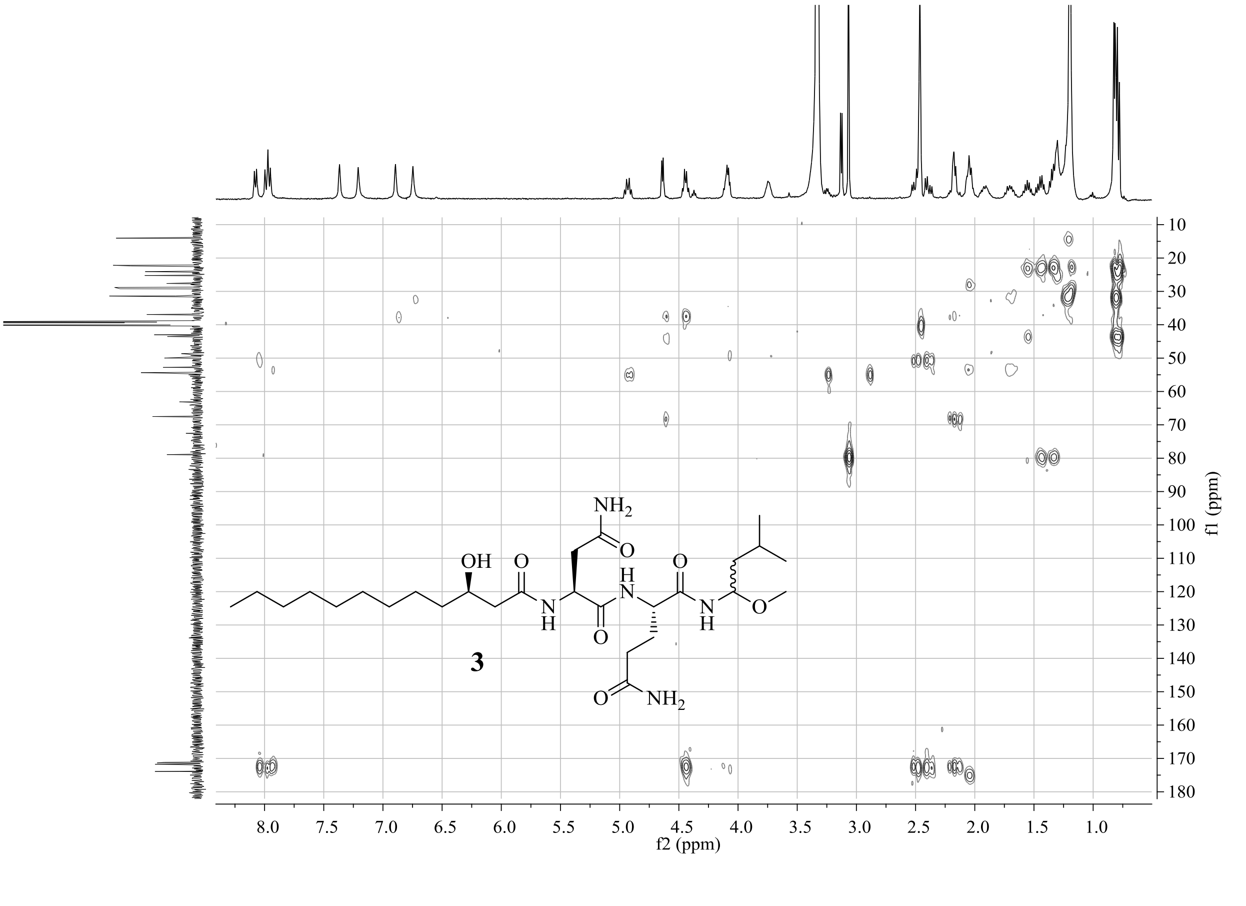

Figure SP4. A: Positive (A) and negative (B) ESIMS spectra of **4**.

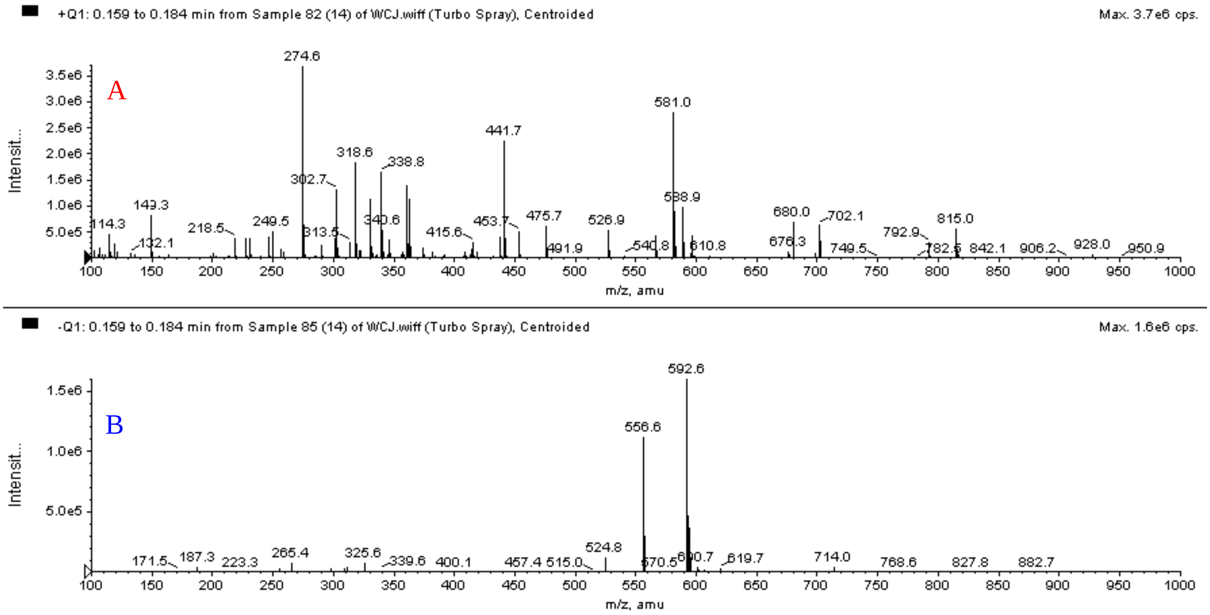

Figure SP4. B: Positive HRESIMS spectrum of **4**.

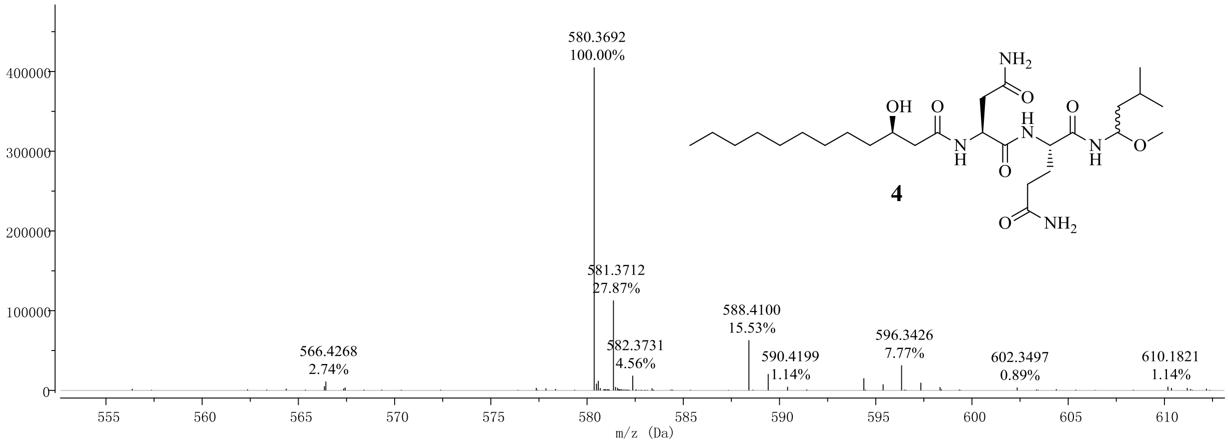

Figure SP4. C: IR spectrum of **4**.

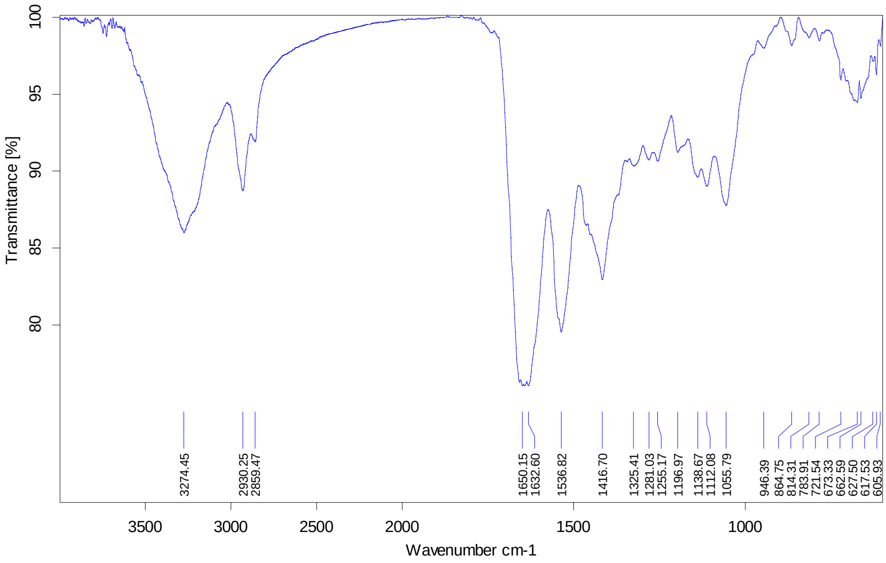

Figure SP4. D: 400 MHz ^{1}H NMR spectrum of **4** in DMSO-d_6.

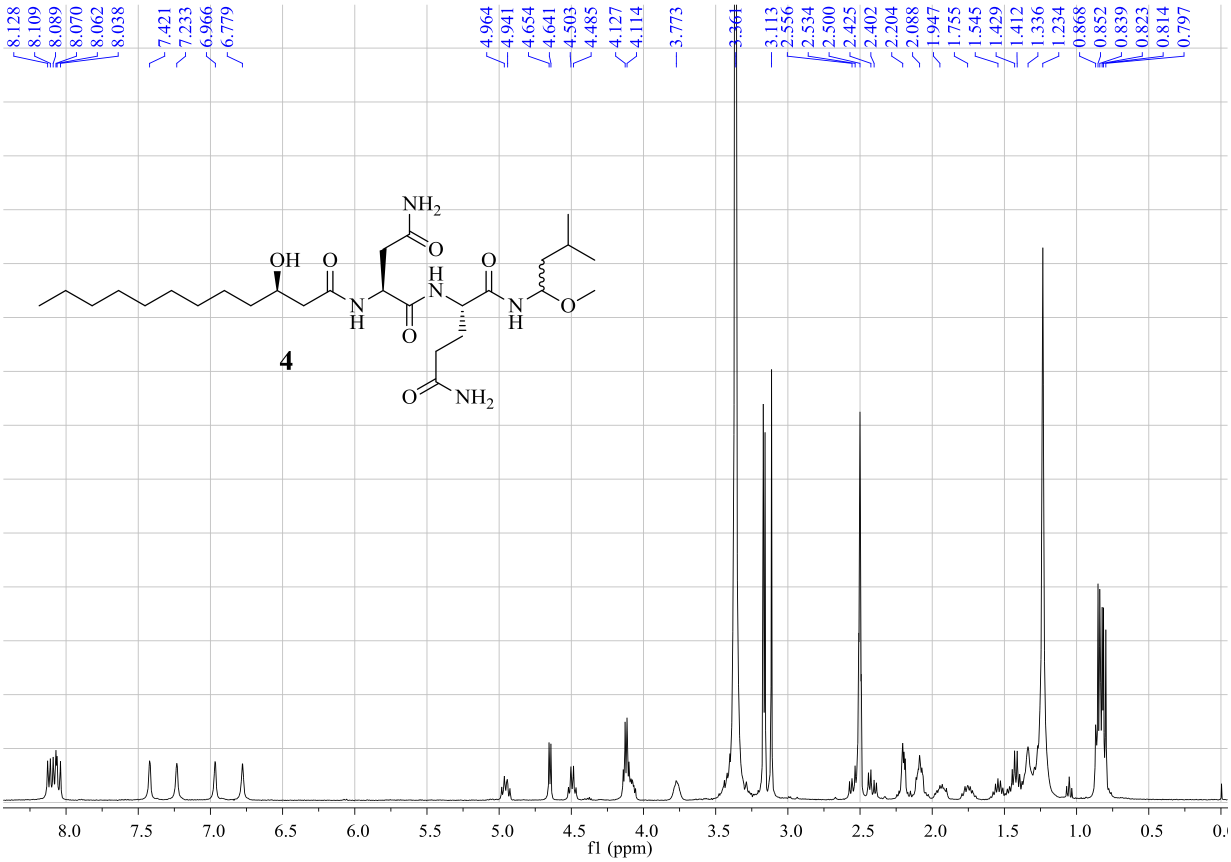

Figure SP4. E: 100 MHz ^{13}C NMR spectrum of **4** in DMSO-d_6.

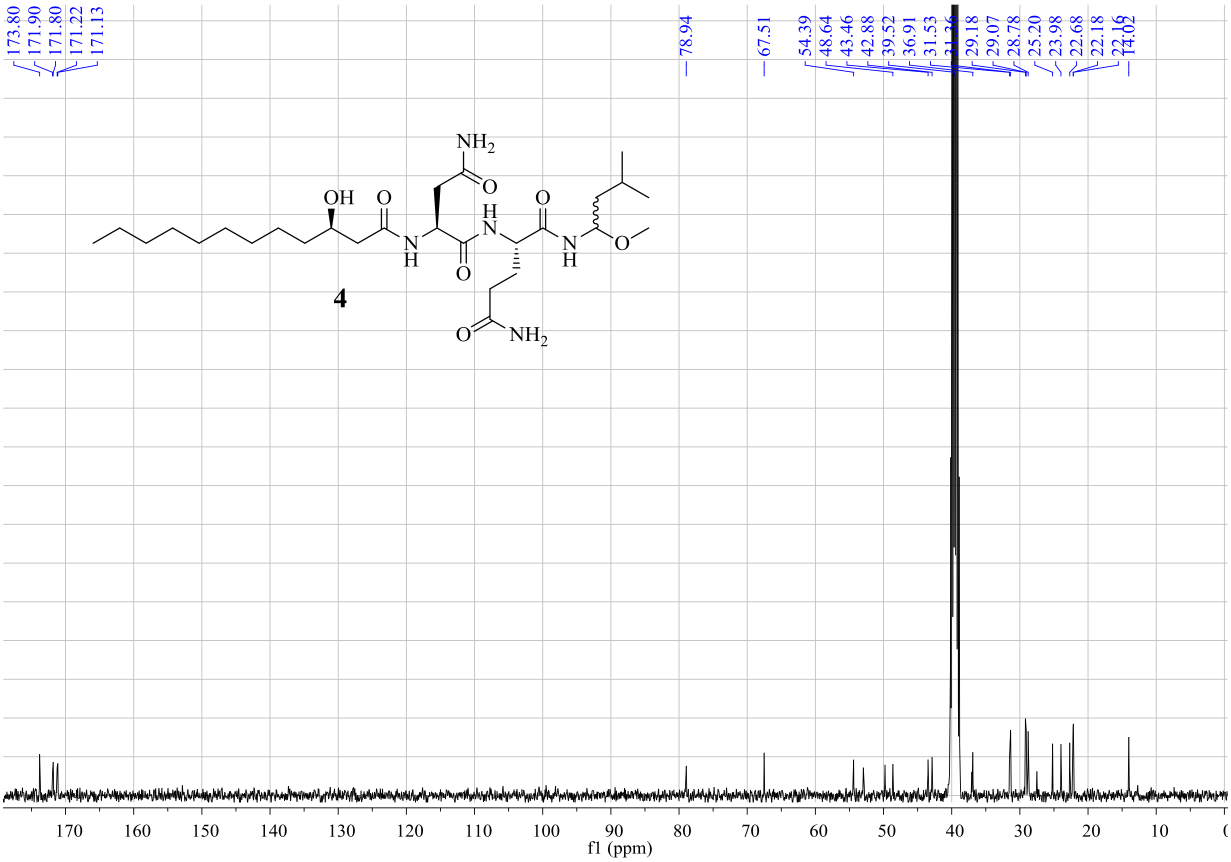

Figure SP4. F: DEPT spectra of **4** in DMSO-d_6.

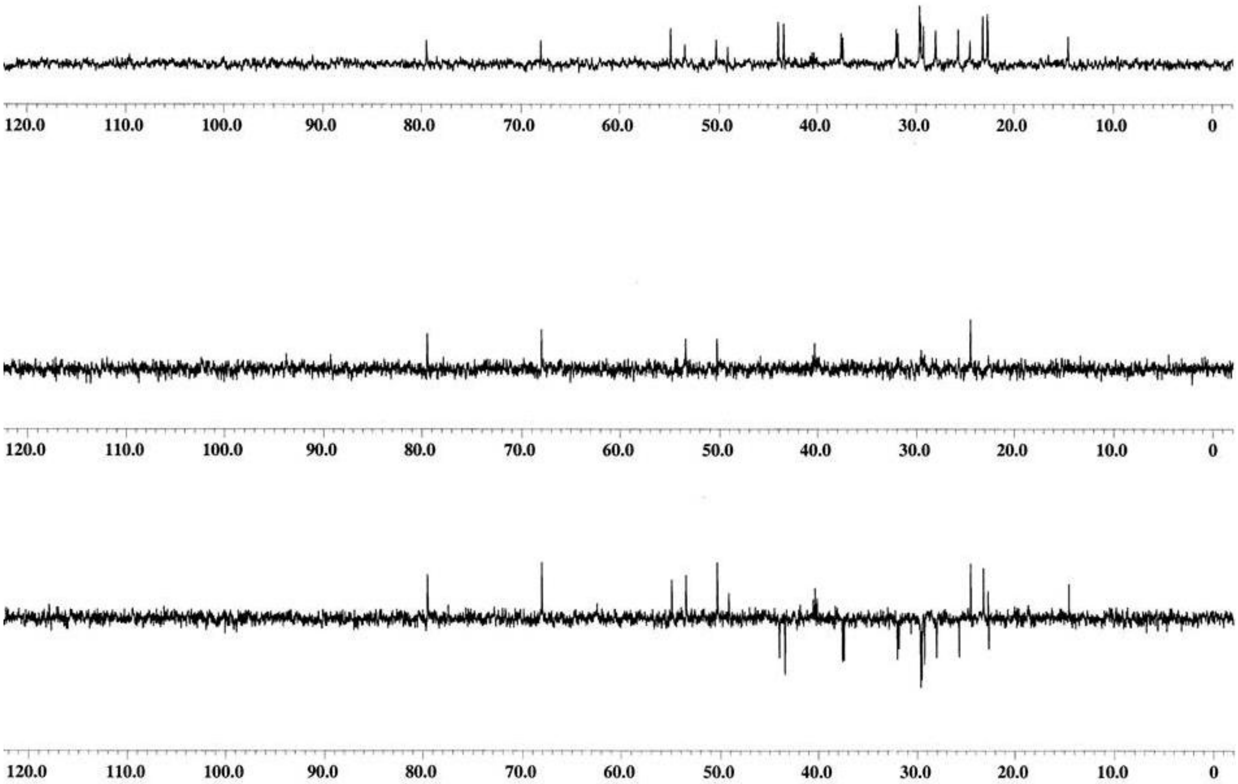

Figure SP4. G: ^{1}H-^{1}H COSY spectrum of **4** in DMSO-d_6.

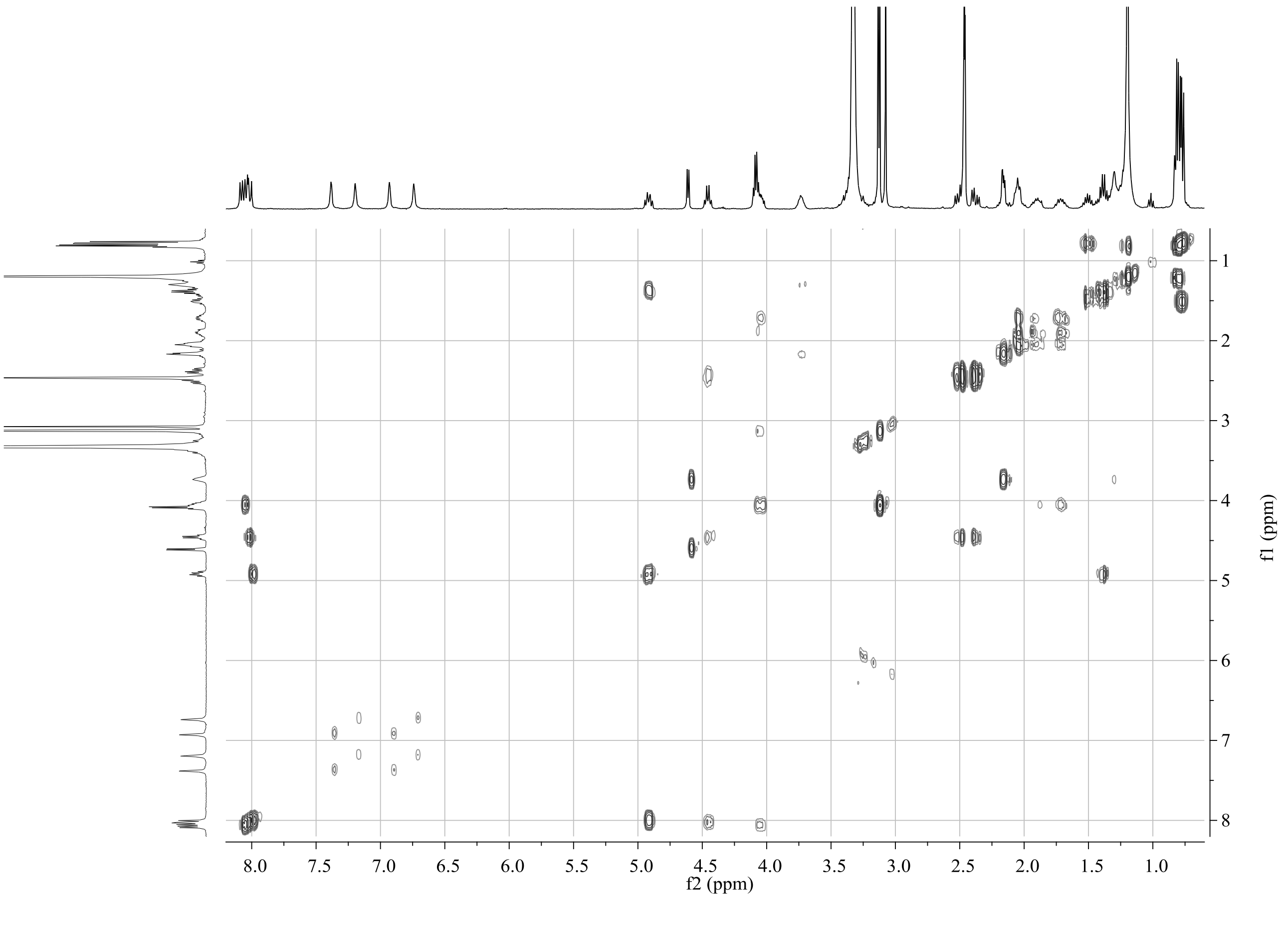

Figure SP4. H: HMQC spectrum of **4** in DMSO-d_6.

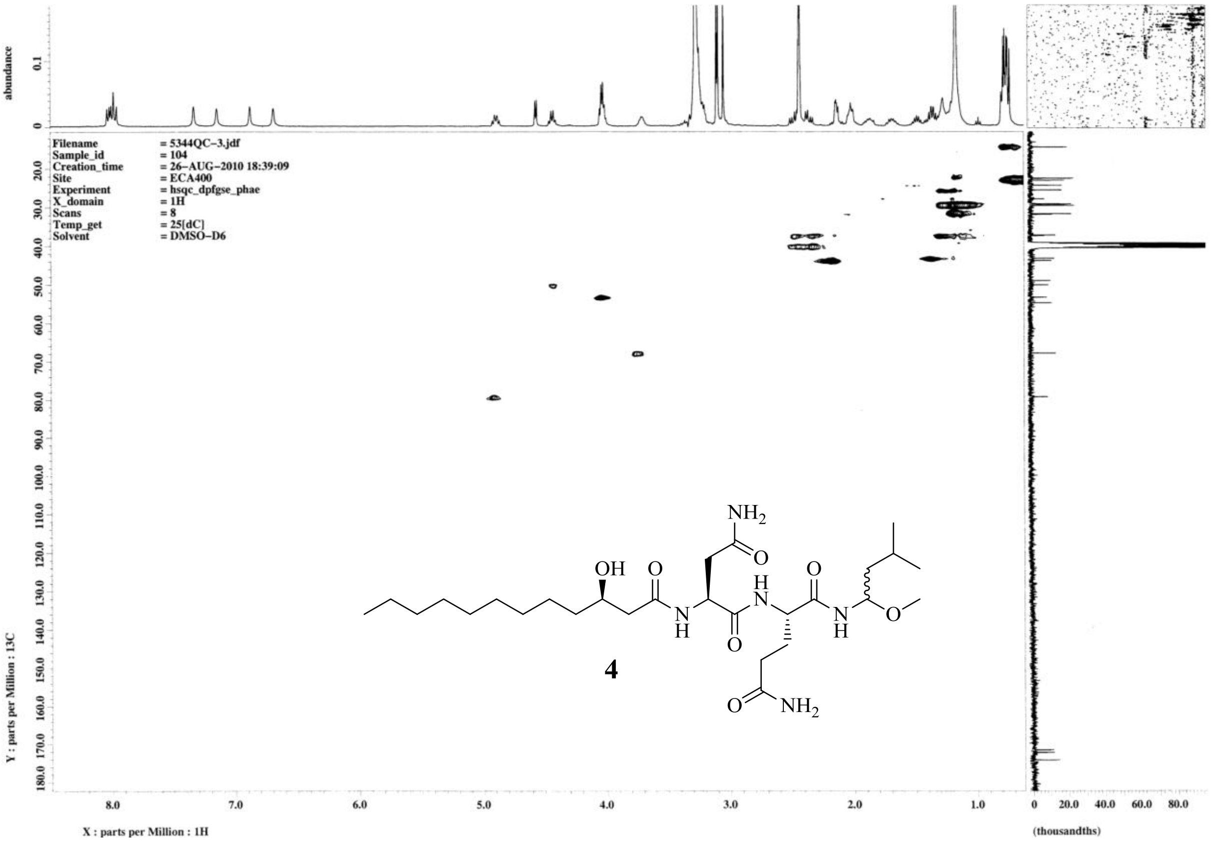

Figure SP4. I: HMBC spectrum of **4** in DMSO-d_6.

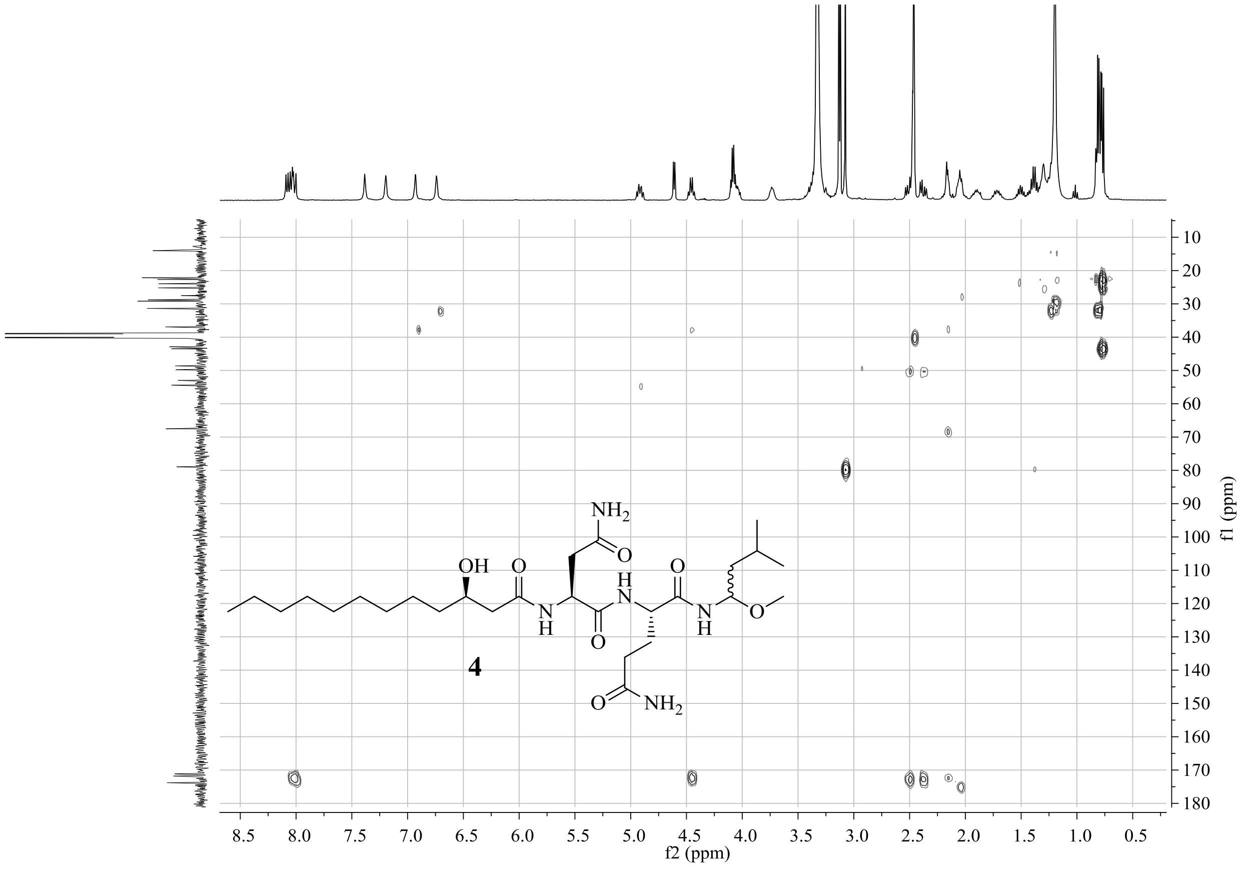

Figure SP5. A: Positive (A) and negative (B) ESIMS spectra of **5**.

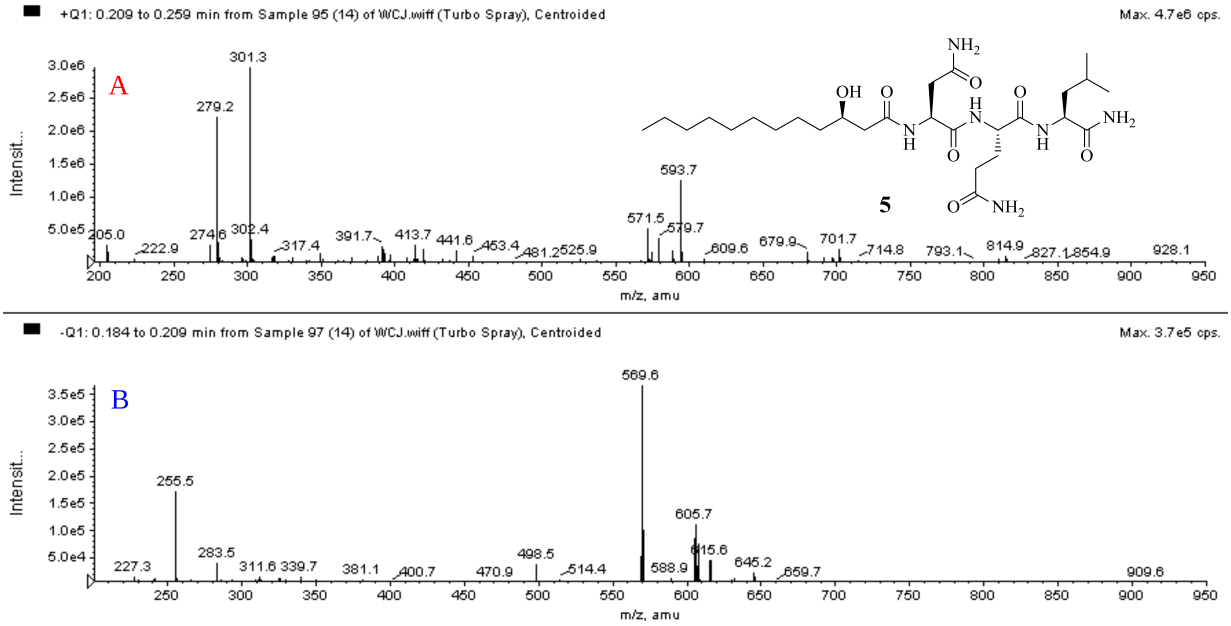

Figure SP5. B: Positive HRESIMS spectrum of **5**.

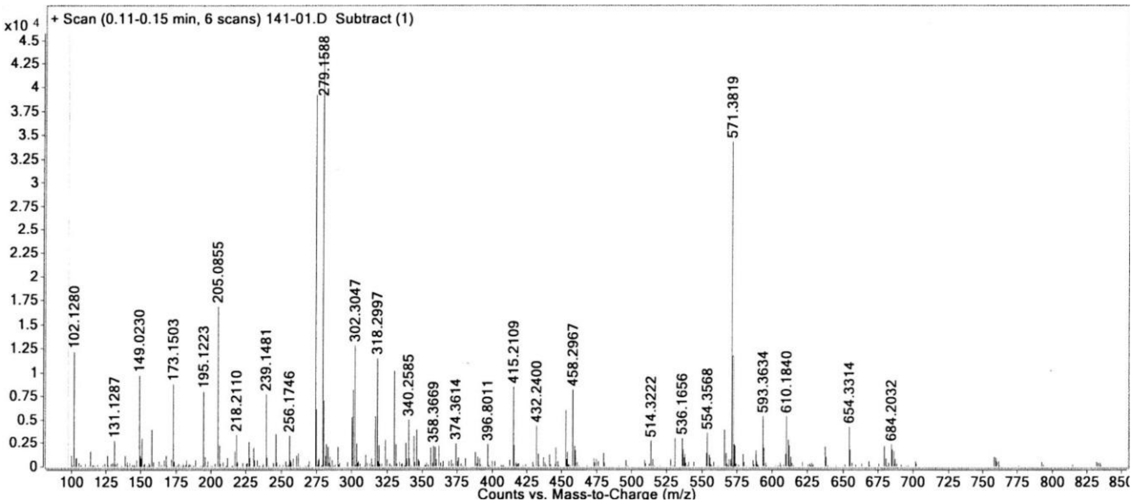

Figure SP5. C: IR spectrum of **5**.

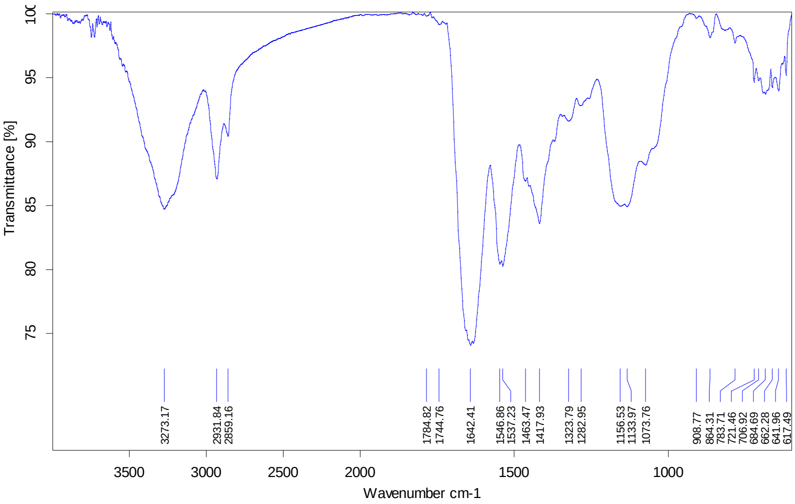

Figure SP5. D: 600 MHz ^{1}H NMR spectrum of **5** in DMSO-d_6.

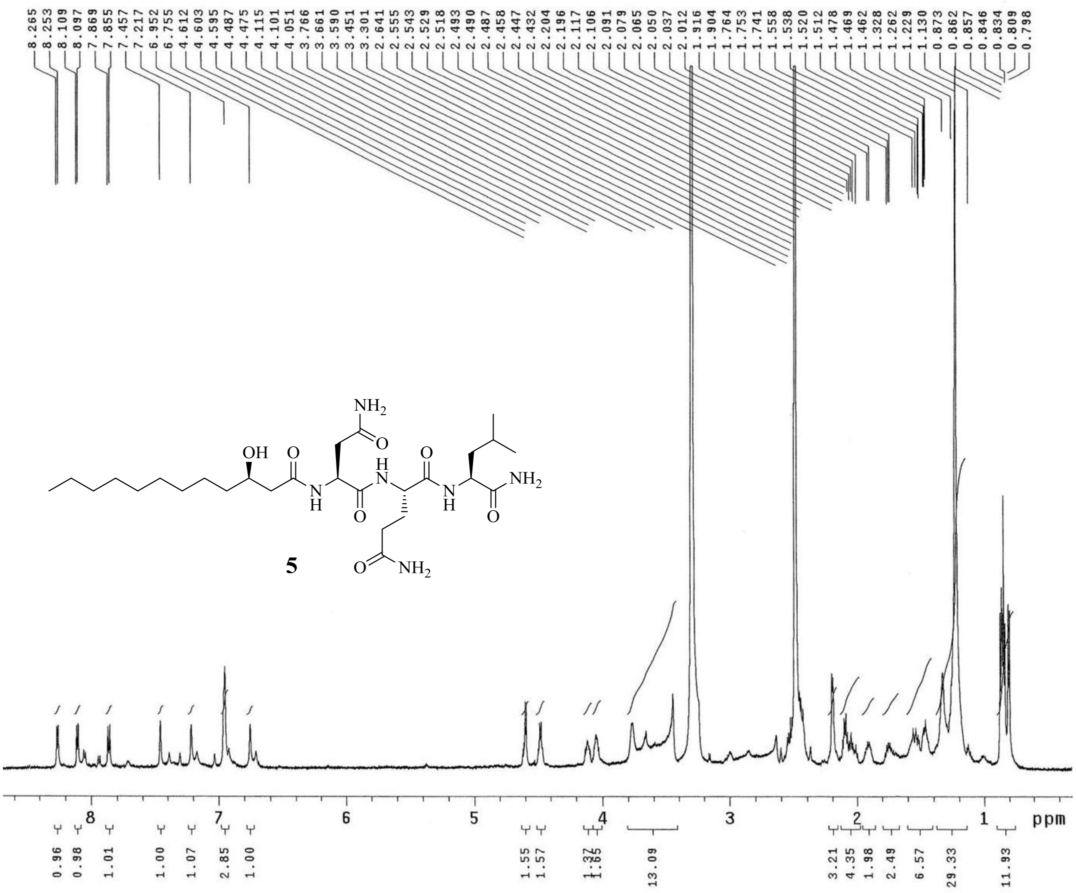

Figure SP5. E: 150 MHz ^{13}C NMR spectrum of **5** in DMSO-d_6.

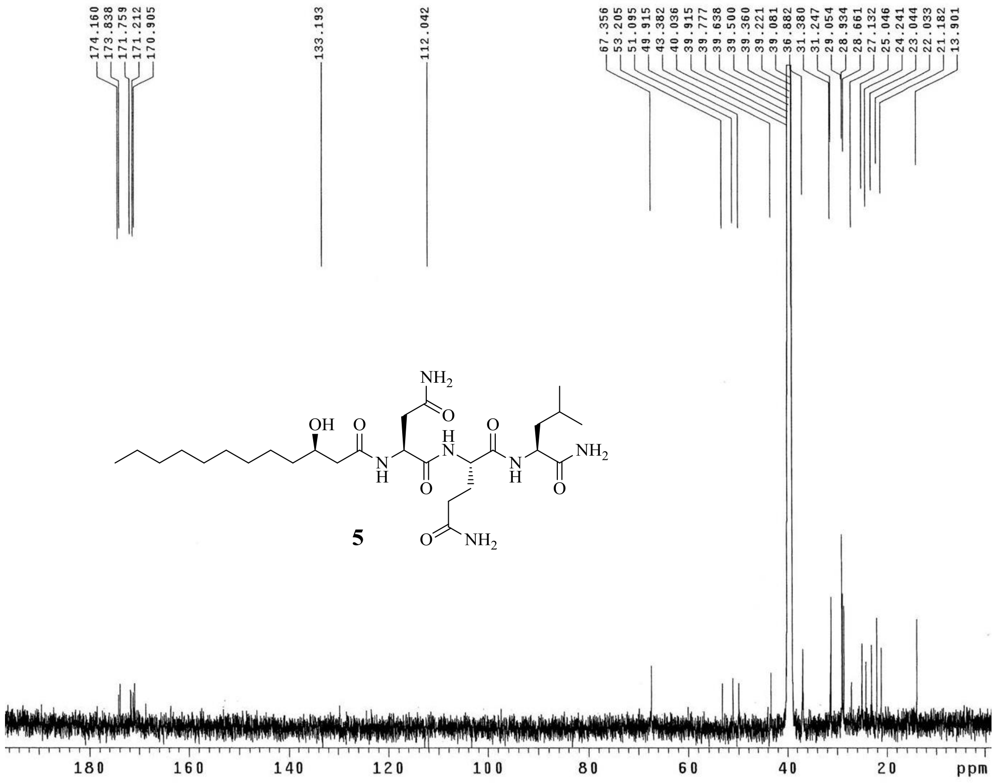

Figure SP6. A: Positive (A) and negative (B) ESIMS spectra of **6**.

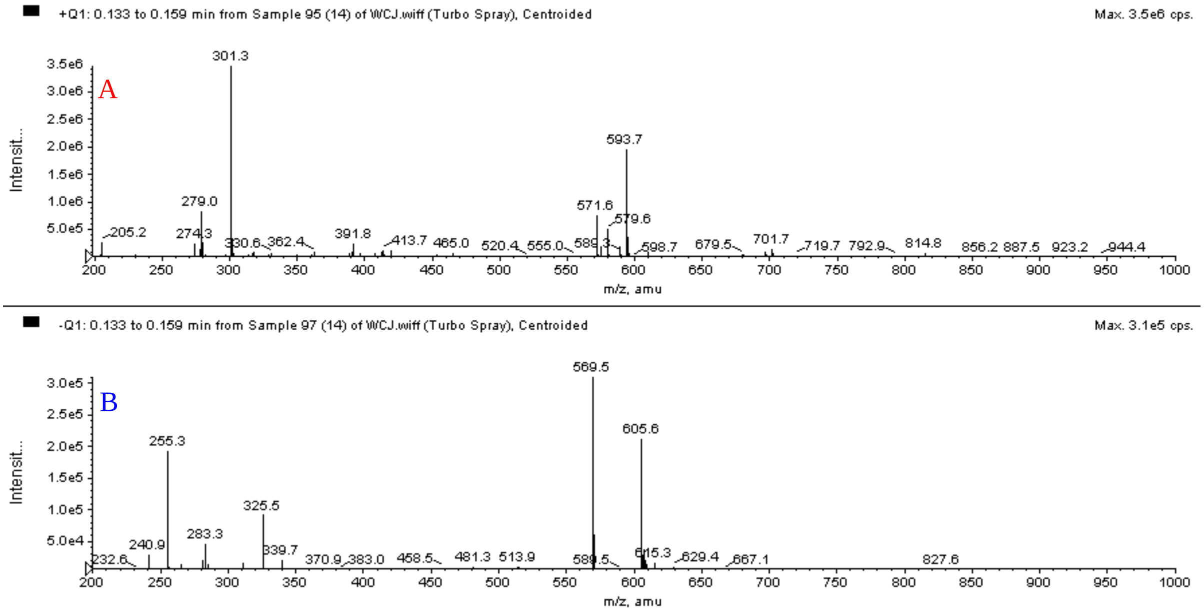

Figure SP6. B: Positive HRESIMS spectrum of **6**.

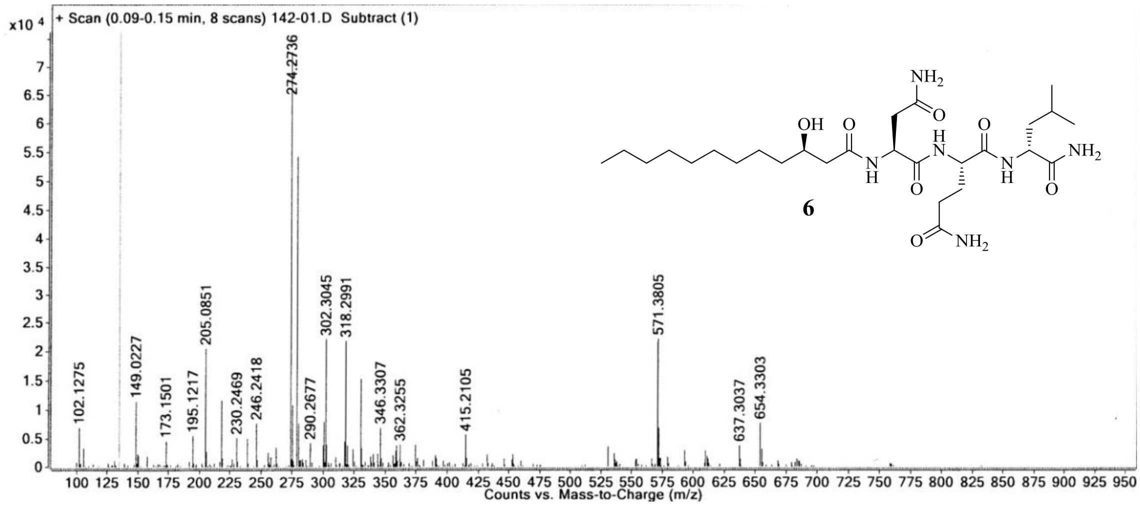

Figure SP6. C: IR spectrum of **6**.

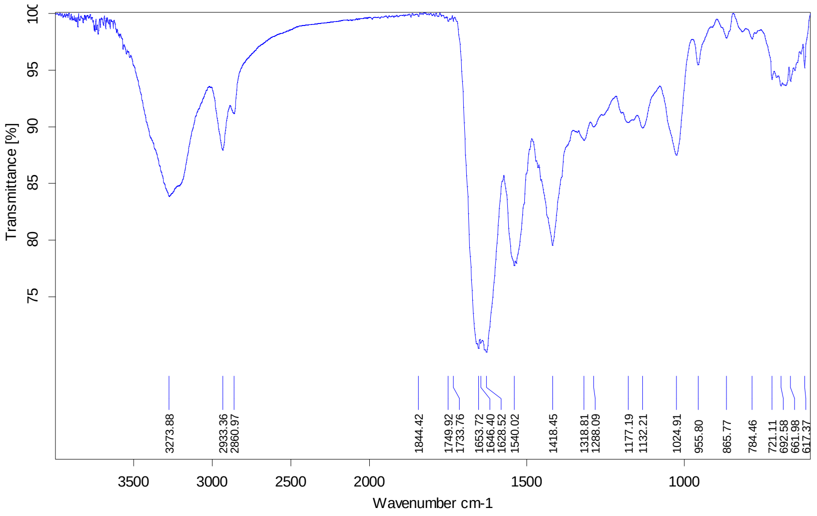

Figure SP6. D: 600 MHz ^{1}H NMR spectrum of **6** in DMSO-d_6.

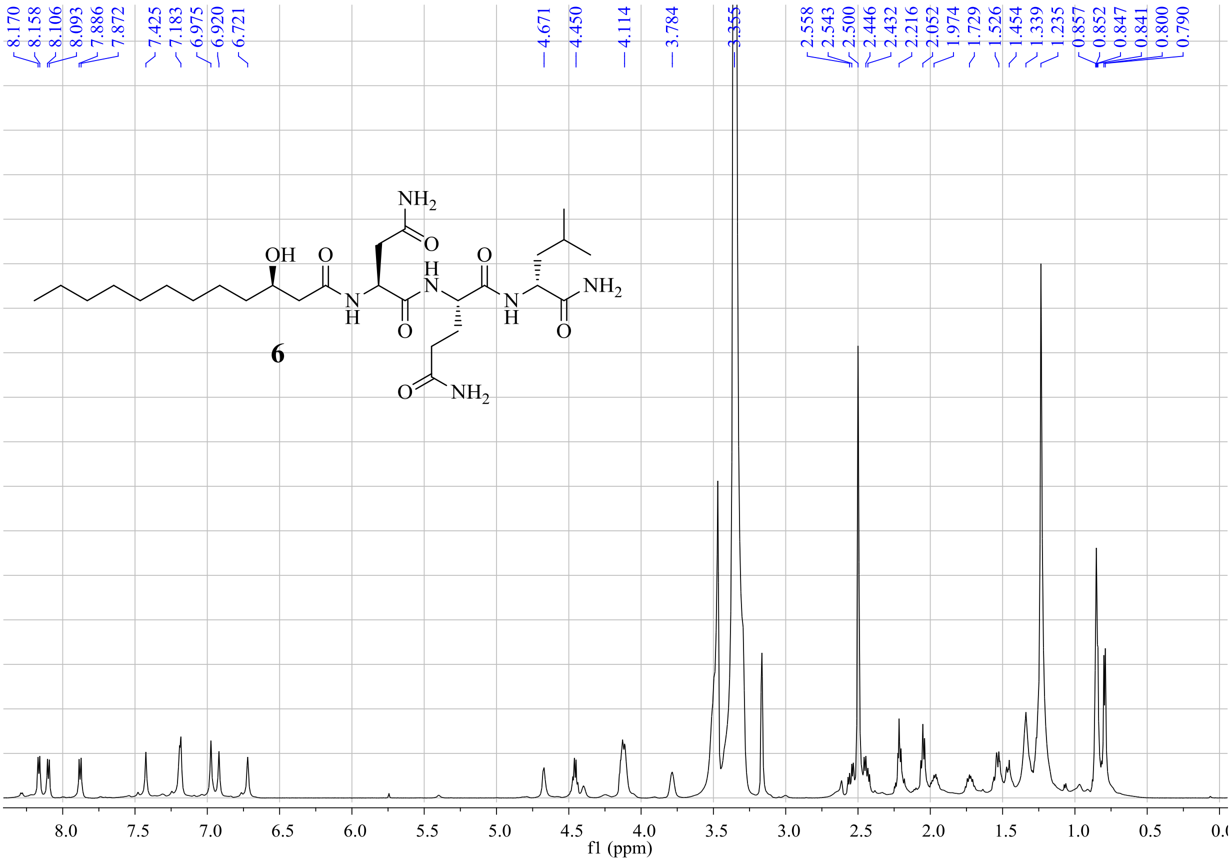

Figure SP6. E: 150 MHz ^{13}C NMR spectrum of **6** in DMSO-d_6.

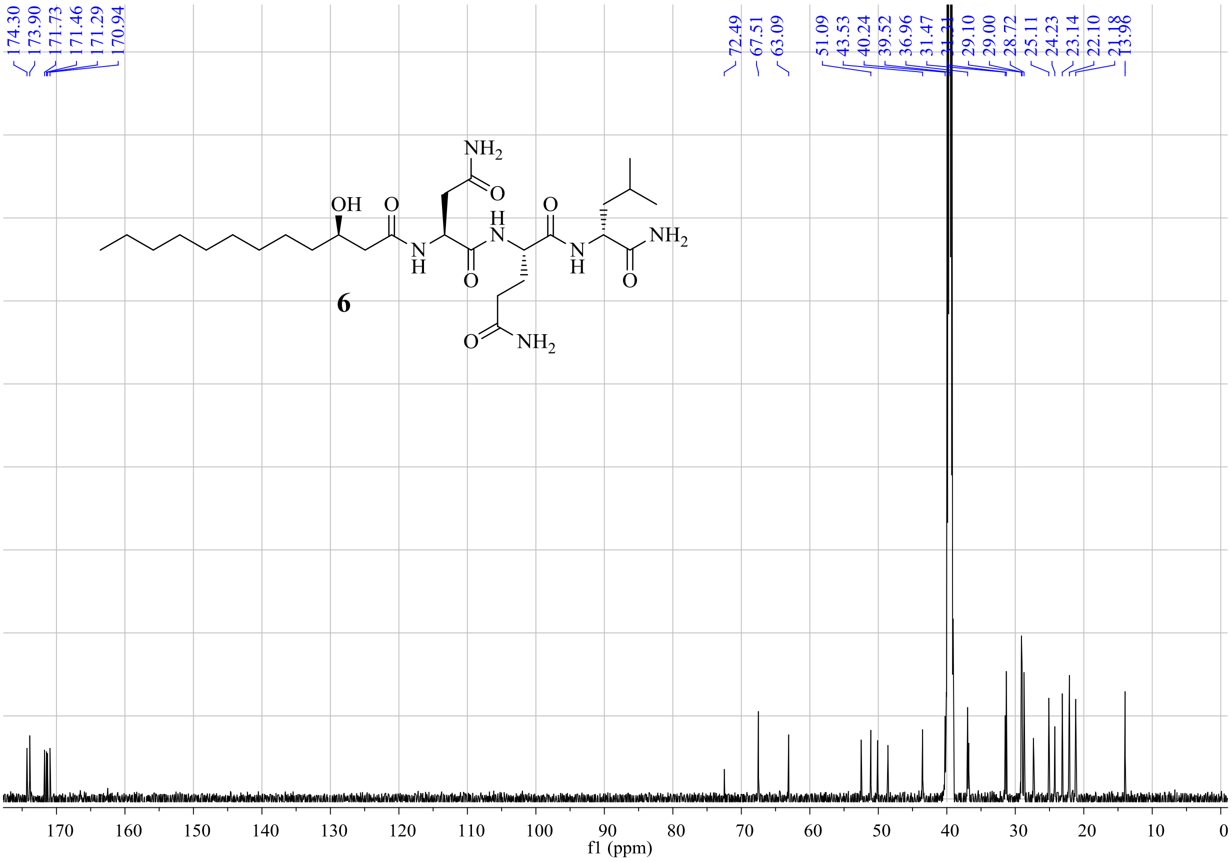

Figure SP6. F: DEPT spectra of **6** in DMSO-d_6.

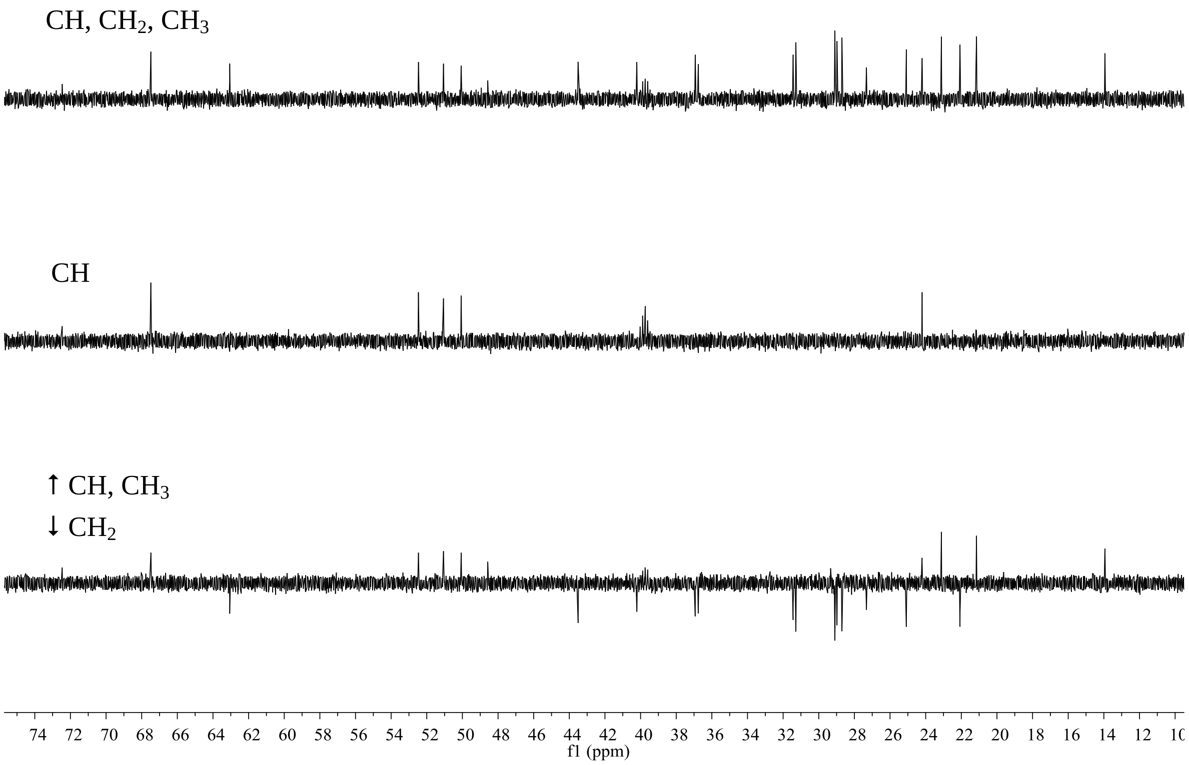

Figure SP6. G: ^{1}H-^{1}H COSY spectrum of **6** in DMSO-d_6.

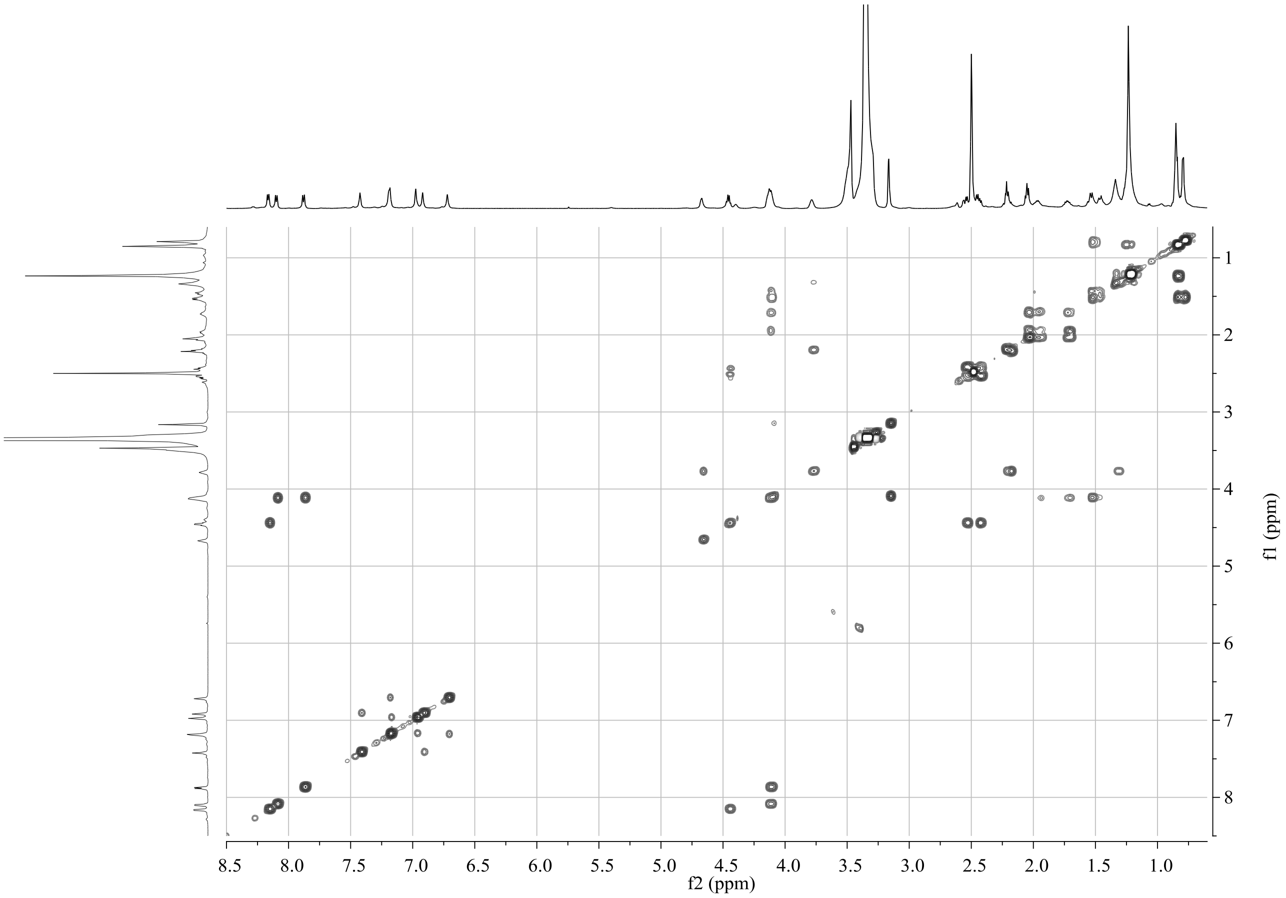

Figure SP6. H: HMQC spectrum of **6** in DMSO-d_6.

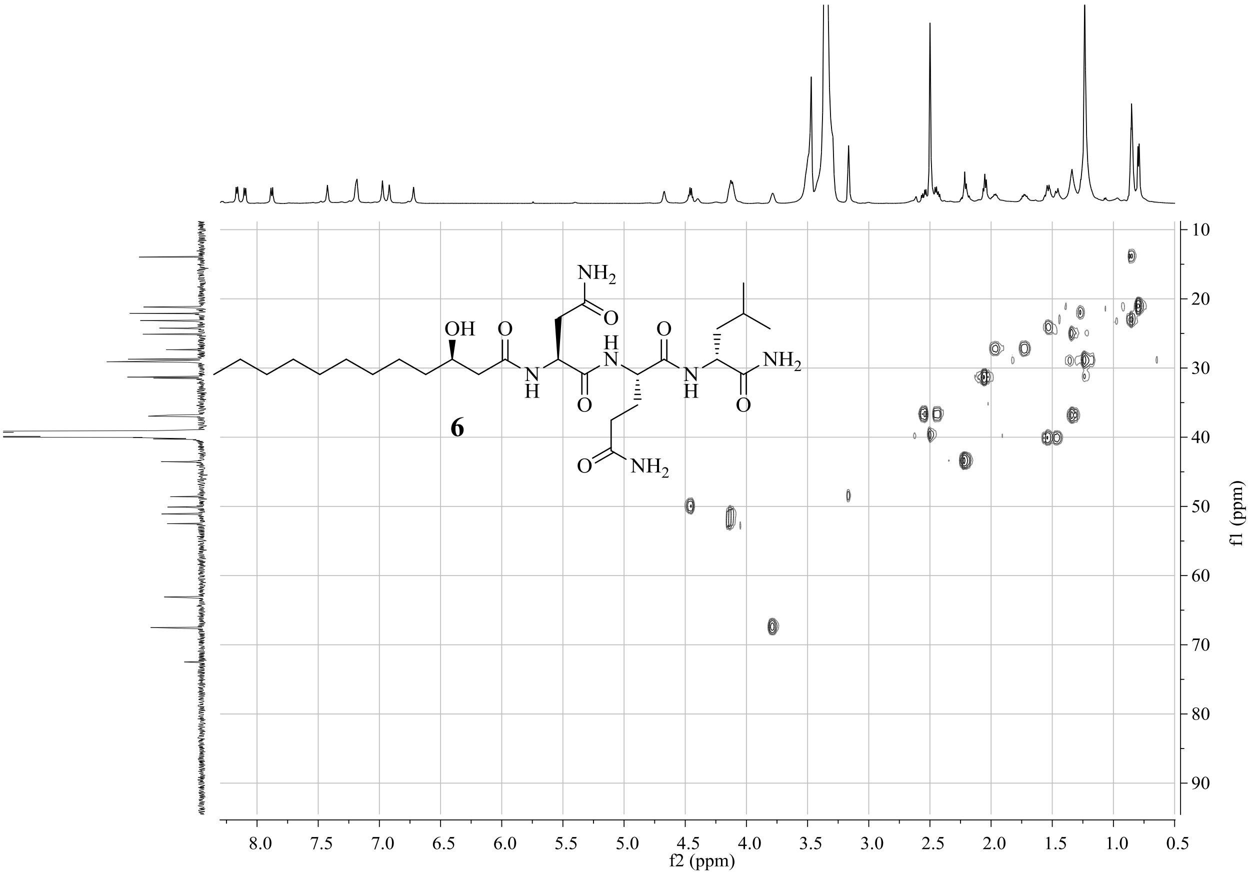

Figure SP6. I: HMBC spectrum of **6** in DMSO-d_6.

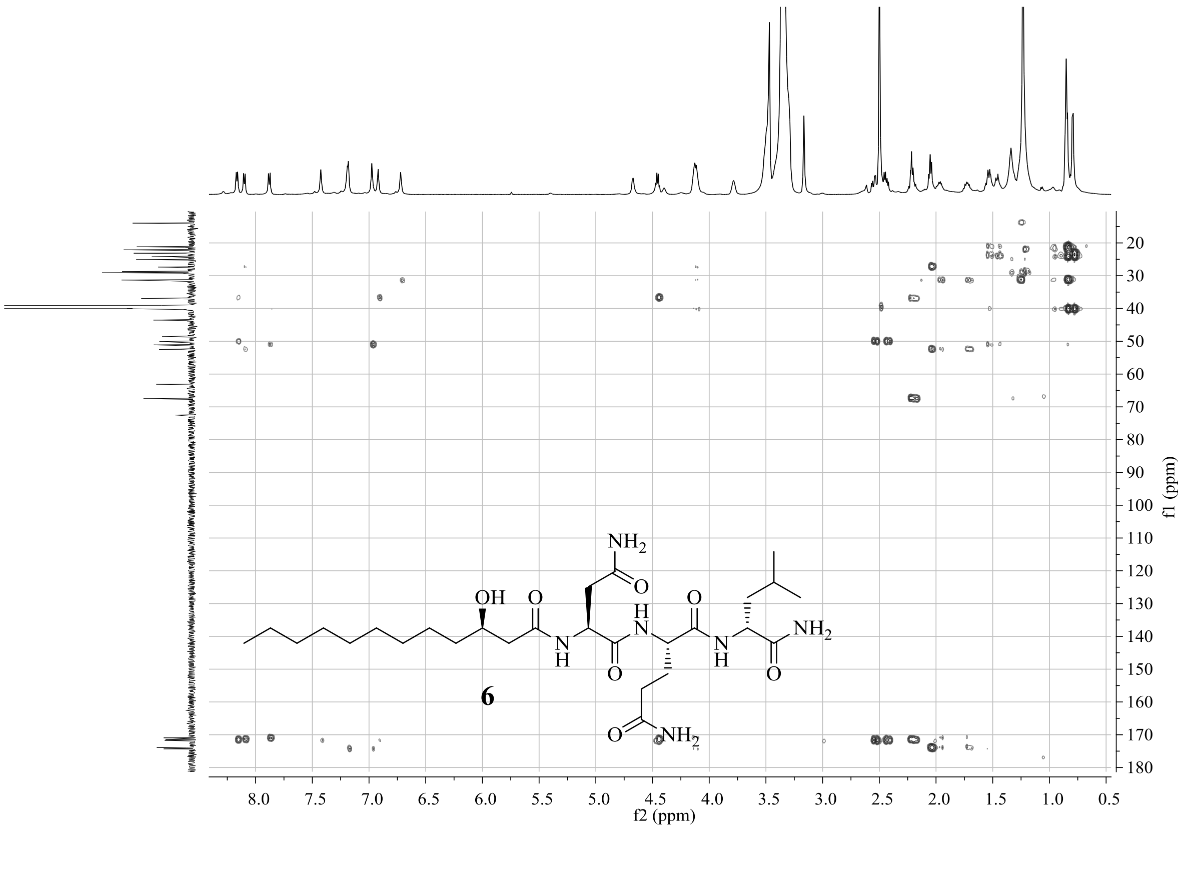

Figure SP7. A: Positive (A) and negative (B) ESIMS spectra of **7**.

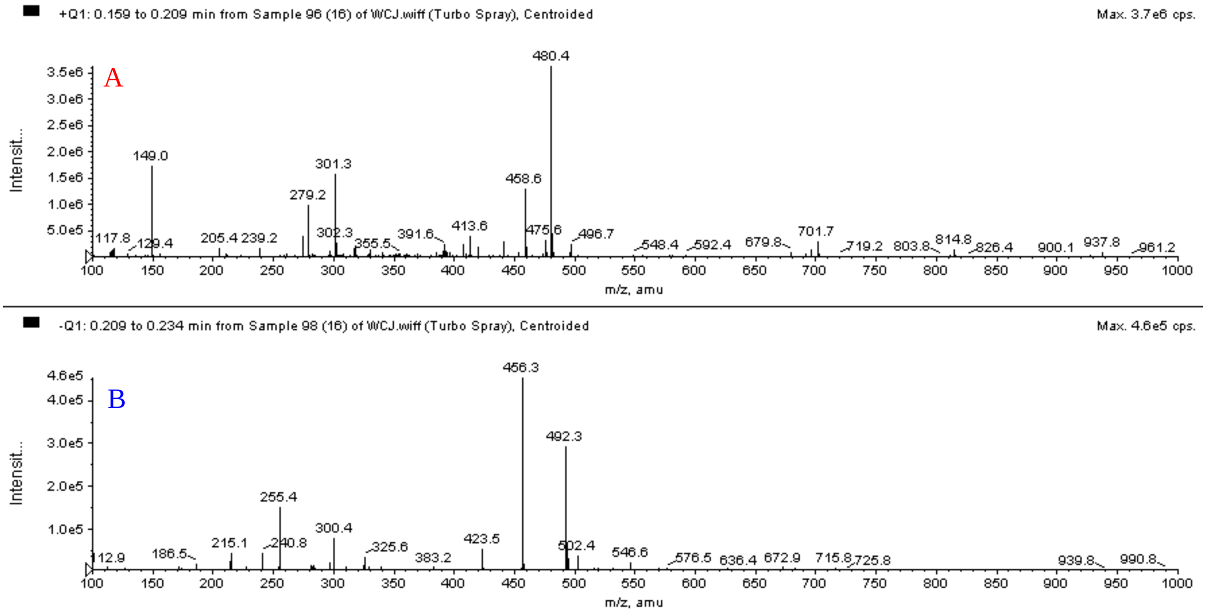

Figure SP7. B: Positive HRESIMS spectrum of **7**.

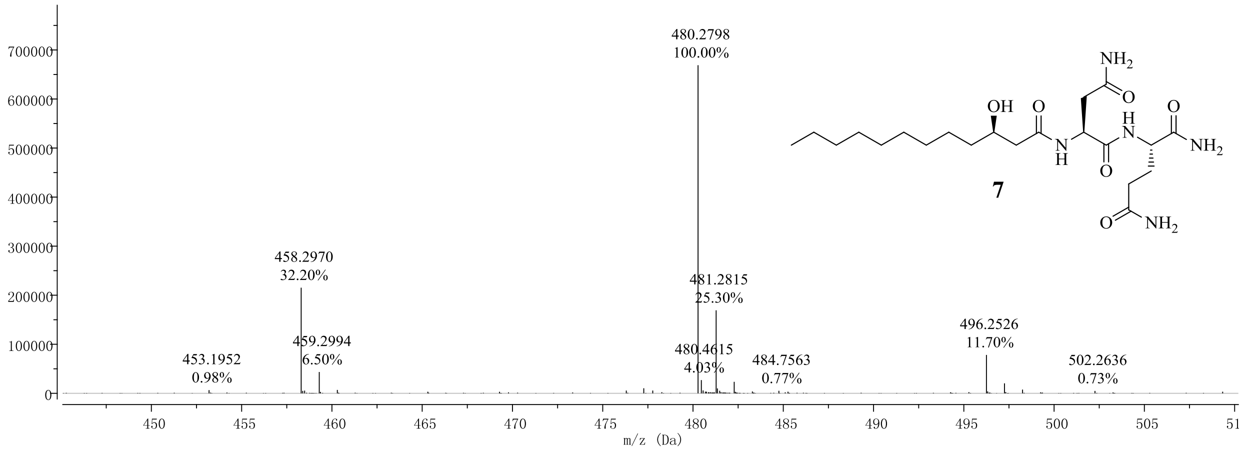

Figure SP7. C: IR spectrum of **7**.

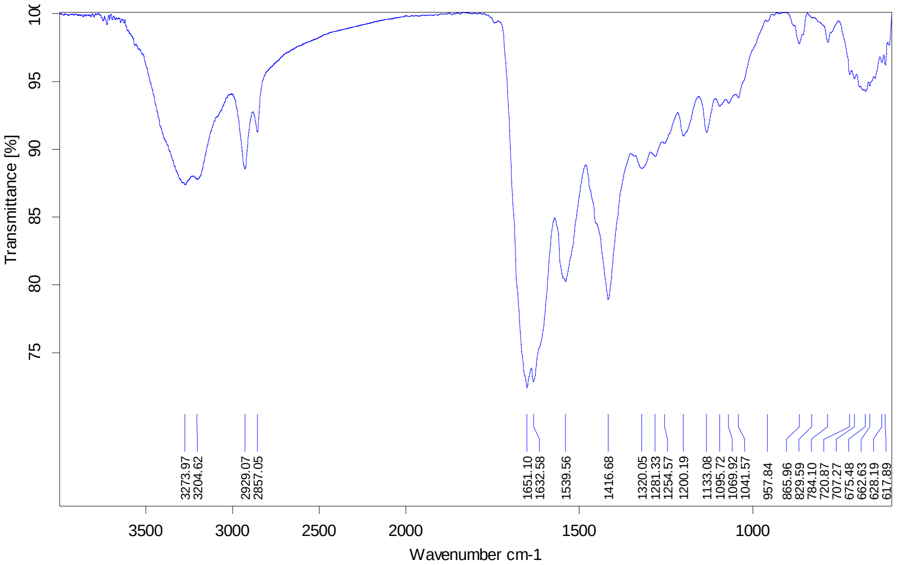

Figure SP7. D: 400 MHz ^{1}H NMR spectrum of **7** in DMSO-d_6.

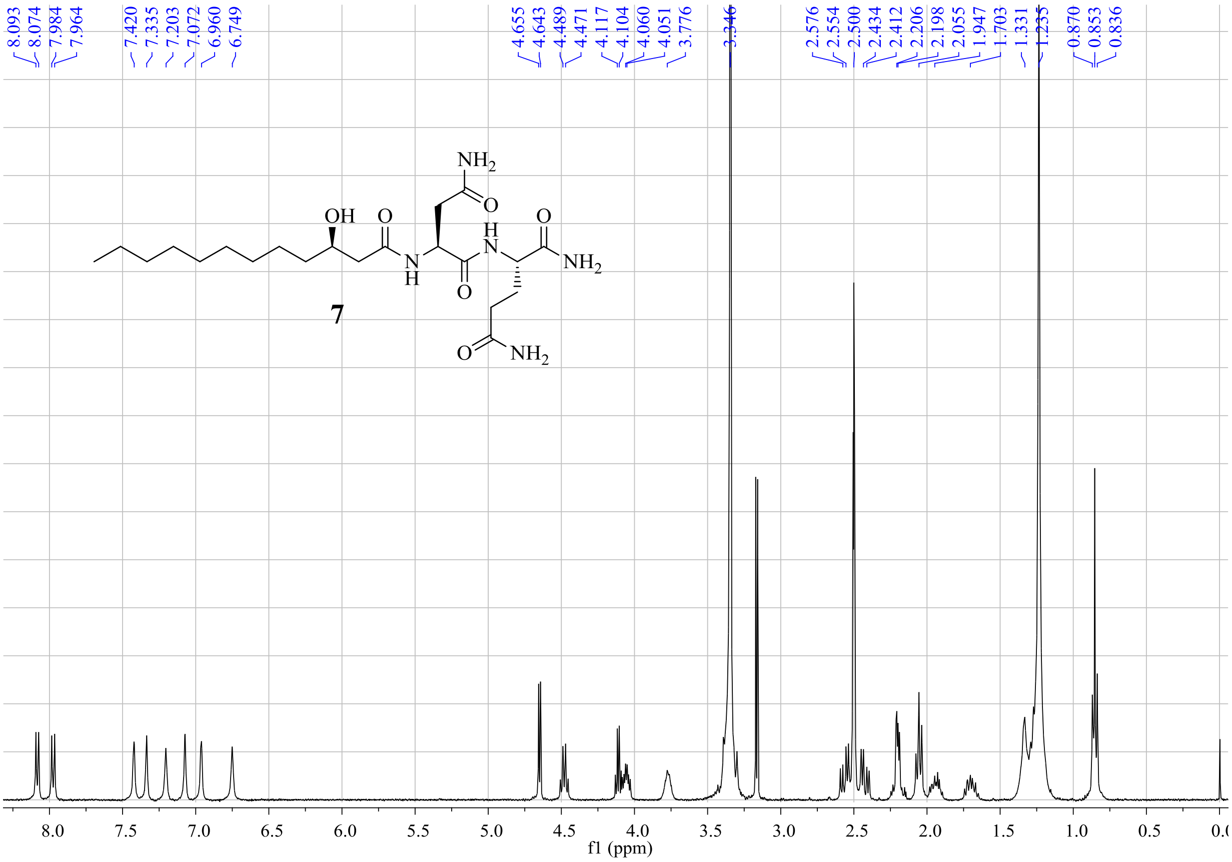

Figure SP7. E: 100 MHz ^{13}C NMR spectrum of **7** in DMSO-d_6.

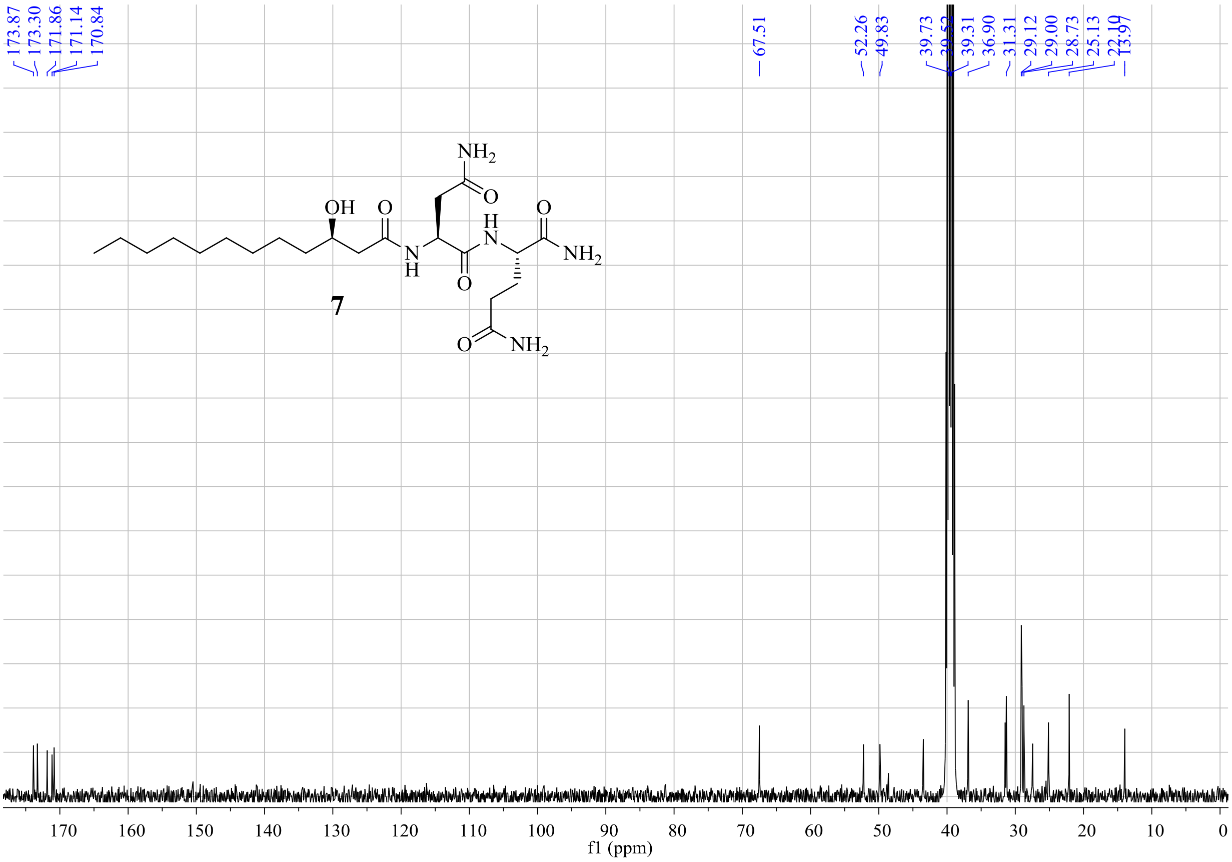

Figure SP7. F: DEPT spectra of **7**.

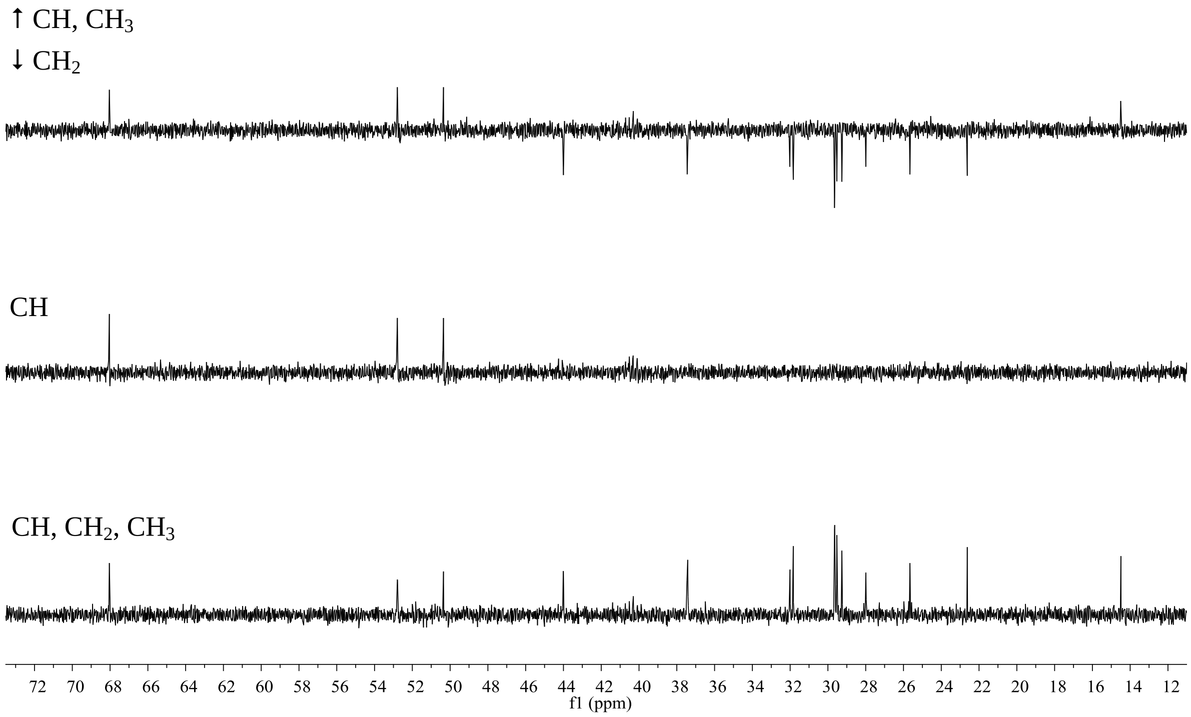

Figure SP7. G: ^1H-^1H COSY spectrum of **7**.

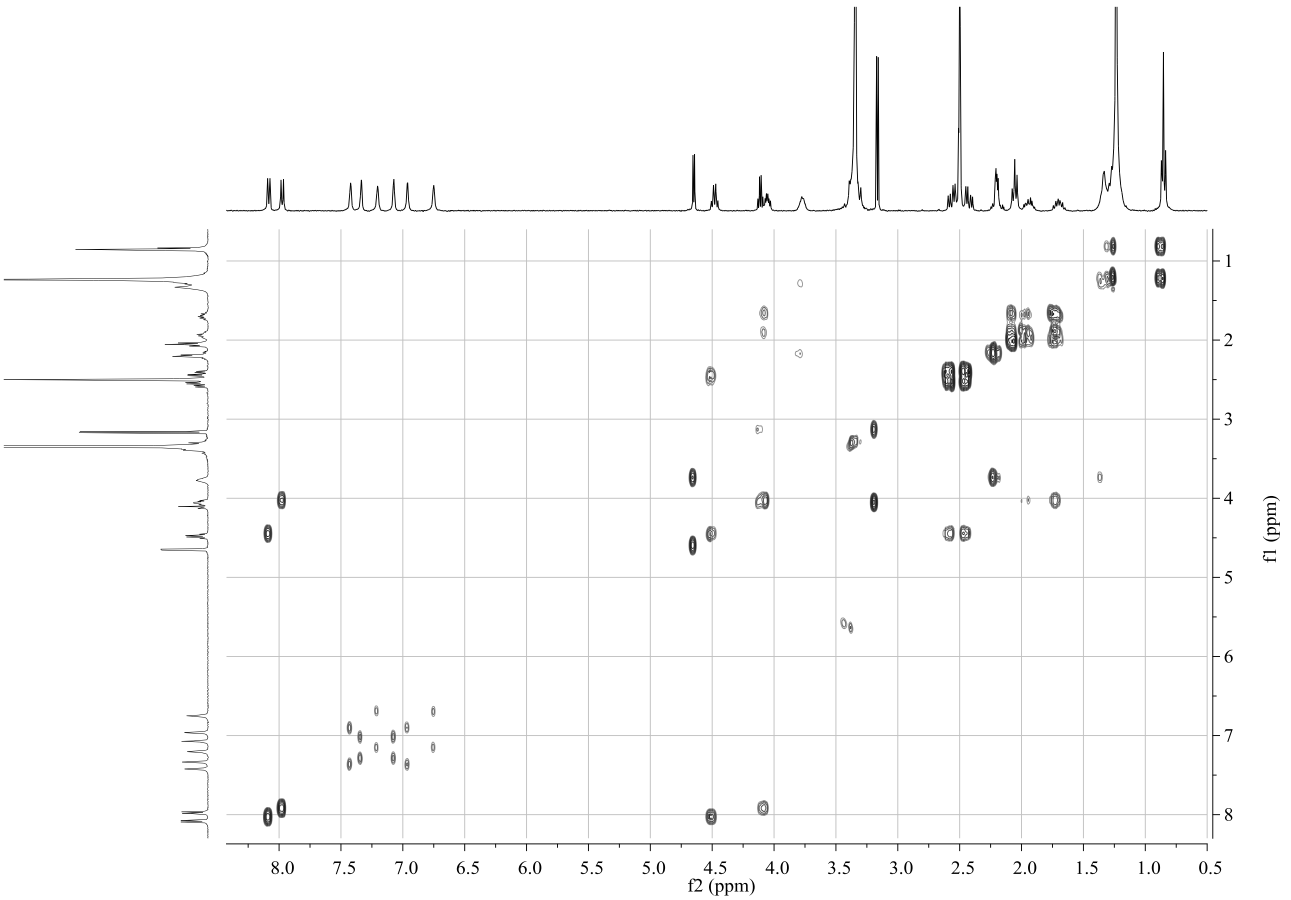

Figure SP7. H: HMQC spectrum of **7**.

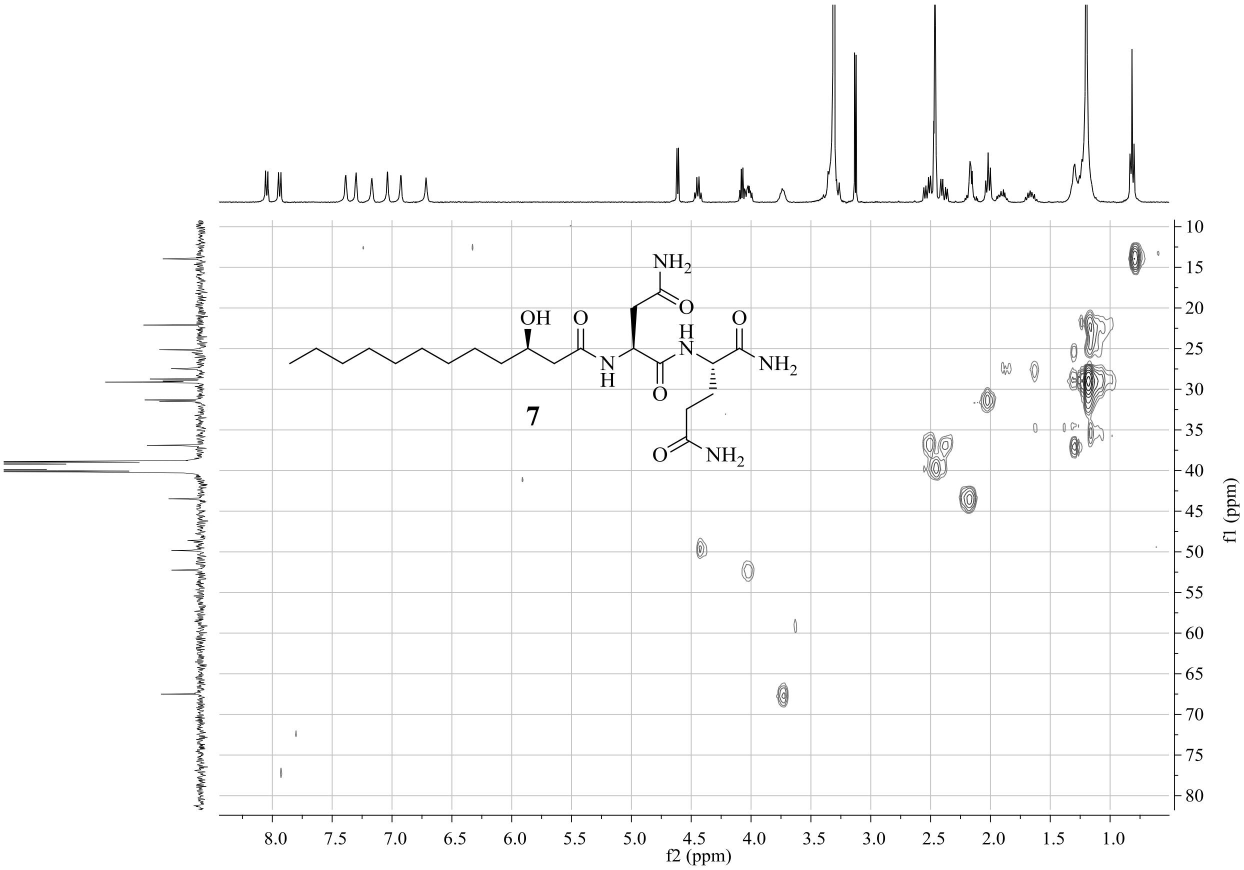

Figure SP7. I: HMBC spectrum of **7**.

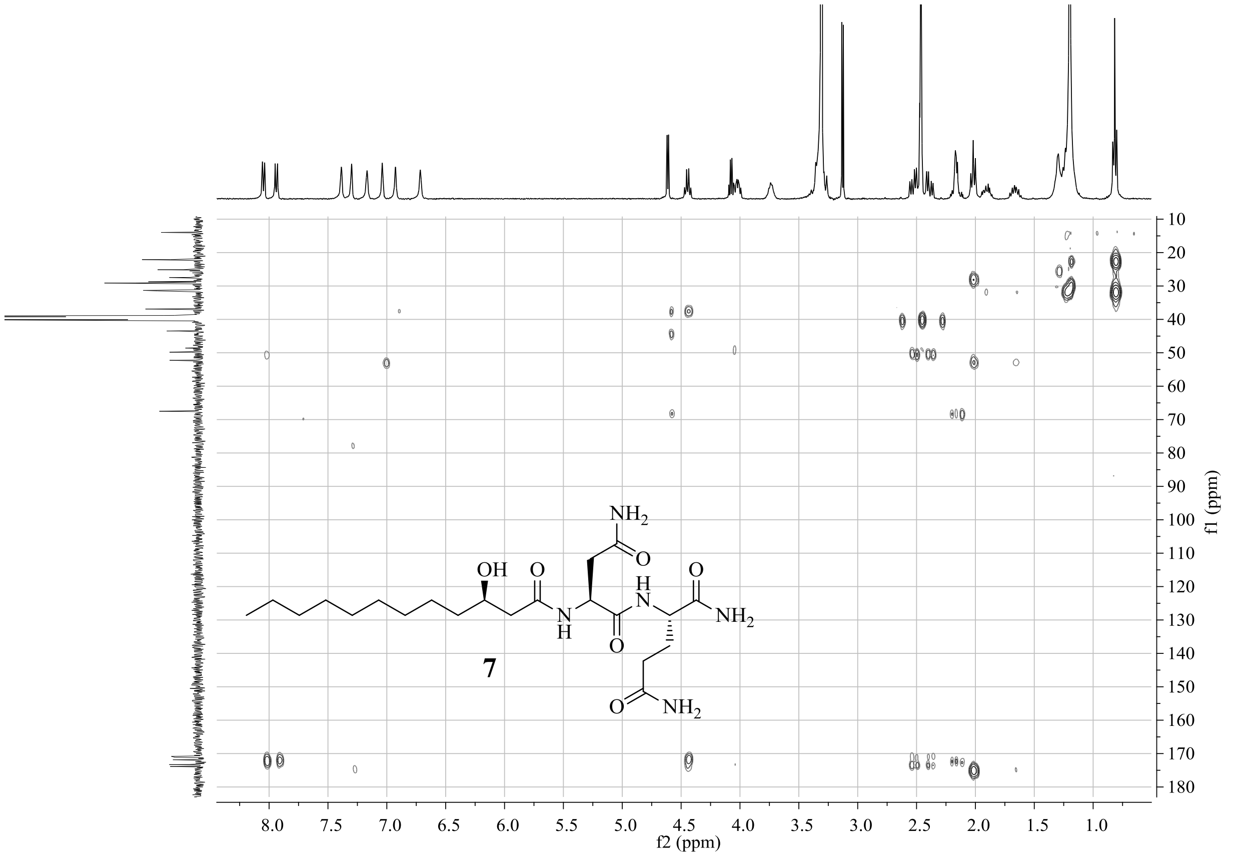

Supplement: Supplementary File 1 — Supplementary Information (PDF, 5380 KB) [file marinedrugs-12-01815-s001.pdf]
